# A two-stage framework for enhancing crsyptocurrency portfolio performance: Integrating credibilistic CVaR criterion with a novel asset preselection approach

**DOI:** 10.1371/journal.pone.0325973

**Published:** 2025-07-21

**Authors:** Hossein Ghanbari, Sina Tavakoli, Mostafa Shabani, Emran Mohammadi, Seyed Jafar Sadjadi, Ronald Ravinesh Kumar

**Affiliations:** 1 Department of Industrial Engineering, Iran University of Science and Technology, Tehran, Iran; 2 Department of Economics and Finance, The Business School, RMIT University, Saigon South Campus, Ho Chi Minh City, Vietnam; Aalto University, FINLAND

## Abstract

In an increasingly diverse investment landscape, the cryptocurrency market has emerged as a compelling option, offering the potential for high returns, diversification opportunities, and significant liquidity. However, the inherent volatility and regulatory uncertainties of this market present substantial risks, underscoring the need for a well-structured investment strategy. Among the various strategies available, portfolio optimization has become a dynamic and evolving area of focus in finance. Despite advancements in financial modeling, traditional portfolio optimization models often fall short, as uncertainty remains a fundamental characteristic of capital markets. To address this challenge, this paper integrates credibility theory with the Conditional Value-at-Risk (CVaR) framework, harnessing their combined strengths in modeling downside risk and managing uncertainty. Nevertheless, relying solely on this model may not be sufficient for achieving optimal investment outcomes, as portfolio optimization models often neglect the crucial step of selecting high-quality assets. This highlights the essential need for a robust pre-selection process. To tackle this issue, this paper introduces a novel pre-selection framework based on Multi-Attribute Decision Making (MADM) methods. Acknowledging that different MADM approaches can yield varying results—which creates uncertainty regarding the most reliable method—this research proposes a systematic framework for asset evaluation. By considering these factors, this paper proposes a two-stage framework for enhancing cryptocurrency portfolio performance. Stage 1, involves establishing comprehensive performance criteria for cryptocurrencies and employing a novel method for asset pre-selection. Stage 2 focuses on optimizing the selected assets using a credibilistic CVaR model, while considering practical constraints from real-world investment scenarios. The results of this two-stage framework demonstrate its effectiveness in constructing well-diversified and efficient portfolios, addressing both the challenges of asset pre-selection and the complexities associated with uncertainty. By integrating these methodologies, investors can navigate the risks associated with cryptocurrency investments more effectively while maximizing potential returns.

## 1. Introduction

Investment plays a crucial role in fostering economic growth while enhancing personal financial well-being. It enables individuals and institutions to grow their wealth over time, build financial security, and contribute to the overall prosperity of society. One of the primary advantages of investing is the ability to generate passive income. By investing in assets such as stocks, bonds, or real estate, individuals can earn dividends, interest, or rental income, supplementing their primary sources of income. This passive income can be reinvested to further increase wealth or used to fund their lifestyle, providing financial stability and independence [1]. Moreover, investing plays a crucial role in the economy by channeling funds toward productive activities, fostering innovation, creating jobs, and ultimately driving economic growth [2].

In recent years, the investment environment has expanded significantly, offering a wider range of market opportunities for investors [3]. Among these emerging markets, the cryptocurrency market has garnered significant attention and popularity, driven by its unique characteristics and potential for high returns [4]. Cryptocurrency offers several key benefits that make it an attractive investment option. First, it provides opportunities for diversification, allowing investors to reduce their exposure to traditional assets such as stocks and bonds [5]. Cryptocurrencies are also known for their high liquidity, enabling investors to buy and sell quickly at competitive prices. Additionally, the decentralized nature of cryptocurrencies offers greater transparency and security compared to traditional financial systems, as blockchain technology ensures that transactions are recorded in a secure, immutable ledger [6]. Moreover, cryptocurrencies have the potential for high growth due to their evolving use cases, including decentralized finance, digital assets, and smart contracts, making them appealing to risk-tolerant investors seeking substantial returns [7,8]. However, while this market presents significant investment opportunities, it also carries substantial risks due to its inherent volatility, regulatory uncertainties, and market dynamics [9,10]. Price fluctuations in the cryptocurrency market can be sudden and extreme, posing challenges even to experienced investors [11]. Therefore, implementing a well-structured investment strategy is essential for managing risks, preserving capital, and maximizing long-term returns. A wide range of investment strategies has been developed, each designed to align with specific risk tolerances and financial objectives. Among these approaches, portfolio optimization has emerged as one of the most effective and widely applied methods in modern investment management.

Portfolio optimization is a dynamic and evolving topic in the fields of finance and investment, playing an important role in modern asset management [12,13]. The concept was first introduced by Harry Markowitz [14] in 1952 through his groundbreaking work on Modern Portfolio Theory (MPT), which laid the foundation for systematic investment strategies. A portfolio, in the context of investment, refers to a collection of financial assets such as stocks, bonds, commodities, real estate, or cryptocurrencies held by an individual or institution. The primary goal of building a portfolio is to balance risk and return by diversifying investments across different asset classes. Diversification reduces the overall risk of the portfolio because the performance of various assets may not be perfectly correlated; when some assets decline in value, others may perform well, offsetting potential losses. Markowitz’s portfolio theory introduced the concept of efficient portfolios, which offer the highest expected return for a given level of risk or the lowest risk for a desired level of return. His mean-variance optimization framework uses expected returns, variances, and covariances of asset returns to determine optimal asset allocation. This approach remains a cornerstone of investment management, guiding both individual and institutional investors in constructing portfolios that align with their financial goals and risk tolerance. Portfolio optimization has attracted significant attention from both investors and researchers, driving continuous advancements in investment management techniques. As the financial markets evolved, the need for more robust risk management tools became evident, prompting researchers to develop a variety of risk measures tailored to different investment environments. Among these measures, Value at Risk (VaR) [15] and Conditional Value at Risk (CVaR) [16,17] have gained considerable prominence [18]. Both belong to the class of downside risk measures, focusing on potential losses rather than overall variability. VaR estimates the maximum expected loss over a specific time frame at a given confidence level, while CVaR goes a step further by assessing the average loss beyond the VaR threshold, providing a more comprehensive view of extreme risks [19,20]. Due to the highly volatile and unpredictable nature of the cryptocurrency market, downside risk measures like VaR and CVaR have better applicability in this context. They enable investors to evaluate and manage extreme losses, making them valuable tools for constructing more resilient and risk-aware investment portfolios in the crypto market.

However, despite advances in financial modeling, uncertainty remains a fundamental characteristic of capital markets, as much of the information used in investment decision-making is inherently uncertain, imprecise, or incomplete [21]. To tackle the challenges posed by this uncertainty, researchers have explored alternative approaches such as fuzzy set theory, first introduced by Zadeh [22]. Building on this concept, Liu [23] proposed credibility theory, which was further expanded in subsequent works [24]. Credibility theory offers several advantages in addressing uncertainty within financial markets. Unlike traditional probabilistic models that rely on precise statistical distributions, credibility theory provides a flexible framework for handling imprecise and incomplete data. It combines elements of probability and possibility theory, making it well-suited for environments characterized by ambiguity and vagueness. One key benefit of credibility theory is its ability to model uncertain asset returns using a more realistic representation of market conditions. This approach accounts for both optimistic and pessimistic scenarios, enabling more comprehensive risk assessments. Additionally, credibility measures are computationally efficient and can be easily integrated into portfolio optimization models, enhancing their practical applicability. Credibility theory has been applied in several portfolio optimization studies, demonstrating its effectiveness in managing uncertainty and enhancing investment strategies. However, its application in cryptocurrency portfolio optimization remains relatively underexplored. Given the unique volatility and uncertainty of the crypto market, further investigation is needed to harness the potential of this theory in this emerging asset class.

To address this research gap, this paper integrates credibility theory with the CVaR framework, leveraging their combined strengths in modeling downside risk and managing uncertainty. Additionally, practical constraints commonly encountered in real-world investment scenarios are considered, ensuring that the proposed framework is both theoretically sound and practically applicable in the dynamic cryptocurrency market. However, relying solely on portfolio optimization models may not be sufficient to achieve optimal investment outcomes. While these models offer valuable frameworks for asset allocation, they often overlook the critical step of selecting high-quality assets. A thorough pre-selection process is essential to identify assets with strong fundamentals, growth potential, and resilience to market fluctuations. By focusing on high-potential assets before applying portfolio optimization models, investors can enhance the effectiveness of their strategies. This ensures that the portfolio includes assets well-positioned for growth while minimizing potential risks. In the cryptocurrency market, pre-selection is particularly important due to the presence of numerous assets with limited investment value. Various methodologies have been proposed for asset pre-selection, including Data Envelopment Analysis (DEA), machine learning, and deep learning techniques. Among these, Multi-Attribute Decision-Making (MADM) methods have proven effective because they can evaluate assets based on multiple criteria, which is crucial for investors making informed decisions. However, a notable challenge with MADM methods is that different methods can produce varying results, creating uncertainty about which approach provides the most reliable outcome. To address this issue, this paper proposes a novel pre-selection framework based on MADM methods that delivers robust and consistent results. To the best of our knowledge, no existing study has applied a systematic pre-selection process in the context of cryptocurrency portfolio optimization. Therefore, this paper first introduces a comprehensive set of cryptocurrency performance criteria and utilizes the proposed pre-selection method based on these criteria. By integrating these components, this paper presents a two-stage framework for enhancing cryptocurrency portfolio performance. In the first stage, after defining relevant cryptocurrency performance criteria, we apply the novel pre-selection method. In the second stage, we implement a credibilistic CVaR model with practical investment constraints, creating a comprehensive and effective investment strategy for the cryptocurrency market.

The remainder of this paper is organized as follows: Section 2 provides a comprehensive and systematic literature review in four subsections, examining prior research on (I) cryptocurrency portfolio optimization, (II) portfolio optimization with asset pre-selection, (III) the application of credibility theory in portfolio optimization, and (IV) the identified research gap. Section 3 outlines the research methodology, beginning with the introduction of key concepts and definitions related to each stage of the proposed framework. This is followed by introducing alternatives and defining cryptocurrency performance criteria essential for the asset pre-selection process in Stage 1. Finally, the proposed optimization model for Stage 2, based on the credibilistic CVaR approach with practical investment constraints, is developed in detail. Section 4 presents the empirical results and computational analysis of the proposed two-stage framework, applying real-world cryptocurrency market data to evaluate its performance. Section 5 provides an in-depth discussion, critically analyzing the theoretical foundations and empirical outcomes of the proposed framework. Section 6 concludes the study by summarizing key findings and suggesting potential directions for future research.

## 2. Literature review

This section provides a comprehensive review of the relevant literature, divided into four subsections to cover all key aspects of the research. The first subsection reviews existing studies on cryptocurrency portfolio optimization, highlighting various methods and frameworks used in this emerging market. The second subsection focuses on portfolio optimization with asset pre-selection, emphasizing techniques employed to enhance investment outcomes through prior asset filtering. The third subsection explores the application of credibility theory in portfolio optimization, discussing its role in managing uncertainty and improving risk assessment. Finally, the fourth subsection identifies the research gap, outlining unexplored areas and justifying the need for the proposed two-stage framework.

### 2.1. Cryptocurrency portfolio optimization

Cryptocurrency portfolio optimization has become a prominent area of research, fueled by the inherent complexity and volatility of digital asset markets. As the cryptocurrency sector continues to expand, researchers and financial analysts have concentrated on crafting innovative models and strategies to effectively balance risk and maximize returns in this dynamic investment landscape. This literature review examines the wide range of methodologies and techniques applied to cryptocurrency portfolio optimization, highlighting significant findings and advancements that contribute to shaping the development of this fast-evolving domain.

James and Menzies [25] investigated whether the cryptocurrency market exhibits mathematical properties comparable to those of the equity market. Departing from traditional portfolio theory, which is grounded in the financial behavior of equity securities, their research focused on the purchasing patterns of retail cryptocurrency investors. The study emphasized collective market dynamics and portfolio diversification within the cryptocurrency domain, exploring the applicability of equity market findings to digital assets. Bowala and Singh [26] developed a data-driven risk forecasting approach tailored to cryptocurrency portfolios, addressing the skewness and kurtosis of returns. Critiquing traditional risk measures for their normality assumptions, they utilized high-frequency data to better capture volatility dynamics. Results showed superior performance in optimizing cryptocurrency portfolios, providing a robust framework for risk management in this volatile asset class. Sahu et al. [27] compared portfolio optimization methods and short-term strategies for the cryptocurrency market using high-frequency data from the top ten cryptocurrencies by market capitalization. The study evaluated Sharpe ratio maximization and kurtosis minimization to balance returns and risks, offering insights into optimizing portfolios in dynamic market conditions. Chen [28] examined the relevance and effectiveness of modern portfolio theory (MPT) in portfolios that include cryptocurrencies. The study assessed whether traditional MPT principles, such as diversification and risk–return optimization, remain applicable in the context of the high volatility and unique risk-return profiles characteristic of cryptocurrencies. Jeleskovic et al. [29] explored the potential benefits of integrating cryptocurrencies into traditional investment portfolios. Using a GARCH-Copula model within the Markowitz framework, the study evaluated whether such integration enhances portfolio performance, particularly by improving the Sharpe ratio and overall portfolio stability, offering new perspectives on optimizing mixed-asset portfolios. Kim et al. [30] examined the risk–return profiles of traditional, cryptocurrency, and hybrid portfolios, focusing on the impact of cryptocurrency integration into global portfolios. Using ensemble methods and tracing strategies, the study analyzed allocation ratios of 1%, 3%, and 5% across optimization techniques, including minimum variance, maximum diversification, equal risk contribution, and hierarchical risk parity. Results highlighted the influence of allocation levels on returns, volatility, Sharpe ratios, and maximum drawdowns, providing critical insights for optimizing portfolios with cryptocurrency exposure. Hrytsiuk et al. [31] introduced a modified Markowitz model for cryptocurrency portfolios, substituting the variance-based risk metric with VaR. By incorporating the Cauchy distribution characteristic of cryptocurrency returns, this approach addressed the standard model’s reliance on normality assumptions. The findings highlighted improved risk assessment and portfolio robustness, effectively capturing the heavy tails and extreme risks of volatile digital asset markets. Brauneis and Mestel [32] applied the Markowitz mean-variance framework to assess diversification benefits in cryptocurrency portfolios using daily data from the top 500 cryptocurrencies over three years. The study compared naïve diversification with optimization strategies targeting maximum return and minimum variance. Results demonstrated that diversified portfolios significantly reduced risk and outperformed single-asset investments, such as holding only Bitcoin, in overall performance. Ma et al. [33] used data from 2015 to 2019 to analyze the impact of integrating Bitcoin (BTC), Ethereum (ETH), Ripple (XRP), Bitcoin Cash (BTC), and Litecoin (LTC) into traditional portfolios. The study evaluated diversification effects and risk-return improvements across asset classes using various optimization techniques, emphasizing Ethereum and Bitcoin’s superior diversification benefits. Mba et al. [34] proposed two advanced cryptocurrency portfolio optimization models, GARCH-DE and GARCH-DE-t-copula, comparing them to the traditional Differential Evolution (DE) model. These models, tested in single- and multi-period frameworks, addressed complex dependency structures and extreme risks in cryptocurrency markets. The GARCH-DE-t-copula model, incorporating a t-copula, effectively captured tail dependencies and volatility clustering, demonstrating superior risk management and return optimization under market volatility. Aljinović et al. [35] introduced a multicriteria portfolio optimization approach leveraging the PROMETHEE II method, expanding beyond traditional return and risk measures. Their model incorporated diverse criteria, such as market capitalization, trading volume, VaR, CVaR, and the overall attractiveness of cryptocurrencies. Using data from January 2017 to February 2020, they demonstrated that their multicriteria approach achieved superior out-of-sample portfolio performance compared to conventional optimization models across various risk and return metrics. Maghsoodi [36] proposed a hybrid decision support system for cryptocurrency portfolio management, integrating time series forecasting via the Prophet Forecasting Model (PFM) with the enhanced CLUS-MCDA II algorithm. This system utilized advanced clustering methods, such as DBSCAN, alongside multicriteria decision analysis techniques like VIKOR and MULTIMOORA, to optimize allocation across more than 70 cryptocurrencies. Their model provided investors with a robust and informed tool to navigate the highly dynamic cryptocurrency market. Mba and Mwambi [37] developed the Markov-switching COGARCH-R-vine (MSCOGARCH) model to optimize cryptocurrency portfolios by addressing structural breaks, heavy tails, and volatility clustering. Compared to a single-regime COGARCH model, the MSCOGARCH model demonstrated superior risk estimation and portfolio optimization by accommodating regime changes in volatility. This approach offered enhanced flexibility for managing portfolios in the volatile cryptocurrency market. Ali et al. [19] explored the diversification benefits of green cryptocurrencies in the context of global portfolio optimization, introducing a novel four-step process to identify cryptocurrencies that are more energy-efficient than others. This study highlights the growing concern over the environmental impact of cryptocurrencies, particularly energy-intensive mining practices. By focusing on energy-efficient cryptocurrencies such as Cardano (ADA), Tezos (XTZ), and Stellar (XLM), the authors demonstrate how these green cryptocurrencies can serve as effective diversifiers for portfolios, offering benefits comparable to or even superior to their non-green counterparts like Bitcoin (BTC) and Ethereum (ETH). The research employed a variety of advanced econometric models, including dynamic conditional correlation (DCC-GARCH) and four-moment modified VaR, to evaluate the downside risk and expected shortfall of portfolios containing both green and non-green cryptocurrencies. In a recent study, Ghanbari et al. [38] proposed a robust framework for cryptocurrency portfolio optimization, leveraging the credibilistic CVaR criterion to address the distinct characteristics of digital asset markets. Recognizing the high volatility, frequent price swings, and the inherent uncertainty of cryptocurrencies, their approach models asset returns using fuzzy logic, enabling greater accuracy in risk assessment. Their proposed framework accounts for the unpredictable behavior of the crypto market, including extreme tail risks, offering a tailored solution for managing digital asset portfolios. This advancement underscores the potential of credibility-based models in navigating the complexities of cryptocurrency investment.

### 2.2. Portfolio optimization with asset preselection

Pre-selection plays a vital role in portfolio management by identifying and selecting assets prior to the portfolio optimization process. This step is particularly critical in volatile markets such as cryptocurrencies, where asset selection significantly affects portfolio performance. Pre-selection involves various methodologies aimed at filtering and selecting high-potential assets. This literature review explores the significance of pre-selection in portfolio optimization, emphasizing key developments and notable findings that shape this rapidly evolving field.

Lozza et al. [39] conducted an ex-post analysis of asset preselection frameworks, employing the joint Markovian dynamics of asset returns within stochastic market boundaries. Examining approximately 10,000 equities across 14 international markets, their findings substantiated the superior efficacy of Markovian-based methodologies over traditional Sharpe ratio optimization, emphasizing the strategic significance of probabilistic state transitions in enhancing portfolio efficiency amidst intricate market dynamics. Huang [40] developed an advanced stock selection paradigm integrating Support Vector Regression (SVR) for performance forecasting and Genetic Algorithms (GA) for parameter optimization and feature refinement. Equally weighted portfolios, derived from performance-ranked stocks, demonstrated empirically superior returns, affirming the model’s efficacy over conventional benchmarks in optimizing investment outcomes. Nguyen [41] introduced a sophisticated risk-measurement framework for large-scale datasets, integrating a stock preselection mechanism to exclude low-diversification stocks pre-optimization. By leveraging performance metrics such as the Sharpe ratio, Stutzer index, and Omega measure, the methodology enhanced portfolio construction. Empirical findings confirmed that preselection markedly improved portfolio performance and diversification, addressing critical challenges in large-scale optimization. Rather et al. [42] introduced a robust hybrid model for stock return prediction, integrating linear models, specifically the Autoregressive Moving Average and Exponential Smoothing techniques, with the nonlinear capabilities of a Recurrent Neural Network (RNN). By combining these methods, the framework leveraged their complementary strengths, while GA optimized the model’s weight distribution, ensuring balanced contributions. Experimental results demonstrated the hybrid model’s significant advantage over standalone RNNs, achieving superior predictive accuracy. Le Caillec et al. [43] proposed a stock selection model integrating behavioral uncertainty and probabilistic techniques, utilizing Cumulative Return and multiple Technical Indicators for preselection. Empirical analysis affirmed its efficacy in enhancing portfolio performance, addressing traditional strategy limitations by incorporating both technical analysis and behavioral dynamics. Fischer and Krauss [44] utilized a LSTM neural network to predict S&P 500 stock movements (1992–2015), outperforming models without memory functions (e.g., RF, DNN, LR). LSTM-based portfolios consistently exceeded alternatives, affirming the superiority of memory-enhanced architectures in financial time series analysis. Alizadeh et al. [45] developed a portfolio optimization model combining an adaptive neural fuzzy inference system for return prediction with a variance index for risk assessment. The model outperformed traditional Mean-Variance, neural network, and Sugeno–Yasukawa approaches, demonstrating the efficacy of integrating AI techniques with modern portfolio optimization for enhanced investment performance. Paiva et al. [46] introduced a unified decision-making model for day-trading in stock market investments, combining Support Vector Machines (SVM) with the MV framework for portfolio selection. The model was benchmarked against two alternatives: SVM + 1/N and Random+MV. Experimental results using assets from the Ibovespa stock market demonstrated that the proposed model delivered superior performance, highlighting its effectiveness in day-trading scenarios. Wang et al. [47] proposed a portfolio construction approach combining LSTM networks with the MV model. The LSTM network was employed to identify stock price patterns using various technical indicators, including the Relative Strength Index (RSI), Momentum Index (MOM), and True Range (TR). The MV model then optimized portfolios composed of five to ten assets. Comparative experiments revealed that the LSTM+MV method consistently outperformed other machine learning and MV-based models, particularly when portfolios included ten stocks. Ta et al. [48] constructed a portfolio using a LSTM neural network alongside three portfolio optimization techniques: the equal weight method, Monte Carlo simulation, and the Mean-Variance (MV) model. For comparison, linear regression and SVM were also applied in the stock selection process. Test results demonstrated that the LSTM neural network surpassed linear regression and SVM in prediction accuracy, and the portfolios it generated outperformed those built using the alternative methods. Chen et al. [49] proposed an innovative portfolio construction method integrating eXtreme Gradient Boosting (XGBoost) with an improved firefly algorithm (IFA) for stock price prediction. The Mean-Variance (MV) model was subsequently used to select and optimize portfolios containing varying numbers of stocks, focusing on those with higher predicted returns. Empirical evaluations revealed that the proposed hybrid model outperformed benchmark models, with its effectiveness being particularly notable when the portfolio consisted of seven stocks.

### 2.3. Portfolio optimization using credibility theory

Credibilistic portfolio optimization has gained significant attention in research due to its effectiveness in addressing uncertainty and ambiguity common in financial markets. Grounded in credibility theory and supported by fuzzy logic, this approach provides a robust framework for optimizing portfolios under conditions of high market volatility. Its capacity to incorporate expert opinions and subjective assessments into the investment decision-making process enhances its relevance in complex financial environments. This literature review explores the application of credibility theory in portfolio optimization, highlighting key methodologies, notable advancements, and empirical findings that underscore its value and potential in contemporary portfolio management.

Liu et al. [50] developed a credibilistic CVaR-based portfolio optimization model, enhancing traditional mean-variance frameworks by integrating CVaR of fuzzy variables to distinguish downside risks from upside potential. Solved via deterministic mixed-integer programming, the model ensures computational efficiency and refined risk management in portfolio optimization. Mohebbi and Najafi [51] proposed a multi-period fuzzy portfolio optimization model integrating credibility theory and scenario tree analysis to address market uncertainty. Using a bi-objective VaR framework, the model optimizes portfolios while incorporating transaction costs, risk-free investments, and practical constraints such as cardinality, thresholds, classes, and liquidity. Solved via interactive dynamic programming, the model combines fuzzy set theory with scenario analysis, offering a robust, adaptive tool for balanced portfolio optimization under real-world conditions. Deng et al. [52] developed a fuzzy mean-entropy portfolio optimization model leveraging credibility theory to enhance risk measurement and portfolio selection under uncertainty. The model integrates entropy as a risk measure, arguing its superiority over variance, especially in the context of fuzzy financial markets. It also incorporates transaction costs, addressing practical considerations often overlooked in traditional models. A sensitivity analysis was performed to assess the influence of parameter variations on the optimal portfolio configuration. The proposed approach aims to equip investors with a reliable and stable tool for portfolio optimization in uncertain and transaction-cost-sensitive financial environments. Liu et al. [53] proposed a multi-period portfolio optimization model designed to incorporate bankruptcy control within a fuzzy economic framework using credibility theory. The model seeks to maximize terminal wealth while simultaneously minimizing cumulative risk and uncertainty throughout the investment horizon. To enhance decision-making, it integrates affine recourse, addressing the impact of historical prediction biases on current portfolio adjustments. The authors also developed a hybrid particle swarm optimization algorithm to solve the model efficiently, providing a practical and robust tool for investors to manage risk and prevent bankruptcy in complex multi-period investment scenarios. Gupta et al. [54] introduced a multi-period portfolio optimization model utilizing coherent fuzzy numbers within a credibilistic framework to enhance decision-making under uncertainty. The model allows for greater flexibility in defining investor risk tolerance by incorporating mean absolute semi-deviation and CVaR as risk measures. It also addresses practical constraints, including cardinality, skewness, and transaction costs, to create realistic and adaptable investment strategies across multiple time horizons. The effectiveness of the proposed model was demonstrated through real-world case studies involving assets from the National Stock Exchange of India and major U.S. stock indices, noting its practical applicability and robustness in diverse market conditions. Mehlawat et al. [55] proposed a multiobjective portfolio optimization model employing coherent fuzzy numbers within a credibilistic framework. This model innovatively integrates investor attitudes—pessimistic, optimistic, or neutral—toward financial markets through a novel credibility function. It replaces variance with mean-absolute semi-deviation for a more realistic risk measure and incorporates skewness to account for the asymmetry of returns. Numerical examples and a genetic algorithm-based solution method highlight the model’s ability to capture investor preferences and handle market uncertainties effectively, offering enhanced flexibility and precision compared to traditional approaches. García et al. [56] extended the traditional mean-semivariance portfolio selection model by developing a multiobjective credibilistic framework that includes the Price-to-Earnings Ratio (PER) as an additional performance criterion. Using L-R power fuzzy numbers to represent uncertainty in asset returns and PER, the model addresses limitations of the classical mean-variance framework while integrating real-world constraints such as budget, bounds, and cardinality. Empirical tests on stocks from the Latin American Integrated Market demonstrated the model’s ability to generate a well-diversified set of efficient portfolios tailored to multiple objectives. In another study, García et al. [57] introduced an advanced multiobjective portfolio optimization model extending the stochastic mean-variance approach by incorporating fuzzy multiobjective criteria. Using trapezoidal fuzzy numbers, the model balances objectives related to return, risk, and liquidity, providing a robust framework for portfolio selection under uncertain and dynamic market conditions. Recently, Ghanbari et al. [38] extended the theoretical application of credibility theory in cryptocurrency portfolio optimization by incorporating trapezoidal fuzzy variables and the credibilistic CVaR framework to effectively model and quantify extreme tail risks. The inclusion of cardinality and allocation constraints further enhanced the model’s robustness, offering a comprehensive approach to managing the stochastic complexities of digital asset markets. This work represents a significant contribution to the field of quantitative financial optimization, setting a valuable benchmark for future research in cryptocurrency investment strategies.

### 2.4. Research gap

After conducting a comprehensive literature review from various perspectives relevant to this study, several critical research gaps have been identified that this paper aims to address:

1 *Lack of asset preselection in cryptocurrency portfolio optimization*

To the best of our knowledge, no existing study on cryptocurrency portfolio optimization incorporates an asset preselection stage, despite its crucial importance in such a highly volatile and diverse market. Given the wide range of cryptocurrencies with varying investment potentials, preselection is essential for filtering out low-quality assets. This paper addresses this gap by proposing a two-stage investment framework specifically designed for the cryptocurrency market. The first stage focuses on asset preselection, while the second stage involves portfolio optimization.

2 *Absence of cryptocurrency performance criteria*

Due to the lack of studies involving asset preselection in cryptocurrency portfolio optimization, there is also no established set of performance criteria tailored for evaluating cryptocurrencies. This gap leaves investors without clear guidelines for assessing asset quality before portfolio construction. To address this gap, this paper proposes a comprehensive set of cryptocurrency performance criteria that reflect fundamental, technical, and market-driven factors relevant to digital asset evaluation.

3 *Limitations of existing preselection methods*

Although various methodologies for asset preselection exist, MADM methods have emerged as one of the more effective approaches due to their ability to consider multiple evaluation criteria. However, a key limitation is that different MADM methods often produce varying results, causing confusion for investors when selecting assets. To address this issue, this paper proposes a novel preselection strategy based on MADM methods that generates robust and reliable results, reducing ambiguity in investment decision-making.

4 *Neglect of market uncertainty in cryptocurrency portfolio optimization*

Uncertainty is an inherent characteristic of the cryptocurrency market due to its extreme volatility, limited historical data, and unpredictable market dynamics. Despite its importance, uncertainty has been insufficiently addressed in existing cryptocurrency portfolio optimization models. To fill this gap, this paper applies a credibilistic CVaR model, which effectively accounts for uncertainty by incorporating fuzzy logic principles, providing a more realistic and adaptive risk management approach. This model also considers practical constraints, enhancing its applicability and relevance in real-world investment scenarios.

## 3. Research methodology

**Fig 1:**
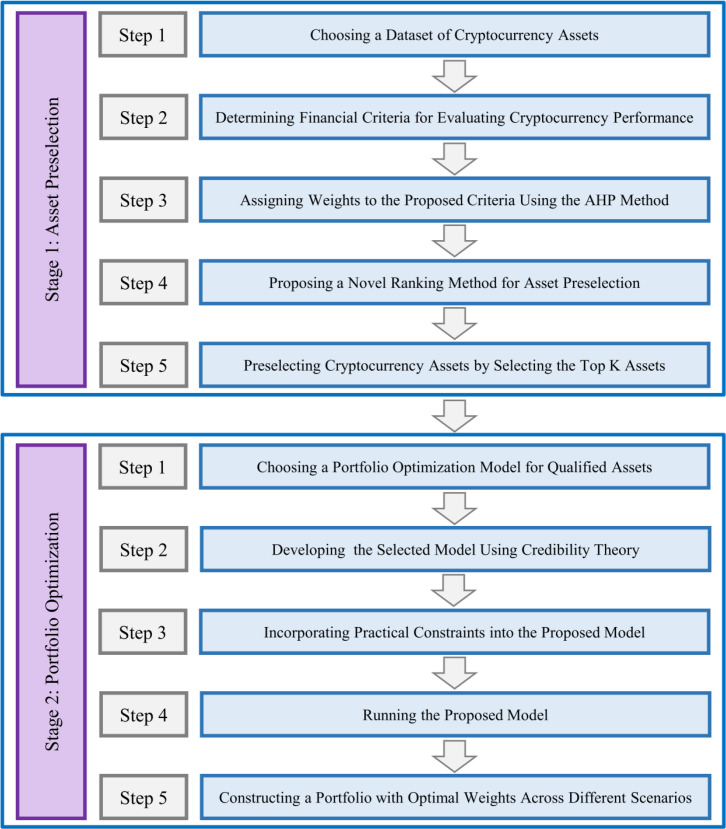
The methodology of proposed two-stage framework for enhancing cryptocurrency portfolio performance.

This paper presents a two-stage framework for enhancing cryptocurrency portfolio performance, designed to address portfolio construction challenges in volatile and uncertain cryptocurrency markets. The framework consists of two stages: In stage 1, we perform pre-selection of high-potential assets by a novel asset preselection approach, and in stage 2, we optimize the selected assets using a credibilistic CVaR approach (see Fig 1).

This section outlines the research methodology in detail and is divided into two subsections. Section 3.1, introduces the pre-selection process of Stage 1, and Section 3.2, provides a detailed description of the optimization process in Stage 2.

### 3.1. Stage 1 - Asset preselection

In Stage 1, we focus on the pre-selection of high-potential assets using a novel approach grounded in MADM methods. A notable challenge with MADM techniques is that different methods can yield varying results, leading to uncertainty about which approach provides the most reliable outcome. To address this issue, this paper proposes a robust pre-selection framework designed to provide consistent outcomes. This framework first calculates the results using a variety of methods, specifically employing 13 MADM techniques in this paper. Each of these methods offers unique strengths and perspectives in evaluating potential cryptocurrency assets. By utilizing multiple approaches, we aim to capture a comprehensive view of asset performance. Once the results are generated from these methods, they are systematically combined using the Copeland approach. This method evaluates and aggregates the outcomes, allowing us to rank the assets based on their overall performance across all selected MADM methods. By applying the Copeland approach, we enhance the reliability of the final selections, mitigating the inconsistencies that can arise from relying on a single MADM method. This dual-layered process not only strengthens our pre-selection framework but also ensures that the selected assets are those most likely to perform well in the dynamic cryptocurrency market.

In this section, we will first provide background knowledge on the 13 MADM methods and other approaches utilized in Stage 1 of our framework. Following that, we will describe the cryptocurrency dataset, which represents the alternatives in our analysis. In the third part, we will introduce the criteria for evaluating cryptocurrency performance. To the best of our knowledge, no existing study has implemented a systematic pre-selection process in the context of cryptocurrency portfolio optimization, resulting in a lack of benchmarks for these criteria. Therefore, we aim to establish a robust set of criteria for evaluating cryptocurrency performance, which can serve as a valuable benchmark for assessing cryptocurrencies during pre-selection or selection. Finally, we will employ the Analytic Hierarchy Process (AHP) method to determine the weight of each criterion, ensuring a systematic and rigorous evaluation framework.

#### 3.1.1. Background knowledge on MADM methods.

This paper employs a total of 13 MADM methods (see Table 1) to enhance the pre-selection process of cryptocurrency assets. These methods include MARCOS [58] (see Appendix 1 in S1 Appendix), CODAS [59] (Appendix 2 in S1 Appendix), CoCoSo [60] (Appendix 3 in S1 Appendix), EDAS [61] (Appendix 4 in S1 Appendix), WASPAS [62] (Appendix 5 in S1 Appendix), TOPSIS [63] (Appendix 6), MOORA [64] (Appendix 7), COPRAS [65] (Appendix 8), ARAS [66] (Appendix 9 in S1 Appendix), VIKOR [67] (Appendix 10 in S1 Appendix), MABAC [68] (Appendix 11 in S1 Appendix), MACBETH [69] (Appendix 12 in S1 Appendix), and TODIM [70] (Appendix 13 in S1 Appendix). Each method offers unique strengths and insights, collectively providing a robust framework for evaluating the potential of cryptocurrency assets in the context of our systematic pre-selection approach.

**Table 1:** Summary of employed MADM methods and corresponding references.

Method	Abbreviation	Reference
Measurement of Alternatives and Ranking according to COmpromise Solution	MARCOS	[58]
Combinative Distance-based Assessment	CODAS	[59]
COmbined COmpromise SOlution	CoCoSo	[60]
Evaluation based on Distance from Average Solution	EDAS	[61]
Weighted Aggregates Sum Product Assessment	WASPAS	[62]
Technique for Order of Preference by Similarity to Ideal Solution	TOPSIS	[63]
Multi-Objective Optimization on the basis of Ratio Analysis	MOORA	[64]
Complex PRoportional Assessment	COPRAS	[65]
Additive Ratio ASsessment	ARAS	[66]
VIseKriterijumska Optimizacija I Kompromisno Resenje	VIKOR	[67]
Multi-Attributive Border Approximation area Comparison	MABAC	[68]
Measuring Attractiveness by a Categorical Based Evaluation TecHnique	MACBETH	[69]
TOmada de Decisao Interativa e Multicriterio - Interactive and Multicriteria Decision Making	TODIM	[70]

Source: Authors’ own compilation

#### 3.1.2. Mean rank method.

The mean rank method is an effective decision-making technique used to evaluate multiple alternatives by assigning ranks based on specific criteria or performance metrics. Each alternative is assessed individually, receiving a rank relative to others—where the best-performing alternative is ranked 1, the second-best is ranked 2, and so on. After all ranks are assigned, the mean rank method calculates the average rank for each alternative. The alternative with the lowest average rank is then selected as the optimal choice, as a lower rank indicates better overall performance. This method is widely applicable in fields like finance, marketing, and operations management, offering a straightforward approach to streamline evaluations and minimize subjectivity in the ranking process.

#### 3.1.3. Borda count method.

The Borda count method [71] is a systematic approach for ranking alternatives based on their performance in pairwise comparisons. It begins by constructing an m×m matrix, where m represents the number of alternatives under consideration. Each entry in this square matrix is populated based on the number of wins each alternative achieves when compared to others. In this matrix, if an alternative in a given row has more wins than an alternative in a corresponding column, an “M” is placed in that entry. This notation signifies that the alternative in the row ranks higher than the one in the column across various decision-making scenarios. Conversely, if the number of wins in the row is equal to or less than that in the column, an “X” is recorded in that entry, indicating that the row’s alternative ranks equally or lower than the column’s alternative. After populating the matrix, the total number of wins for each alternative is calculated by summing the “M” entries in each row. This tally reflects the number of times each alternative has outperformed others in head-to-head comparisons. Finally, the alternatives are ranked based on the total number of wins, with those achieving a higher win count receiving a superior rank. This method not only provides a clear ranking but also emphasizes the comparative strengths of each alternative, making it a valuable tool in decision-making processes where multiple options need to be evaluated against one another.

#### 3.1.4. Copeland method.

Similar to the Borda count method, the Copeland method [72] also employs an m × m matrix to facilitate the ranking of alternatives based on their performance in pairwise comparisons. In this matrix, each entry is determined by the number of wins each alternative accumulates against others. Specifically, if an alternative in a given row has more wins than the alternative in a corresponding column, an “M” is placed in that entry. This indicates that the alternative in the row holds a higher rank in the context of various decision-making scenarios. Conversely, if the number of wins in the row is equal to or less than that in the column, an “X” is recorded, suggesting that the row’s alternative ranks equally or lower than the column’s alternative. Once the matrix is populated, the next step involves calculating the total number of wins for each alternative by summing the “M” entries in each row. This provides a clear indication of how many alternatives each option has outperformed. Additionally, the method also requires determining the number of losses for each alternative, which is done by summing the “M” entries in each column. The final ranking of the alternatives is derived from the difference between the total number of wins and losses for each option. Alternatives that exhibit a greater positive difference between their wins and losses are assigned higher ranks, reflecting their overall superiority in the comparison process. This approach not only allows for a nuanced ranking of alternatives but also emphasizes the relative strengths and weaknesses of each option, making the Copeland method a robust tool for decision-making in complex scenarios where multiple alternatives must be evaluated. By considering both wins and losses, the Copeland method provides a more balanced assessment compared to methods that focus solely on wins.

#### 3.1.5. Dataset description.

This section is dedicated to providing a comprehensive description of the dataset utilized in this study. The analysis focuses on 47 alternative cryptocurrencies, with data meticulously gathered from CoinGecko.com, a reputable platform known for its extensive cryptocurrency market data. The dataset encompasses daily price information spanning from December 1, 2023, to December 14, 2024. This timeframe allows for a thorough examination of price movements and trends in the rapidly evolving cryptocurrency market. From the daily price data, returns for each asset were calculated to assess their performance over the specified period. Descriptive statistics for the selected assets are summarized in Table 2, offering a clear view of key metrics such as mean, median, standard deviation, and other relevant statistical measures. This table serves as a crucial reference for understanding the characteristics of the assets under consideration.

**Table 2:** Summary of descriptive statistics for selected cryptocurrency assets.

Alt	Name	Coin/Token	Count	Mean	SD	Min	25%	50%	75%	Max	Variance
1	ETH	Ethereum	379	0.0023	0.0334	-0.1006	-0.0148	0.0022	0.0198	0.1905	0.0011
2	SOL	Solana	379	0.0045	0.0446	-0.1362	-0.0253	0.0001	0.0337	0.1442	0.0020
3	TRX	Tron	379	0.0037	0.0520	-0.2090	-0.0081	0.0016	0.0120	0.8888	0.0027
4	BNB	BSC (Binance)	379	0.0035	0.0304	-0.0852	-0.0131	0.0019	0.0173	0.1651	0.0009
5	BTC	Bitcoin	379	0.0030	0.0275	-0.0824	-0.0110	0.0017	0.0158	0.1227	0.0008
6	LINK	Chainlink	379	0.0029	0.0467	-0.1504	-0.0264	-0.0004	0.0280	0.3219	0.0022
7	ARB	Arbitrum	379	0.0013	0.0502	-0.1712	-0.0272	-0.0022	0.0263	0.2444	0.0025
8	SUI	Sui	379	0.0073	0.0624	-0.1656	-0.0319	0.0002	0.0314	0.3931	0.0039
9	AVAX	Avalanche	379	0.0073	0.0624	-0.1656	-0.0319	0.0002	0.0314	0.3931	0.0039
10	POL	Polygon	379	0.0004	0.0446	-0.1703	-0.0268	-0.0022	0.0245	0.1536	0.0020
11	CRV	Curve	379	0.0035	0.0579	-0.2035	-0.0276	-0.0010	0.0311	0.2730	0.0033
12	APT	Aptos	379	0.0032	0.0522	-0.1761	-0.0273	-0.0025	0.0279	0.2484	0.0027
13	OP	Optimism	379	0.0028	0.0590	-0.1647	-0.0344	-0.0015	0.0354	0.3937	0.0035
14	CORE	CORE	379	0.0067	0.0959	-0.2911	-0.0344	-0.0035	0.0259	0.8688	0.0092
15	UNI	Uniswap	379	0.0045	0.0597	-0.1410	-0.0259	0.0004	0.0270	0.5379	0.0036
16	MNT	Mantle	379	0.0032	0.0458	-0.1012	-0.0223	-0.0007	0.0208	0.3299	0.0021
17	CRO	Cronos	379	0.0032	0.0532	-0.1483	-0.0202	-0.0019	0.0201	0.6701	0.0028
18	ONDO	Ondo	330	0.0088	0.0046	0.0677	-0.0329	0.0003	0.0438	0.3836	0.1439
19	NUM	Numbers	379	0.0074	0.1118	-0.2281	-0.0408	-0.0093	0.0312	1.1497	0.0125
20	CAKE	Pancakeswap	379	0.0022	0.0469	-0.1965	-0.0228	0.0006	0.0234	0.2104	0.0022
21	MKR	Maker	379	0.0019	0.0459	-0.1455	-0.0235	-0.0021	0.0205	0.2367	0.0021
22	RUNE	Thorchain	379	0.0016	0.0572	-0.1824	-0.0352	-0.0017	0.0350	0.3148	0.0033
23	TON	TON	379	0.0035	0.0443	-0.1497	-0.0207	0.0002	0.0223	0.2294	0.0020
24	ADA	Cardano	379	0.0039	0.0449	-0.1588	-0.0212	0.0006	0.0248	0.2276	0.0020
25	GNO	Gnosis	379	0.0020	0.0418	-0.1232	-0.0224	0.0006	0.0241	0.2099	0.0017
26	AAVE	Aave	379	0.0048	0.0501	-0.1725	-0.0254	0.0002	0.0282	0.2823	0.0025
27	AR	Arweave	379	0.0051	0.0696	-0.2026	-0.0399	-0.0019	0.0403	0.5116	0.0048
28	DYDX	dYdX	379	0.0004	0.0541	-0.2204	-0.0305	-0.0017	0.0266	0.3138	0.0029
29	NEAR	Near	379	0.0052	0.0605	-0.1712	-0.0315	-0.0014	0.0379	0.3681	0.0037
30	1INCH	1inch	379	0.0023	0.0502	-0.2352	-0.0281	0.0024	0.0295	0.2447	0.0025
31	ROSE	Oasis	379	0.0020	0.0513	-0.1951	-0.0316	-0.0040	0.0337	0.1863	0.0026
32	SEI	Sei	379	0.0046	0.0682	-0.1820	-0.0373	-0.0057	0.0375	0.2713	0.0047
33	ONE	Harmony	379	0.0041	0.0560	-0.1984	-0.0299	0.0020	0.0369	0.2582	0.0031
34	MANA	Decentraland	379	0.0023	0.0503	-0.1877	-0.0226	0.0021	0.0254	0.3839	0.0025
35	KAVA	Kava	379	0.0003	0.0436	-0.2042	-0.0222	0.0022	0.0256	0.1779	0.0019
36	RON	Ronin	379	0.0031	0.0504	-0.1811	-0.0287	0.0007	0.0318	0.1700	0.0025
37	VET	Vechain	379	0.0040	0.0516	-0.1661	-0.0256	-0.0001	0.0302	0.3717	0.0027
38	LTC	Litecoin	379	0.0022	0.0381	-0.1838	-0.0163	0.0020	0.0197	0.1863	0.0014
39	EOS	EOS	379	0.0023	0.0461	-0.2158	-0.0218	0.0024	0.0226	0.2390	0.0021
40	CELO	Celo	379	0.0028	0.0566	-0.1891	-0.0292	0.0004	0.0294	0.5029	0.0032
41	FTM	Fantom	379	0.0057	0.0605	-0.1893	-0.0365	0.0041	0.0433	0.2945	0.0037
42	EGLD	MultiversX	379	0.0012	0.0439	-0.1943	-0.0243	-0.0014	0.0276	0.1340	0.0019
43	STX	Stacks	379	0.0050	0.0618	-0.1726	-0.0329	-0.0007	0.0358	0.4108	0.0038
44	XMR	Monero	379	0.0013	0.0382	-0.3663	-0.0126	0.0024	0.0168	0.2379	0.0015
45	ATOM	Cosmos	379	0.0009	0.0426	-0.1692	-0.0229	-0.0017	0.0221	0.1887	0.0018
46	XTZ	Tezos	379	0.0027	0.0504	-0.1824	-0.0204	0.0001	0.0230	0.4876	0.0025
47	ALGO	Algorand	379	0.0044	0.0525	-0.1587	-0.0260	0.0019	0.0288	0.3759	0.0028

Source: Authors’ own compilation based on data from CoinGecko.com

It is important to highlight that the descriptive statistics for all assets were computed based on a dataset comprising 379 days of data. However, the asset ONDO presents a slight deviation, as it has only been available for 330 days (the asset was launched 330 days ago). This discrepancy is worth noting, as it may affect the reliability of the statistical analysis for ONDO compared to the other assets included in the study.

However, since the difference between 330 and 379 days is relatively small, we believe this deviation can be neglected for the purposes of this study. The difference of just 49 days is unlikely to significantly distort the overall trends or findings. Therefore, we think it is beneficial to consider ONDO as an option rather than removing it entirely, as its inclusion may offer valuable insights, particularly as the asset develops further and additional data becomes available. Removing it could limit the scope of our analysis, especially considering that many portfolio optimization studies may encounter similar data limitations with newly launched assets.

#### 3.1.6. Criteria for evaluating cryptocurrency performance.

This part is dedicated to the description of the criteria for evaluating cryptocurrency performance. In this paper, we introduce 11 comprehensive criteria for assessing crypto assets, as detailed in Table 3. This table not only presents the criteria themselves but also outlines the types of benefits and costs associated with each criterion, thereby providing a clear and structured framework for evaluation. To the best of our knowledge, no existing study has implemented a systematic pre-selection process specifically in the context of cryptocurrency portfolio optimization. This gap has resulted in a lack of established benchmarks for these criteria, making it challenging for researchers and practitioners to assess cryptocurrency assets effectively. To address this issue, we aim to establish a robust set of criteria that can serve as a valuable benchmark for evaluating cryptocurrencies during the pre-selection phase. Our criteria are designed to ensure that the assessment of cryptocurrency performance is both rigorous and comprehensive, addressing the unique challenges posed by this dynamic asset class. By developing these criteria, we hope to facilitate more informed decision-making in the rapidly evolving cryptocurrency market. This systematic approach not only enhances the reliability of our evaluations but also contributes to the broader field of cryptocurrency research by providing a structured methodology that others can replicate.

**Table 3:** Comprehensive criteria for evaluating cryptocurrency performance.

Criteria	Cost/Benefit	Description
Avg TPS	Benefit	The average number of transactions processed per second (TPS) on a blockchain network. This metric indicates the network’s speed and capacity to handle transactions.
Avg Active Addresses	Benefit	The average number of addresses that participate in network transactions within a specific time frame (in this case, daily). It counts addresses that have conducted at least one transaction on the network.
Max Drawdown From ATH	Cost	The maximum percentage decline in the value of an asset from its all-time high (ATH). This metric is considered a measure of cost and risk, demonstrating how much a project’s price can drop during bearish market conditions.
Avg TVL (Billion Dollars)	Benefit	The average total value locked (TVL) in a decentralized finance (DeFi) protocol. It indicates how much value (typically cryptocurrencies) is locked in smart contracts on a platform.
Avg Fee (Dollars)	Cost	The average transaction fee on a blockchain network, referring to the cost’s users incur to execute transactions. These fees are typically paid to miners or validators as rewards for processing and validating transactions. Transaction fees can vary depending on network congestion, transaction size, and user prioritization.
Avg Daily Volume (Billion Dollars)	Benefit	The average daily trading volume of a cryptocurrency. This metric includes buy and sell transactions across all trading platforms (both centralized and decentralized exchanges).
Avg M-Cap (Billion Dollars)	Benefit	The average total market value of a project in the cryptocurrency market. Market cap is calculated by multiplying the total circulating supply of tokens by the current price per token.
Total Revenue (Million Dollars)	Benefit	Refers to the total income generated by a blockchain or protocol through various activities, such as transaction fees, service fees, and other financial operations. This revenue is typically collected from users or participants within the ecosystem.
Circulating/Total Supply	Benefit	This ratio represents the proportion of tokens in circulation relative to the total token supply. It indicates what percentage of a cryptocurrency’s total supply is currently available and tradable in the market.
Full-Time Developers	Benefit	Refers to developers who work full-time on the development, improvement, and maintenance of a blockchain project. These individuals are part of the project’s technical team and consistently write, update, and test the project’s code. This metric reflects the strength of the development team.
Total Developers	Benefit	Refers to the total number of developers involved in the development of a blockchain project, whether full-time, part-time, or volunteer. This metric includes all contributors who have updated or participated in the project’s code repositories, such as those on GitHub.

Note: “Ave” = Average, “Max” = Maximum

Source: Authors’ own compilation

**Fig 2:**
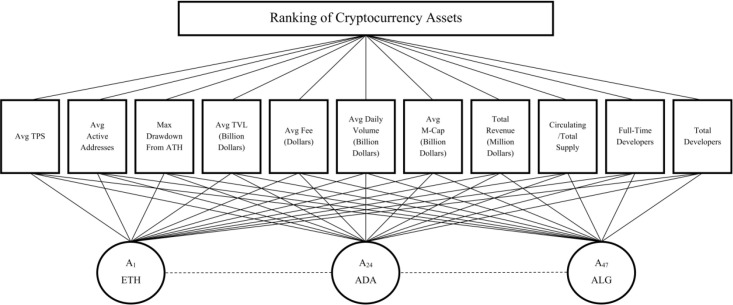
Schematic representation of the AHP model for weighting evaluating cryptocurrency criteria.

Next, we employed the Analytic Hierarchy Process (AHP) method [73] to determine the weight of each criterion involved in the evaluation of cryptocurrency performance. AHP is a structured decision-making tool that allows for the systematic comparison of multiple criteria by breaking down complex problems into a hierarchy of manageable components. This method facilitates a thorough assessment by enabling decision-makers to evaluate the relative importance of each criterion through pairwise comparisons. The structure of the AHP model is illustrated in Fig 2, which visually represents the hierarchical arrangement of the criteria. This figure outlines how the criteria are organized, showing the relationships and dependencies among them. At the top of the hierarchy is the overall goal, followed by the criteria that contribute to this goal.

The results of the AHP analysis are presented in Table 4, highlighting the weights assigned to each criterion based on pairwise comparisons.

**Table 4:** Weights assigned to each criterion based on AHP method.

Code	Criteria	Weight
C_1_	Avg TPS	11.10%
C_2_	Avg Active Addresses	9.30%
C_3_	Max Drawdown From ATH	3.30%
C_4_	Avg TVL (Billion Dollars)	17.80%
C_5_	Avg Fee (Dollars)	10.90%
C_6_	Avg Daily Volume (Billion Dollars)	6.40%
C_7_	Avg M-Cap (Billion Dollars)	9.00%
C_8_	Total Revenue (Million Dollars)	18.10%
C_9_	Circulating/Total Supply	4.30%
C_10_	Full-Time Developers	2.50%
C_11_	Total Developers	7.20%

Source: Authors’ own computation from AHP method

In total, 55 comparisons were made during the AHP process, resulting in a Consistency Ratio (CR) of 1.5%, indicating a high level of consistency in the judgments made. The principal eigenvalue calculated from the AHP model was found to be 11.221, further confirming the reliability of the derived weights. This systematic approach ensures that the evaluation of cryptocurrency assets is both rigorous and justified, enhancing the robustness of our framework.

### 3.2. Stage 2 - Portfolio optimization

After introducing the pre-selection process (Stage 1) in the previous section, this section focuses on the optimization process. Given that uncertainty is a fundamental characteristic of capital markets, this paper integrates credibility theory with the CVaR framework, effectively leveraging their combined strengths to model downside risk and manage uncertainty during the optimization process of Stage 2. Furthermore, practical constraints commonly encountered in real-world investment scenarios are taken into account, ensuring that the proposed framework is both theoretically robust and practically applicable within the dynamic cryptocurrency market. This integrated model is employed for constructing portfolios, facilitating more informed investment decisions.

In this section, we will first present key concepts and definitions related to fuzzy theory and credibility measures, providing a foundational understanding necessary for the subsequent discussion. Following this, we will describe the proposed model in detail, elucidating how it addresses the challenges of uncertainty and risk management in cryptocurrency portfolio optimization.

#### 3.2.1. *Some concepts and definitions in fuzzy theory and credibility measure.*

In this section, we will revisit several fundamental concepts and definitions that are crucial for understanding the subsequent content. We will specifically focus on three key areas: (I) Fuzzy set theory, (II) Fuzzy numbers, and (III) Credibility theory. Together, these elements form an integral part of the analytical framework that supports this study.

Definition 1. Fuzzy set theory

Classical set theory evaluates elements based on binary criteria, determining whether an element is either a member of a set or not. This black-and-white approach can be limiting, as it does not account for the complexities of real-world situations where membership may not be absolute. In contrast, fuzzy set theory offers a more sophisticated mathematical framework that allows for a nuanced evaluation of element membership within a set. In fuzzy set theory, this evaluation is represented through a membership function, which assigns each element a membership grade that ranges from zero to one. This functionality enables the model to effectively capture the vagueness and uncertainty inherent in various scenarios, allowing for a more flexible representation of reality.

Let X denote a universe of discourse, with its generic element represented as x. A fuzzy set A can be characterized as a collection of ordered pairs defined within the universe X, expressed mathematically as follows:


$$A=\left\{\left(x,\mu_{A}(x)|\;x\in X\right)\right\}$$ (1)

In this expression, $\mu_{A}(x)$ denotes the membership function, which quantifies the degree of membership of an element x∈X. The membership function is defined within the real interval [0,1]. A value of $\mu_{A}(x)=1$ indicates full membership, meaning that the element completely belongs to the fuzzy set A, while a value of $\mu_{A}(x)=0$ signifies no membership. Intermediate values represent varying degrees of membership, reflecting how strongly the element x belongs to the fuzzy set A. This flexibility to express partial membership is particularly useful in domains where uncertainty and ambiguity are prevalent, such as in decision-making processes, risk assessment, and various applications in economics, finance and engineering. By accommodating the complexities of real-world situations, fuzzy set theory enhances our ability to model and analyze data in a way that traditional set theory cannot.

Definition 2. Fuzzy Numbers

In practical applications, it is common for experts to express their assessments and judgments using fuzzy numbers. These fuzzy numbers are particularly useful in situations where uncertainty or imprecision is inherent in the data or the information being conveyed. Fuzzy numbers allow for a more flexible representation of values, enabling decision-makers to capture the nuances of their evaluations. Two specific forms of fuzzy numbers that are widely used are triangular and trapezoidal fuzzy numbers. These forms are defined by their unique characteristics as follows:

*Triangular fuzzy number* Let $\tilde{A}$ be defined as a triangular fuzzy number characterized by the parameters $\left(a_{1},\;a_{2},a_{3}\right)$, where $a_{1}$, $a_{2}$, and $a_{3}$ are real numbers satisfying $a_{1}\leq a_{2}\leq a_{3}$. The membership function $\mu_{A}(x)$ associated with $\tilde{A}$ is defined piecewise as follows:


$$\mu_{\tilde{A}}(x)=\left\{ \begin{array}{cc} \;\;\;\;\;\;\;\;\;\;0, & x\in \left(-\infty ,a_{1}\right)\\ \frac{x-a_{1}}{a_{2}-a_{1}}, & \;\;\;\;\;x\in \left[a_{1},a_{2}\right]\\ \frac{a_{3}-x}{a_{3}-a_{2}}, & \;\;\;\;\;x\in \left[a_{2},a_{3}\right]\\ \;\;\;\;\;\;\;\;\;\;0, & x\in \left(a_{3},+\infty \right) \end{array}\right.\;$$ (2)

**Fig 3:**
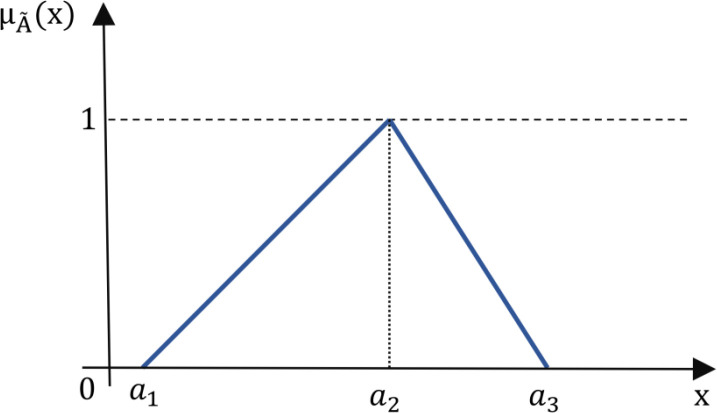
Visual representation of a triangular fuzzy number.

In this representation, the triangular fuzzy number $\tilde{A}$ achieves its maximum membership value of 1 at $x=a_{2}$. As x moves away from $a_{2}$ towards either $a_{1}$ or $a_{3}$, the membership values decrease linearly to 0, creating the characteristic triangular shape depicted in Fig 3.

*Trapezoid fuzzy number* Let $\tilde{A}$ be defined as a trapezoid fuzzy number characterized by the parameters $\left(a_{1},\;a_{2},a_{3},a_{3}\right)$, where $a_{1}$, $a_{2}$, $a_{3}$ and $a_{4}$ are real numbers satisfying $a_{1}\leq a_{2}\leq a_{3}\leq a_{4}$. The membership function $\mu_{A}(x)$ associated with $\tilde{A}$ is defined piecewise as follows:


$$\mu_{\tilde{A}}(x)=\left\{ \begin{array}{cc} \;\;\;\;\;\;\;\;\;0, & x\in \left(-\infty ,a_{1}\right)\\ \frac{x-a_{1}}{a_{2}-a_{1}}, & \;\;\;\;\;\;\;x\in \left[a_{1},a_{2}\right]\\ \;\;\;\;\;\;\;\;\;\;\;1, & \;\;\;\;\;\;\;x\in \left[a_{2},a_{3}\right]\\ \frac{a_{4}-x}{a_{4}-a_{3}}, & \;\;\;\;\;\;\;x\in \left[a_{3},a_{4}\right]\\ \;\;\;\;\;\;\;\;\;\;0, & x\in \left(a_{4},+\infty \right) \end{array}\;\right.\;$$ (3)

**Fig 4:**
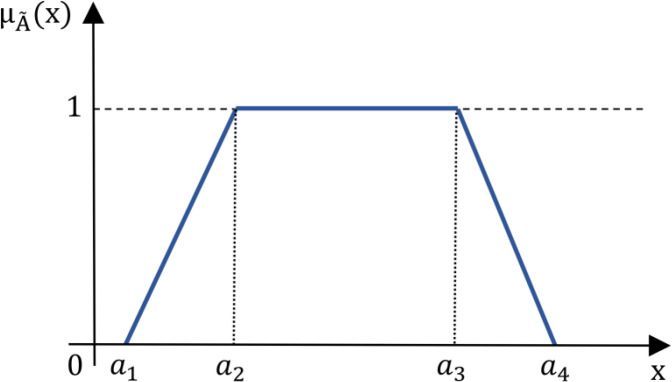
Visual representation of a trapezoid fuzzy number.

The membership function for a trapezoidal fuzzy number takes on a trapezoidal shape, where the fuzzy number $\tilde{A}$ maintains a constant maximum membership value of 1 over the interval ${[a}_{2},a_{3}]$. Outside this interval, the membership values decrease linearly to 0 as x approaches $a_{1}$ or $a_{4}$, resulting in the characteristic trapezoidal shape depicted in Fig 4.

Definition 3. Credibility theory

Credibility theory, originally introduced by Liu [23] and further developed in subsequent works [24], provides a robust mathematical framework for analyzing and modeling fuzzy phenomena. This theory is essential for the advancement of credibility fuzzy programming, which allows decision-makers to quantify and evaluate their level of confidence in meeting specific constraints amid uncertainty. Credibility theory offers a versatile approach to managing various types of fuzzy data, such as triangular and trapezoidal fuzzy numbers, which are commonly employed to represent uncertain information in decision-making processes. By incorporating these fuzzy numbers, the theory facilitates a more nuanced and realistic portrayal of uncertainty encountered in real-world situations. Liu [23,24] lays out the fundamental definitions and notations that form the basis of credibility theory, including the concept of the credibility measure. This measure is a crucial tool for assessing the likelihood or degree of confidence that a particular event or condition will occur within a fuzzy context. As highlighted by Liu and Liu [74], the calculation of the credibility measure is expressed as follows:


$$Cr\{\xi \in A\}=\frac{1}{2}(Pos\{\xi \in A\}+Nes\{\xi \in A\})$$ (4)

In this equation, Pos{ξ∈A} denotes the possibility measure of the event {ξ∈A}, while Nes{ξ∈A} represents the necessity measure of the same event. Both measures are fundamental concepts in fuzzy set theory:

- *Possibility Measure* (Pos): The possibility measure, denoted as Pos{ξ∈A}, evaluates the highest degree of membership of the variable ξ within the fuzzy set A. This measure reflects the most plausible extent to which the event {ξ∈A} can occur, capturing the potential for membership in a fuzzy context. It is mathematically defined as:

$$Pos\{\xi \in A\}=sup\;{\mu (x)}_{x\in A}$$ (5)

Here, $sup\;{\mu (x)}_{x\in A}$ signifies the supremum (or least upper bound) of the membership function μ(x) for all x within the set A. This measure essentially provides a value between 0 and 1, indicating the highest degree to which ξ can be considered a member of A.

- *Necessity Measure* (Nes): In contrast, the necessity measure Nes{ξ∈A} assesses the degree of certainty associated with the event {ξ∈A}. It is calculated as the complement of the highest degree of membership found in the complement set $A^{c}$, providing a measure of how definitive the membership is. This is expressed as:

$$Nes\{\xi \in A\}=1-\sup{\mu (x)}_{x\in A^{c}}$$ (6)

In this formulation, $\sup{\mu (x)}_{x\in A^{c}}$ represents the maximum degree of membership for any element outside the set A. By taking the complement, this measure effectively captures the certainty that ξ belongs to A.

Since $Pos\{\xi \in A\}=sup\;{\mu (x)}_{x\in A}$ and $Nes\{\xi \in A\}=1-\sup{\mu (x)}_{x\in A^{c}}$, the credibility measure can also be formulated as:


$$Cr\{\xi \in A\}=\frac{1}{2}(sup\;{\mu (x)}_{x\in A}+1-\sup{\mu (x)}_{x\in A^{c}})$$ (7)

This formulation illustrates that the credibility measure takes into account both the possibility and necessity of the event {ξ∈A}, offering a thorough evaluation of its likelihood within a fuzzy context.

When examining a specific fuzzy event characterized by {ξ≤r}, where r is a real number, the credibility measure is expressed as:


$$Cr\{\xi \leq r\}=\frac{1}{2}(sup\;{\mu (x)}_{x\leq r}+1-\sup{\mu (x)}_{x>r})$$ (8)

This equation allows for the assessment of the credibility that a fuzzy variable ξ will assume a value less than or equal to a specified real number r. It considers both the maximum membership value within the interval (−∞,r] and the complement membership value in the interval (r,+∞), providing a balanced perspective on the likelihood of the event.

Another key concept in credibility theory is the expected value of a fuzzy variable ξ, which represents the “average” outcome of ξ while accounting for its fuzzy characteristics. It is calculated using the following expression:


$$E[\xi ]=\int_{0}^{+\infty }Cr\left\{\xi \geq r\right\}dr-\int_{-\infty }^{0}Cr\left\{\xi \leq r\right\}dr$$ (9)

Equation (9) integrates the credibility measures across the entire real line, effectively determining the expected value of ξ by weighing contributions from both positive and negative ranges. The first integral evaluates the credibility of ξ being greater than or equal to r, while the second integral assesses the credibility of ξ being less than or equal to r.

The following sections of the paper will concentrate on applying the credibility measure to specific types of fuzzy variables, specifically triangular and trapezoidal fuzzy numbers.

*Credibility measure for triangular fuzzy numbers* Consider a fuzzy variable characterized by the triplet $\left(a_{1},a_{2},a_{3}\right)$ of crisp numbers, where $\left(a_{1}\leq a_{2}\leq a_{3}\right)$. Utilizing the general formula for credibilistic expected value (as presented in Equation (9)), we can derive the credibilistic expected value of the triangular fuzzy variable ξ as follows:


$$E[\xi ]=\frac{a_{1}+2a_{2}+a_{3}}{4}$$ (10)

This expression indicates that the expected value of the triangular fuzzy variable is a weighted average, giving more significance to the mode $a_{2}$ due to its central position within the triangular structure.

Next, we determine the credibility measure Cr{ξ≤r} for a triangular fuzzy number using the following equation:


$$Cr\{\xi \leq r\}=\left\{ \begin{array}{cc} \;\;\;\;\;\;\;\;\;\;\;\;\;\;\;\;\;0, & \;\;\;\;\;\;\;\;\;\;\;\;\;\text{\textasciitilde{}}r\leq a_{1}\\ \mathrm{\backslash vspace}1mm\frac{r-a_{1}}{2(a_{2}-a_{1})}, & \text{\textasciitilde{}}a_{1}\leq r\leq a_{2}\\ \mathrm{\backslash vspace}1mm\frac{a_{3}-2a_{2}+r}{2(a_{3}-a_{2})}, & \text{\textasciitilde{}}a_{2}\leq r\leq a_{3}\\ \;\;\;\;\;\;\;\;\;\;\;\;\;\;\;\;\;\;1, & \;\;\;\;\;\;\;\;\;\;\;\;\text{\textasciitilde{}}a_{3}\leq r \end{array}\right.\;$$ (11)

This measure captures the probability that the fuzzy variable ξ will take on a value less than or equal to a specific real number r. It incorporates different cases depending on the value of r relative to the parameters $a_{1},a_{2},\;$and $a_{3}$.

Similarly, the credibility measure Cr{ξ≥r} for a triangular fuzzy number can be expressed as:


$$Cr\{\xi \geq r\}=\left\{ \begin{array}{cc} \;\;\;\;\;\;\;\;\;\;\;\;\;\;\;\;\;\;\;1, & \text{\textasciitilde{}}\;\;\;\;\;\;\;\;\;\;\;\;\;r\leq a_{1}\\ \mathrm{\backslash vspace}1mm\frac{2a_{2}-a_{1}-r}{2(a_{2}-a_{1})}, & \text{\textasciitilde{}}a_{1}\leq r\leq a_{2}\\ \frac{a_{3}-r}{2(a_{3}-a_{2})}, & \text{\textasciitilde{}}a_{2}\leq r\leq a_{3}\\ \;\;\;\;\;\;\;\;\;\;\;\;\;\;\;\;0, & \text{\textasciitilde{}}\;\;\;\;\;\;\;\;\;\;\;\;\;a_{3}\leq r \end{array}\right.\;$$ (12)

This measure assesses the probability that the fuzzy variable ξ will assume a value greater than or equal to r. Like the previous measure, it accounts for different scenarios based on the position of r relative to the triangular parameters.

**Fig 5:**
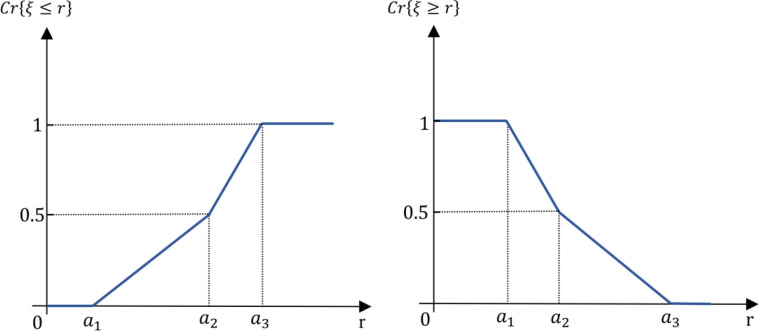
Credibility measures for a triangular fuzzy variable.

Fig 5 visually represents these credibility measures, illustrating the likelihood of various outcomes in the context of a triangular fuzzy variable. The graphical depiction aids in understanding how the credibility measures change with different values of r and highlights the inherent uncertainty associated with fuzzy variables.

*Credibility measure for trapezoidal fuzzy numbers* Consider a fuzzy variable defined by the quadruplet $\left(a_{1},a_{2},a_{3},a_{4}\right)$ of crisp numbers, where $\left(a_{1}\leq a_{2}\leq a_{3}\leq a_{4}\right)$. Using the general formula for the credibilistic expected value (as outlined in Equation (9)), we can define the expected value for a trapezoidal fuzzy variable ξ as follows:


$$E[\xi ]=\frac{a_{1}+a_{2}+a_{3}+a_{4}}{4}$$ (13)

This equation indicates that the expected value of the trapezoidal fuzzy variable is the arithmetic mean of its four parameters, reflecting the central tendency of the fuzzy number.

Next, we determine the credibility measure Cr{ξ≤r} for a trapezoidal fuzzy number, which is expressed as:


$$Cr\{\xi \leq r\}=\left\{ \begin{array}{cc} \;\;\;\;\;\;\;\;\;\;\;\;\;\;\;\;\;\;\;\;0, & \text{\textasciitilde{}}\;\;\;\;\;\;\;\;\;\;\;\;\;\;\;r\leq a_{1}\\ \mathrm{\backslash vspace}1mm\;\;\;\;\;\frac{r-a_{1}}{2(a_{2}-a_{1})}, & \text{\textasciitilde{}}a_{1}\leq r\leq a_{2}\\ \mathrm{\backslash vspace}1mm\;\;\;\;\;\;\;\;\;\;\;\;\;\;\;\;\;\;\;\;\frac{1}{2}, & \text{\textasciitilde{}}a_{2}\leq r\leq a_{3}\\ \mathrm{\backslash vspace}1mm\frac{a_{4}-2a_{3}+r}{2(a_{4}-a_{3})}, & \text{\textasciitilde{}}a_{3}\leq r\leq a_{4}\\ \;\;\;\;\;\;\;\;\;\;\;\;\;\;\;\;\;\;\;\;\;1, & \;\;\;\;\;\;\;\;\;\;\;\;\;\;\text{\textasciitilde{}}a_{4}\leq r \end{array}\right.\;$$ (14)

This measure evaluates the probability that the fuzzy variable ξ will take on a value less than or equal to a specific real number r. It considers various cases based on the position of r relative to the trapezoidal parameters, reflecting the inherent uncertainty associated with trapezoidal fuzzy numbers.

In a similar fashion, the credibility measure Cr{ξ≥r} for a trapezoidal fuzzy number can be formulated as follows:


$$Cr\{\xi \geq r\}=\left\{ \begin{array}{cc} \;\;\;\;\;\;\;\;\;\;\;\;\;\;\;\;\;1, & \text{\textasciitilde{}}\;\;\;\;\;\;\;\;\;\;\;\;\;\;r\leq a_{1}\\ \mathrm{\backslash vspace}1mm\frac{2a_{2}-r-a_{1}}{2(a_{2}-a_{1})}, & \text{\textasciitilde{}}a_{1}\leq r\leq a_{2}\\ \mathrm{\backslash vspace}1mm\;\;\;\;\;\;\;\;\;\;\;\;\;\;\;\;\;\;\frac{1}{2}, & \text{\textasciitilde{}}a_{2}\leq r\leq a_{3}\\ \frac{a_{4}-r}{2(a_{4}-a_{3})}, & \text{\textasciitilde{}}a_{3}\leq r\leq a_{4}\\ \;\;\;\;\;\;\;\;\;\;\;\;\;\;\;\;0, & \;\;\;\;\;\;\;\;\;\;\;\;\;\text{\textasciitilde{}}a_{4}\leq r \end{array}\right.\;$$ (15)

This measure assesses the probability that the fuzzy variable ξ will assume a value greater than or equal to r. Like the previous measure, it accounts for different scenarios depending on the value of r in relation to the trapezoidal parameters.

**Fig 6:**
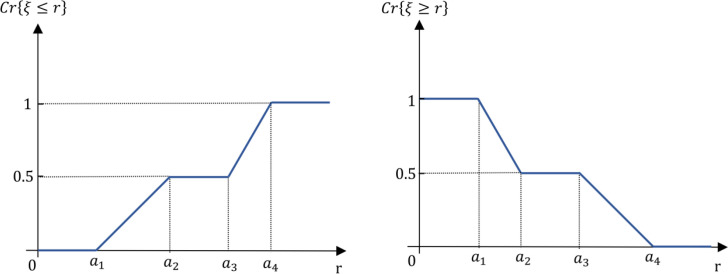
Credibility measures for a trapezoidal fuzzy variable.

Fig 6 visually illustrates these credibility measures, depicting the likelihood of various outcomes in the context of a trapezoidal fuzzy variable. The graphical representation aids in understanding how the credibility measures vary with different values of r, highlighting the uncertainties and potentialities associated with trapezoidal fuzzy numbers.

#### 3.2.2. *Proposed model.*

In this paper, we employ the Credibilistic CVaR criterion, taking into account practical constraints such as cardinality constraints, and floor and ceiling constraints – also known as quantity, threshold, or box constraints. We utilize trapezoidal fuzzy variables to model this Credibilistic CVaR.

In this section, we will detail the model and the modeling process. We begin by introducing the concept of CVaR and explaining its significance in risk management. Following that, we will delve into the specifics of Credibilistic CVaR, highlighting its advantages and how it accommodates uncertainty. Next, we will discuss the various constraints involved in our model, including cardinality constraints that limit the number of selected elements, as well as floor and ceiling constraints that set limits on variable quantities. Finally, we will present the complete model, integrating the Credibilistic CVaR with the identified constraints, to provide a comprehensive framework for decision-making under uncertainty.

#### 3.2.3. Conditional Value at Risk (CVaR).

A key objective of risk management is to evaluate and enhance the performance of financial investments by thoroughly analyzing the associated risks involved in profit generation. One of the most commonly employed methods for quantifying these risks is VaR, which has gained widespread acceptance as a standard tool for estimating potential losses. However, despite its widespread use, VaR has significant limitations, particularly regarding its inability to adequately capture extreme or tail risks—those rare but severe losses that can occur in adverse market conditions. In light of these shortcomings, CVaR has emerged as a more advanced and comprehensive risk assessment measure. Often referred to as mean excess loss, mean shortfall, or tail VaR, CVaR offers a more nuanced understanding of risk by concentrating on the potential losses that surpass the VaR threshold. This focus allows CVaR to provide valuable insights into the tail end of the loss distribution, making it especially relevant in situations where extreme risks are a primary concern.

CVaR is a significant risk assessment tool that goes beyond traditional VaR by concentrating on the tail end of the loss distribution. While VaR provides a measure of the maximum expected loss within a specified confidence level, it falls short by not fully encompassing the potential extreme losses that can occur beyond this threshold. In contrast, CVaR captures the expected loss that exceeds the VaR level, thus offering a more comprehensive perspective on the risks associated with extreme outcomes. This characteristic makes CVaR particularly valuable in situations where tail risk is a critical concern, such as in financial risk management, insurance underwriting, and portfolio optimization strategies. By focusing on the worst-case scenarios, CVaR enables decision-makers to better understand and mitigate the risks of substantial losses. One of the fundamental advantages of CVaR is its coherence, which means it adheres to several desirable mathematical properties, including sub-additivity, translation invariance, monotonicity, and positive homogeneity. The property of sub-additivity is especially important, as it ensures that the risk of a combined portfolio does not exceed the sum of the individual portfolio risks, thereby encouraging diversification. These properties collectively enhance the robustness and reliability of CVaR as a risk assessment measure across various financial contexts. Mathematically, the CVaR of a random variable ξ at a confidence level α can be expressed as:


$$CVaR(x,\eta )=\eta +(1-\alpha {)}^{-1}\int_{\xi \varepsilon R^{n}}\;\;[f(X,\xi )-{\eta ]}^{+}p(\xi )d\xi$$ (16)

In this formulation, $\;[f(X,\xi )-{\eta ]}^{+}$ is expressed as:


$$\begin{array}{c} \;[f(X,\xi )-{\eta ]}^{+\underset{―}{\underset{―}{def}}}\;\left\{ \;\;\;\begin{array}{c} f(X,\xi )-\eta \;if\;f(X,\xi )-\eta >0\\ 0\;if\;f(X,\xi )-\eta \leq 0 \end{array}\right.\; \end{array}$$ (17)

In this context, η represents the VaR threshold, α denotes the confidence level, f(X,ξ) indicates the loss function, and p(ξ) is the probability density function for ξ. This formulation utilizes linear programming techniques to efficiently compute CVaR, making it highly applicable in financial optimization scenarios where understanding and managing risk is critical.

#### 3.2.4. Credibilistic VaR and CV*a*R.

Integrating credibility theory into the computation of CVaR enables a more flexible and nuanced approach to managing uncertainty. Credibility theory is particularly valuable in contexts where data is fuzzy or imprecise, offering an alternative to traditional probability-based methods. By applying this framework, practitioners can better account for the inherent uncertainties in their risk assessments.

Since credibilistic CVaR can be derived from credibilistic VaR, we first need to introduce the concept of credibilistic VaR to facilitate this process. For a fuzzy variable ξ and a confidence level α∈(0, 1], VaR within the context of credibility theory can be defined as:


$$\begin{array}{c} \xi_{VaR}(\alpha )=-sup\{x|Cr\{\xi \leq x\}\leq \alpha \} \end{array}$$ (18)

This definition helps identify the maximum value of x for which the credibility measure Cr{ξ≤x} is less than or equal to the specified confidence level α. In essence, it provides a fuzzy counterpart to the traditional VaR measure, adapting the concept to better handle uncertainty and imprecision inherent in fuzzy data. Moreover, there is an alternative formulation for VaR within the framework of credibility theory:


$$\xi_{VaR}(\alpha )=-inf\left\{x|Cr\left\{\xi \leq x\right\}\geq \alpha \right\}=-inf\left\{x|\Phi (x)\geq \alpha \right\}=-\Phi^{-1}(\alpha )$$ (19)

In this expression, Φ(x) denotes the cumulative credibility distribution function.

Based on the information provided, the VaR for a triangular fuzzy variable defined by the parameters $\xi =(a_{1},\;a_{2},\;a_{3})$ and a confidence level α∈(0, 1] can be determined using the following equations:


$$\xi_{VaR}(\alpha )=\left\{ \;\;\;\begin{array}{c} \begin{array}{cc} 2\left(a_{1}-a_{2}\right)\alpha -a_{1}\; & \alpha \in (0,\;0.5] \end{array}\\ \begin{array}{cc} 2\left(a_{2}-a_{3}\right)\alpha +a_{3}-2a_{2}\; & \alpha \in (0.5,\;1] \end{array} \end{array}\right.\;$$ (20)

Similarly, the VaR for a trapezoidal fuzzy variable defined by the parameters $\xi =\left(a_{1},\;a_{2},\;a_{3},a_{4}\right)$ and a confidence level α∈(0, 1] can be determined using the following equations:


$$\xi_{VaR}(\alpha )=\left\{ \;\;\;\begin{array}{c} \begin{array}{cc} 2\left(a_{1}-a_{2}\right)\alpha -a_{1} & \;\alpha \in (0,\;0.5] \end{array}\\ \begin{array}{cc} 2\left(a_{3}-a_{4}\right)\alpha +a_{4}-2a_{3}\; & \alpha \in (0.5,\;1] \end{array} \end{array}\right.\;$$ (21)

The CVaR within the framework of credibility theory, referred to as $\xi_{CVaR}$, is derived by integrating the VaR function across the confidence interval:


$$\xi_{CVaR}=\frac{1}{1-\alpha }\int_{\alpha }^{1}\;\;\xi_{VaR}(r)dr$$ (22)

Based on the information provided, the CVaR for a triangular fuzzy variable defined by the parameters $\xi =(a_{1},\;a_{2},\;a_{3})$ and a confidence level α∈(0, 1] can be determined using the following equations:


$$\begin{array}{c} \xi_{CVaR}(\alpha )=\left\{ \;\;\;\begin{array}{c} \begin{array}{cc} \alpha a_{1}-(1+\alpha )a_{2} & \alpha \in (0,\;0.5] \end{array}\\ \begin{array}{cc} (\alpha -1)a_{2}-\alpha a_{3} & \;\alpha \in (0.5,\;1] \end{array} \end{array}\right.\; \end{array}$$ (23)

Similarly, the CVaR for a trapezoidal fuzzy variable defined by the parameters $\xi =\left(a_{1},\;a_{2},\;a_{3},a_{4}\right)$ and a confidence level α∈(0, 1] can be determined using the following equations:


$$\xi_{CVaR}(\alpha )=\left\{ \;\;\;\begin{array}{c} \begin{array}{cc} \alpha a_{1}-(1+\alpha )a_{2} & \alpha \in (0,\;0.5] \end{array}\\ \begin{array}{cc} (\alpha -1)a_{3}-\alpha a_{4} & \;\alpha \in (0.5,\;1] \end{array} \end{array}\right.\;$$ (24)

In these formulations (Equations 20–24), different expressions for VaR and CVaR are applied depending on whether the confidence level α falls below or above 0.5.

#### 3.2.5. Additional constraints *for an* effective and realistic portfolio optimization.

In the realm of real-world portfolio optimization, it is crucial to take into account a variety of practical constraints to ensure that the model genuinely reflects the complexities of actual investment scenarios. These constraints play a vital role in shaping investment strategies that are not only theoretically sound but also feasible and relevant to real-life decision-making. By incorporating practical constraints, we can significantly enhance the realism of the portfolio selection process [75]. For example, constraints related to asset selection, risk exposure, and diversification need help to create a more accurate representation of what investors might face in the market. This alignment with practical investment considerations allows investors to make informed decisions that take into account the limitations and requirements of their specific situations. Furthermore, considering these constraints can lead to better risk management. Constraints such as maximum allowable loss, transaction costs, and liquidity requirements ensure that the portfolio not only seeks returns but also protects against potential pitfalls. This dual focus on return maximization and risk minimization is essential for developing a robust investment strategy. In addition, practical constraints can facilitate compliance with regulatory requirements and institutional guidelines, especially concerning cryptocurrencies. Many investors, such as pension funds and mutual funds, are subject to rules that dictate how assets can be allocated. By integrating these constraints into the optimization model, we ensure that the resulting portfolios are compliant with relevant regulations, thus avoiding potential legal issues and enhancing investor confidence.

• *Cardinality constraint:* The cardinality constraint imposes a limit on the number of assets that can be included in a portfolio. This limitation is essential for several reasons, primarily for managing transaction costs and fostering effective portfolio diversification. By restricting the number of assets, investors can avoid the complications and expenses associated with trading too many securities, which can erode potential returns. In practical terms, the selection status of each asset within the portfolio is indicated by a binary variable $Z_{i}$. This means that for each asset, $Z_{i}$ can either be 0 (indicating that the asset is not included in the portfolio) or 1 (indicating that the asset is included). The cardinality constraint can be mathematically expressed as follows:

$$\begin{array}{c} \sum_{i=1}^{N}Z_{i}=K \end{array}$$ (25)

Here, N represents the total number of available assets in the market, while K denotes the maximum number of assets that can be included in the portfolio. This equation ensures that the sum of the binary variables does not exceed the specified limit of K, enforcing the constraint that only a defined number of assets can be selected.

Additionally, the binary variable $Z_{i}\;$must satisfy the following condition:


$$Z_{i}\in \left\{0,1\right\},\;i=1,2,...,N$$ (26)

- *Floor and ceiling constraints:* Floor and ceiling constraints, often referred to as buy-in thresholds, set the minimum and maximum limits for the allocation of portfolio assets. These constraints are critical for maintaining a balanced investment strategy, as they prevent the portfolio from becoming overly concentrated in a single asset and ensure that no asset is allocated an insignificant proportion of the overall investment. By establishing these boundaries, investors can effectively manage their exposure to risk. For instance, a floor constraint ensures that a minimum percentage of the portfolio is invested in a particular asset, while a ceiling constraint caps the maximum percentage that can be allocated. This approach helps to diversify investments and mitigate the potential negative impact of poor performance from any single asset. The mathematical representation of these constraints is expressed as:

$$l_{i}Z_{i}\leq x_{i}\leq u_{i}Z_{i},\;i=1,2,...,N$$ (27)

In this formulation, $x_{i}$ represents the proportion of the portfolio allocated to asset i, while $l_{i}$ and $u_{i}$ denote the lower and upper bounds, respectively, for this allocation. The binary variable $Z_{i}$ indicates whether the asset is included in the portfolio (1) or not (0). Thus, if an asset is not selected (i.e., $Z_{i}=0$), the allocation $x_{i}$ is effectively zero.

Moreover, the following conditions must be satisfied:


$$0\leq l_{i}\leq u_{i}\leq 1$$ (28)

This ensures that the lower bound $l_{i}$ is non-negative and does not exceed the upper bound $u_{i}$, which in turn cannot exceed 1 (or 100% of the portfolio).

*Final structure of proposed portfolio optimization model*

Based on all the information provided, this section will present the final structure of the proposed portfolio optimization model in detail. The paper utilizes a credibilistic CVaR approach with trapezoidal fuzzy variables, enhancing its ability to handle uncertainty in asset returns. In addition, the proposed model incorporates cardinality, floor and ceiling constraints to ensure a balanced and realistic investment strategy. Furthermore, the model restricts the confidence level to the interval α∈(0, 0.5], which ensures that the assessment of risk remains conservative. This conservative approach is designed to mitigate potential losses and enhance the robustness of the portfolio, leading to more informed investment decisions. The final structure of the proposed portfolio optimization model is established as follows:


$$Min\;CVaR=\;\sum_{i=1}^{n}x_{i}\left[{\alpha a}_{1i}-(1+\alpha )a_{2i}\right]$$ (29)

$$S.t.\;\sum_{i=1}^{n}x_{i}\frac{a_{1i}+a_{2i}+a_{3i}+a_{4i}}{4}\geq R$$ (30)

$$\;\sum_{i=1}^{n}x_{i}=1$$ (31)

$${\;l}_{i}Z_{i}\leq x_{i}\leq u_{i}Z_{i}$$ (32)

$$\;\sum_{i=1}^{n}Z_{i}=K$$ (33)

$${\;Z}_{i}=\left\{0,1\right\}$$ (34)

$${\;x}_{i}\geq 0,\;i=1,\;2,\;\ldots ,\;n$$ (35)

In this formulation, Equation (29) specifies the model’s objective, which is to minimize the credibilistic CVaR. Equation (30) introduces a constraint related to expected returns. This constraint ensures that the portfolio achieves a minimum required return R. By doing so, it establishes a necessary balance between risk and return, allowing investors to pursue profit while managing potential downsides. Next, Equation (31) sets a budget constraint, ensuring that the entire budget is fully allocated across the selected assets. This constraint is vital for maintaining the integrity of the investment strategy, as it prevents any portion of the budget from remaining uninvested. Equation (32) outlines the floor and ceiling constraints, which establish minimum and maximum limits on the allocation for each asset within the portfolio. In this context, $Z_{i}$ acts as a binary variable that indicates whether a specific asset is included in the portfolio. This mechanism prevents over-concentration in any single asset and ensures that each asset meets its respective allocation thresholds. Additionally, Equation (33) describes the cardinality constraint, which restricts the number of assets included in the portfolio to a maximum of K. This limitation reflects practical considerations, such as managing transaction costs and maintaining a manageable portfolio size, thereby enhancing the overall efficiency of the investment strategy. Finally, Equations (34) and (35) establish essential binary and non-negativity constraints. Equation (34) mandates that $Z_{i}$ be a binary variable, indicating whether an asset is included in the portfolio (1) or excluded (0). Meanwhile, Equation (35) requires that $x_{i}$, the proportion allocated to each asset, be non-negative. This condition ensures that short positions are not taken, thereby aligning the model with traditional investment practices that typically avoid negative allocations.

## 4. Numerical experiments

This section is devoted to the numerical experiments and provides a systematic presentation of the results, organized step by step. It is divided into two distinct subsections.

The first subsection outlines the outcomes of the asset preselection process, which employs our novel preselection approach. This segment emphasizes the methodology used as well as the results obtained from selecting assets prior to the optimization phase. The second subsection focuses on the portfolio optimization results derived from the assets identified in the earlier stage, specifically utilizing the credibilistic CVaR model. In this part, we will also explore the validation process conducted to ensure the robustness and effectiveness of the proposed model. Together, these subsections provide a comprehensive overview of the two-stage framework for enhancing cryptocurrency portfolio performance, demonstrating its application and effectiveness in real-world scenarios.

### 4.1. *Asset preselection results*

As highlighted in Table 2, we collected data on 47 alternative cryptocurrency assets over the period from December 1, 2023, to December 14, 2024. Furthermore, as detailed in Table 3, we established 11 criteria to evaluate the preselection of these crypto assets. These criteria were carefully chosen to reflect key performance indicators and risk factors that are critical for effective portfolio management in the volatile cryptocurrency market. Using the collected data in conjunction with the specified criteria, we developed a decision matrix, which is presented in Table 5. This matrix serves as a vital tool for comparing the assets based on the established criteria, facilitating informed decision-making in the preselection process.

**Table 5:** Decision matrix for cryptocurrency asset preselection.

Criteria			Avg TPS	Avg Active Addresses	Max Drawdown From ATH	Avg TVL (Billion Dollars)	Avg Fee (Dollars)	Avg Daily Volume (Billion Dollars)	Avg M-Cap (Billion Dollars)	Total Revenue (Million Dollars)	Circulating/Total Supply	Full-Time Developers	Total Developers
Alternatives			Weights										
			11.10%	9.30%	(3.30%)	17.80%	(10.90%)	6.40%	9.00%	18.10%	4.30%	2.50%	7.20%
A_1_	ETH	Ethereum	22.7	6.200	94.32%	21.034	4.135	20.321	250	1950.123	1.000	2788	8865
A_2_	SOL	Solana	1053.7	6.550	97.43%	0.345	0.007	1.534	50.123	100.456	0.782	664	2856
A_3_	TRX	Tron	159	13.625	96.00%	5.532	0.887	1.034	80.456	40.234	0.892	135	952
A_4_	BNB	BSC (Binance)	378.3	11.675	85.53%	2.832	0.103	1.812	350.789	800.789	0.775	556	2,015
A_5_	BTC	Bitcoin	6.71	12.100	84.85%	0.012	15	30.543	650.987	250.345	0.905	358	1,246
A_6_	LINK	Chainlink	7.03	0.047	90.52%	0.011	0.53	0.534	20.234	20.678	0.500	61	158
A_7_	ARB	Arbitrum	59	3.975	75.12%	1.543	0.014	0.312	10.678	15.456	0.128	712	2,530
A_8_	SUI	Sui	854.1	0.039	80.00%	0.002	0.0024	0.026	5.123	5.234	0.053	202	1,108
A_9_	AVAX	Avalanche	89.2	0.688	92.57%	0.634	0.158	0.745	15.456	30.789	0.486	496	1,706
A_10_	POL	Polygon	190.4	6.725	91.08%	0.823	0.022	0.923	30.567	50.123	0.930	834	2,877
A_11_	CRV	Curve	7.56	2.150	95.68%	2.345	0.503	0.035	2.345	10.345	0.227	32	62
A_12_	APT	Aptos	49.5	2.892	78.49%	0.008	0.003	0.423	7.456	8.234	0.200	179	835
A_13_	OP	Optimism	11.8	1.600	76.82%	0.657	0.0114	0.345	6.234	12.789	0.073	466	1,707
A_14_	CORE	CORE	5.49	4.225	82.64%	0.004	0.5013	0.034	1.123	2.123	0.202	16	41
A_15_	UNI	Uniswap	10.95	5.808	88.03%	3.067	4.88	0.645	12.345	60.456	0.753	17	51
A_16_	MNT	Mantle	25.5	0.025	70.16%	0.001	0.45	0.033	0.567	3.234	0.515	30	130
A_17_	CRO	Cronos	72.2	6.700	89.43%	0.134	0.102	0.256	3.234	25.567	0.835	16	81
A_18_	ONDO	Ondo	0.023	10.525	65.29%	0.003	0.345	0.032	0.234	1.123	0.139	17	39
A_19_	NUM	Numbers	0.65	0.003	68.12%	0.001	0.543	0.031	0.123	0.512	0.250	12	24
A_20_	CAKE	Pancakeswap	3.067	1.306	93.55%	0.045	1.86	0.03	0.345	20.678	1.000	14	36
A_21_	MKR	Maker	3.69	0.316	87.43%	5.034	0.693	0.145	2.456	18.345	0.972	25	78
A_22_	RUNE	Thorchain	0.021	0.007	92.43%	0.245	0.123	0.134	0.789	7.234	0.660	18	48
A_23_	TON	TON	175	3.235	74.22%	0.032	0.026	0.029	0.456	40.789	0.244	52	245
A_24_	ADA	Cardano	0.084	4.010	95.07%	0.215	0.153	1.245	40.234	35.123	0.778	217	635
A_25_	GNO	Gnosis	65.6	0.098	80.95%	0.014	0.002	0.028	0.567	5.234	0.260	257	647
A_26_	AAVE	Aave	0.275	0.121	90.46%	4.567	34.76	0.212	1.789	22.789	0.875	23	94
A_27_	AR	Arweave	9.34	0.168	85.44%	0.009	0.397	0.027	0.234	3.123	0.758	46	132
A_28_	DYDX	dYdX	1.53	0.608	88.36%	0.005	0.864	0.123	0.678	10.456	0.065	30	60
A_29_	NEAR	Near	117.8	2.900	91.12%	0.112	0.011	0.113	0.789	15.234	0.900	322	1,214
A_30_	1INCH	1inch	1.018	0.502	89.74%	0.021	0.062	0.103	0.456	8.123	0.400	52	122
A_31_	ROSE	Oasis	2.83	3.889	87.53%	0.003	0.627	0.052	0.234	2.345	0.570	81	382
A_32_	SEI	Sei	1.664	3.945	60.29%	0.002	0.488	0.051	0.123	1.678	0.180	496	1,706
A_33_	ONE	Harmony	8.941	0.041	94.44%	0.011	0.0016	0.049	0.345	6.234	0.935	80	228
A_34_	MANA	Decentraland	0.097	1.340	92.54%	0.004	0.851	0.048	0.456	12.789	0.820	35	50
A_35_	KAVA	Kava	0.276	1.528	86.45%	0.137	0.011	0.047	0.234	4.567	0.750	28	90
A_36_	RON	Ronin	18.65	2.429	72.35%	0.012	0.0031	0.046	0.123	9.345	0.150	12	36
A_37_	VET	Vechain	10.25	4.001	93.50%	0.008	0.056	0.045	0.345	11.678	0.838	22	45
A_38_	LTC	Litecoin	3.582	3.052	90.41%	0.003	0.064	0.044	50.123	20.123	0.869	33	81
A_39_	EOS	EOS	3.493	1.525	95.27%	0.005	0.0124	0.043	0.789	5.456	0.980	37	121
A_40_	CELO	Celo	12.37	0.944	88.44%	0.011	0.0022	0.042	0.234	3.234	0.500	342	1,206
A_41_	FTM	Fantom	59.2	0.470	91.00%	0.157	0.015	0.234	0.345	7.789	0.882	269	1,013
A_42_	EGLD	MultiversX	2.318	0.020	89.65%	0.006	0.065	0.041	0.234	4.123	0.796	57	181
A_43_	STX	Stacks	1.023	1.135	85.38%	0.004	0.104	0.04	0.123	2.234	0.715	55	155
A_44_	XMR	Monero	3.504	0.449	82.14%	0.002	0.053	0.039	1.456	10.789	1.000	30	80
A_45_	ATOM	Cosmos	7.243	0.357	87.56%	0.009	0.034	0.038	0.789	8.456	1.000	683	2,272
A_46_	XTZ	Tezos	2.519	1.495	90.32%	0.005	0.026	0.037	0.567	6.123	1.000	72	228
A_47_	ALGO	Algorand	170.3	1.273	92.18%	0.007	0.0019	0.036	0.234	5.789	0.780	87	444

All data used in this analysis were sourced from a variety of reputable databases, ensuring a comprehensive and diverse dataset. It is worth noting that while most criteria can be directly obtained from these databases, two specific metrics — “Avg Active Addresses” and “Circulating/Total Supply” — were not readily available and required further calculation. The methodologies and calculations for these two criteria are detailed in Appendices 14 and 15, respectively.

As noted, we propose a novel approach for the preselection of cryptocurrency assets that begins with calculating results through a diverse set of methods, specifically utilizing 13 MADM techniques outlined in this paper. The techniques employed in our analysis include MARCOS, CODAS, CoCoSo, EDAS, WASPAS, TOPSIS, MOORA, COPRAS, ARAS, VIKOR, MABAC, MACBETH, and TODIM. Once the results are generated from these methods, they are systematically combined using the Copeland approach, which ensures a comprehensive evaluation by aggregating rankings from multiple decision-making techniques.

To implement this framework effectively, we first need to calculate the outputs of all 13 MADM methods using the data provided in the decision matrix. The results of these calculations are systematically organized and presented in Table 6, which provides a clear and comprehensive overview of the findings.

**Table 6:** Cryptocurrency rankings according to different methods.

Alternative	MARCOS		CODAS		CoCoSo (λ=0.5)		EDAS		WASPAS		TOPSIS		MOORA		COPRAS		ARAS		VIKOR (ϑ=0.5)		MABAC		MACBETH		TODIM (θ=1)	
	Result	Rank	Result	Rank	Result	Rank	Result	Rank	Result	Rank	Result	Rank	Result	Rank	Result	Rank	Result	Rank	Result	Rank	Result	Rank	Result	Rank	Result	Rank
A_1_	0.6423	1	10.331	1	4.5515	1	0.5463	1	0.2843	1	0.7318	1	0.1659	1	0.2537	1	1	1	1.0000	1	0.535	1	66.468	1	1	1
A_2_	0.2863	4	3.179	4	2.7170	5	0.1205	28	0.1489	3	0.3488	3	0.0985	9	0.0644	4	0.2560	4	0.4615	3	0.164	4	27.342	4	0.7580	3
A_3_	0.2419	5	2.146	5	2.7578	4	0.0870	43	0.1040	4	0.3258	4	0.0960	43	0.0482	5	0.1869	5	0.4280	4	0.142	5	25.516	5	0.6277	5
A_4_	0.3336	2	3.643	3	3.2140	2	0.1770	4	0.1621	2	0.4048	2	0.0982	26	0.0962	2	0.3688	2	0.5149	2	0.242	2	33.079	3	0.7607	2
A_5_	0.3264	3	3.822	2	2.8093	3	0.3930	3	0.0611	6	0.3210	5	0.0742	46	0.0908	3	0.3597	3	0.4086	5	0.190	3	37.161	2	0.6757	4
A_6_	0.0527	44	-5.065	44	1.7489	30	0.1114	33	0.0138	36	0.2640	39	0.0970	37	0.0064	36	0.0204	35	0.2854	45	-0.043	44	5.743	38	0.2081	33
A_7_	0.1009	21	-2.229	21	2.0252	11	0.0776	44	0.0424	8	0.2765	9	0.0985	14	0.0183	11	0.0637	15	0.3274	13	0.003	14	7.283	28	0.4181	9
A_8_	0.1932	6	1.917	6	1.7400	34	0.1698	5	0.0321	12	0.3110	6	0.0985	6	0.0315	6	0.1522	6	0.3506	7	0.032	7	11.069	12	0.2609	25
A_9_	0.0740	33	-3.912	35	1.8323	20	0.0753	45	0.0306	14	0.2708	17	0.0980	30	0.0134	16	0.0442	23	0.3056	27	-0.023	28	7.914	23	0.3456	15
A_10_	0.1611	9	-0.350	11	2.3237	6	0.0559	47	0.0681	5	0.2834	8	0.0984	16	0.0255	7	0.0925	8	0.3724	6	0.060	6	15.841	7	0.6275	6
A_11_	0.0684	36	-4.072	38	1.8244	22	0.1033	37	0.0227	22	0.2747	11	0.0971	36	0.0134	18	0.0477	20	0.2949	38	-0.032	37	7.820	24	0.1307	42
A_12_	0.1282	13	-0.941	13	1.9145	15	0.0971	39	0.0345	11	0.2697	18	0.0985	7	0.0134	17	0.0721	14	0.3117	22	-0.017	24	5.875	36	0.3965	11
A_13_	0.0691	35	-4.238	41	1.7413	33	0.1039	36	0.0259	20	0.2692	20	0.0985	12	0.0110	21	0.0370	24	0.2992	31	-0.033	38	4.010	42	0.2991	22
A_14_	0.0635	39	-4.112	39	1.7552	29	0.1258	21	0.0117	38	0.2653	36	0.0971	35	0.0058	39	0.0178	36	0.2976	34	-0.028	31	5.834	37	0.1030	45
A_15_	0.1311	12	-0.893	12	2.1538	7	0.1140	30	0.0426	7	0.2615	44	0.0847	45	0.0213	9	0.0852	10	0.2906	41	0.024	10	14.761	8	0.3647	12
A_16_	0.0556	42	-4.838	43	1.7099	37	0.1283	15	0.0097	45	0.2634	41	0.0972	33	0.0047	42	0.0128	43	0.2980	33	-0.028	32	3.556	44	0.1475	40
A_17_	0.1185	14	-1.202	15	2.1078	8	0.0943	41	0.0362	9	0.2739	12	0.0982	25	0.0139	14	0.0450	22	0.3433	8	0.024	9	12.021	9	0.3539	14
A_18_	0.1095	18	-0.942	14	1.9337	13	0.1385	7	0.0165	31	0.2753	10	0.0975	31	0.0105	22	0.0341	26	0.3430	9	0.027	8	8.180	20	0.1214	43
A_19_	0.0408	46	-5.972	46	1.2719	47	0.1419	6	0.0062	47	0.2620	42	0.0970	38	0.0027	47	0.0059	47	0.2854	44	-0.043	43	1.788	47	0.0000	47
A_20_	0.0765	31	-3.068	30	1.7827	27	0.1235	25	0.0152	33	0.2568	46	0.0932	44	0.0047	43	0.0177	37	0.2858	43	-0.024	29	9.027	17	0.1756	37
A_21_	0.1132	16	-1.312	16	2.0743	9	0.1253	22	0.0306	15	0.2934	7	0.0965	40	0.0230	8	0.0869	9	0.3335	10	0.020	11	11.622	10	0.2974	23
A_22_	0.0551	43	-4.664	42	1.6217	43	0.1279	17	0.0106	44	0.2658	32	0.0981	28	0.0058	40	0.0137	41	0.2912	40	-0.042	42	6.003	34	0.1042	44
A_23_	0.0912	23	-2.751	26	1.9675	12	0.0923	42	0.0281	19	0.2733	13	0.0984	17	0.0158	13	0.0508	18	0.3247	14	-0.001	15	6.809	29	0.3075	20
A_24_	0.1033	19	-1.933	20	2.0305	10	0.0697	46	0.0311	13	0.2720	14	0.0981	29	0.0159	12	0.0539	17	0.3280	12	0.005	12	11.190	11	0.4398	7
A_25_	0.1389	10	0.220	9	1.7462	32	0.1121	31	0.0282	18	0.2678	23	0.0985	4	0.0105	23	0.0783	13	0.2962	36	-0.037	40	4.343	41	0.3328	16
A_26_	0.1030	20	-1.791	19	1.3864	46	0.5274	2	0.0211	25	0.1162	47	0.0348	47	0.0197	10	0.0798	12	0.0000	47	-0.099	47	21.636	6	0.2542	27
A_27_	0.0604	41	-4.218	40	1.7207	36	0.1301	13	0.0113	42	0.2638	40	0.0974	32	0.0046	44	0.0123	45	0.2962	35	-0.031	36	5.946	35	0.1790	36
A_28_	0.0320	47	-6.738	47	1.5845	44	0.1330	8	0.0073	46	0.2608	45	0.0960	42	0.0030	46	0.0089	46	0.2649	46	-0.065	46	3.439	46	0.0339	46
A_29_	0.1143	15	-1.393	18	1.9071	16	0.0963	40	0.0301	17	0.2709	16	0.0985	10	0.0129	19	0.0470	21	0.3292	11	0.005	13	10.354	13	0.4246	8
A_30_	0.0484	45	-5.451	45	1.6790	40	0.1310	11	0.0115	41	0.2655	35	0.0983	22	0.0058	41	0.0128	44	0.2866	42	-0.048	45	4.810	40	0.1381	41
A_31_	0.0786	30	-3.215	32	1.8259	21	0.1098	34	0.0138	35	0.2650	38	0.0967	39	0.0072	31	0.0243	31	0.3062	26	-0.016	23	8.052	21	0.2445	29
A_32_	0.0730	34	-3.640	33	1.6726	41	0.1261	19	0.0116	40	0.2652	37	0.0971	34	0.0067	35	0.0210	34	0.3139	19	-0.008	19	3.894	43	0.2307	32
A_33_	0.1750	8	1.756	7	1.7466	31	0.1282	16	0.0305	16	0.2664	29	0.0985	2	0.0071	32	0.0801	11	0.3020	29	-0.030	34	7.482	27	0.3315	18
A_34_	0.0683	37	-3.709	34	1.7351	35	0.1240	23	0.0117	37	0.2619	43	0.0961	41	0.0046	45	0.0149	39	0.2930	39	-0.029	33	7.722	26	0.1746	38
A_35_	0.0843	28	-2.923	29	1.7907	26	0.1235	24	0.0179	28	0.2668	27	0.0985	10	0.0075	29	0.0254	28	0.3076	25	-0.022	27	6.788	31	0.2363	31
A_36_	0.1103	17	-1.369	17	1.5765	45	0.1234	26	0.0224	23	0.2670	24	0.0985	8	0.0082	26	0.0493	19	0.3015	30	-0.030	35	3.492	45	0.1727	39
A_37_	0.0907	24	-2.414	23	1.8726	18	0.1183	29	0.0186	27	0.2685	21	0.0983	21	0.0087	25	0.0247	30	0.3187	17	-0.008	18	9.540	14	0.2379	30
A_38_	0.0932	22	-2.352	22	1.9336	14	0.1010	38	0.0208	26	0.2693	19	0.0983	23	0.0113	20	0.0346	25	0.3225	15	-0.003	16	9.492	15	0.3113	19
A_39_	0.0900	25	-2.444	24	1.8027	25	0.1261	20	0.0179	29	0.2668	26	0.0985	13	0.0073	30	0.0247	29	0.3094	24	-0.020	26	8.587	18	0.2593	26
A_40_	0.1347	11	-0.143	10	1.6288	42	0.1286	14	0.0236	21	0.2661	31	0.0985	5	0.0070	33	0.0610	16	0.2959	37	-0.037	41	5.645	39	0.2924	24
A_41_	0.0859	26	-2.763	27	1.7698	28	0.1118	32	0.0217	24	0.2678	22	0.0985	15	0.0092	24	0.0301	27	0.3101	23	-0.019	25	7.925	22	0.3326	17
A_42_	0.0620	40	-4.066	37	1.6969	38	0.1318	10	0.0112	43	0.2656	34	0.0983	24	0.0060	38	0.0136	42	0.2982	32	-0.034	39	6.274	32	0.1931	35
A_43_	0.0657	38	-4.013	36	1.6830	39	0.1273	18	0.0116	39	0.2656	33	0.0982	27	0.0063	37	0.0146	40	0.3033	28	-0.027	30	6.242	33	0.1991	34
A_44_	0.0762	32	-3.085	31	1.8210	23	0.1304	12	0.0146	34	0.2662	30	0.0983	20	0.0069	34	0.0163	38	0.3131	21	-0.015	22	6.803	30	0.2483	28
A_45_	0.0813	29	-2.875	28	1.8106	24	0.1320	9	0.0156	32	0.2666	28	0.0984	19	0.0080	27	0.0216	33	0.3133	20	-0.015	21	7.756	25	0.3576	13
A_46_	0.0855	27	-2.657	25	1.8354	19	0.1221	27	0.0169	30	0.2669	25	0.0984	17	0.0079	28	0.0224	32	0.3141	18	-0.014	20	8.332	19	0.3021	21
A_47_	0.1787	7	1.100	8	1.9009	17	0.1062	35	0.0354	10	0.2712	15	0.0985	3	0.0134	15	0.0946	7	0.3195	16	-0.007	17	9.290	16	0.4030	10

Source: Authors’ own computation

**Fig 7:**
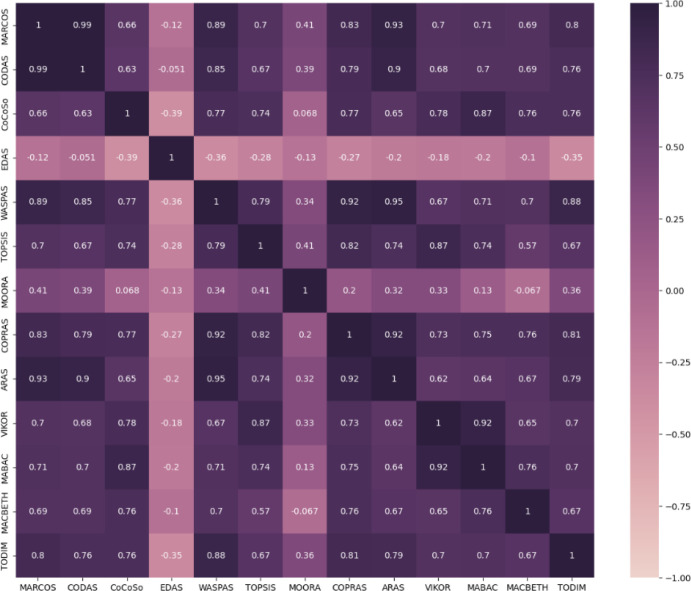
Spearman correlation coefficients among 13 selected MADM methods.

As seen in Table 6, the rankings for each cryptocurrency vary significantly depending on the method used, which can be confusing for investors when selecting the most appropriate approach. For instance, Alternative 33 (ONE, token: Harmony) has the following ranks: 8 in MARCOS, 7 in CODAS, 31 in CoCoSo, 16 in EDAS and WASPAS, 29 in TOPSIS and VIKOR, 2 in MOORA, 32 in COPRAS, 11 in ARAS, 34 in MABAC, 27 in MACBETH, and 18 in TODIM. This wide range of rankings for a single alternative illustrates the inconsistency across different methods, a challenge that extends to other alternatives as well. For more information, the Spearman correlation of these methods is provided in Fig 7. This figure visually represents the correlation coefficients between the various ranking methods, highlighting how closely aligned the rankings are across different approaches. A higher correlation indicates a greater agreement in rankings, while lower values suggest significant discrepancies.

As illustrated in the analysis, there is a significant variation among the various ranking methods employed. This discrepancy highlights the necessity for a robust framework that can effectively synthesize these differing outcomes. Therefore, implementing our proposed method becomes crucial in ensuring a reliable evaluation process.

To address this challenge, we have integrated the results from the 13 selected methods to derive a single comprehensive ranking. This was achieved through three distinct combined approaches: (a) Mean Rank, (b) Borda Count, and (c) Copeland. The outcomes from the Mean Rank method are detailed in Table 7, providing a foundational overview. Following this, Table 8 presents a pairwise matrix of 47 cryptocurrency assets based on the results outlined in Table 6. Table 9 then showcases the results from both the Borda Count and Copeland methods, offering further insights into the rankings derived from these approaches. This integrated methodology aims to provide investors with a clearer and more consistent evaluation framework, enhancing decision-making processes in the ever-evolving landscape of investment opportunities. By reconciling the differences among the various methods, we strive to present a unified perspective that better informs investment strategies.

**Table 7:** Combined results of 13 methods using the mean rank approach.

	MARCOS	CODAS	CoCoSo (λ=0.5)	EDAS	WASPAS	TOPSIS	MOORA	COPRAS	ARAS	VIKOR (ϑ=0.5)	MABAC	MACBETH	TODIM (θ=1)	Mean Rank	Final Rank
A_1_	1	1	1	1	1	1	1	1	1	1	1	1	1	1.00	1
A_2_	4	4	5	28	3	3	9	4	4	3	4	4	3	6.00	3
A_3_	5	5	4	43	4	4	43	5	5	4	5	5	5	10.54	5
A_4_	2	3	2	4	2	2	26	2	2	2	2	3	2	4.15	2
A_5_	3	2	3	3	6	5	46	3	3	5	3	2	4	6.77	4
A_6_	44	44	30	33	36	39	37	36	35	45	44	38	33	38.00	45
A_7_	21	21	11	44	8	9	14	11	15	13	14	28	9	16.77	12
A_8_	6	6	34	5	12	6	6	6	6	7	7	12	25	10.62	6
A_9_	33	35	20	45	14	17	30	16	23	27	28	23	15	25.08	27
A_10_	9	11	6	47	5	8	16	7	8	6	6	7	6	10.92	7
A_11_	36	38	22	37	22	11	36	18	20	38	37	24	42	29.31	33
A_12_	13	13	15	39	11	18	7	17	14	22	24	36	11	18.46	14
A_13_	35	41	33	36	20	20	12	21	24	31	38	42	22	28.85	32
A_14_	39	39	29	21	38	36	35	39	36	34	31	37	45	35.31	39
A_15_	12	12	7	30	7	44	45	9	10	41	10	8	12	19.00	15
A_16_	42	43	37	15	45	41	33	42	43	33	32	44	40	37.69	42
A_17_	14	15	8	41	9	12	25	14	22	8	9	9	14	15.38	10
A_18_	18	14	13	7	31	10	31	22	26	9	8	20	43	19.38	16
A_19_	46	46	47	6	47	42	38	47	47	44	43	47	47	42.08	46
A_20_	31	30	27	25	33	46	44	43	37	43	29	17	37	34.00	37
A_21_	16	16	9	22	15	7	40	8	9	10	11	10	23	15.08	9
A_22_	43	42	43	17	44	32	28	40	41	40	42	34	44	37.69	42
A_23_	23	26	12	42	19	13	17	13	18	14	15	29	20	20.08	18
A_24_	19	20	10	46	13	14	29	12	17	12	12	11	7	17.08	13
A_25_	10	9	32	31	18	23	4	23	13	36	40	41	16	22.77	20
A_26_	20	19	46	2	25	47	47	10	12	47	47	6	27	27.31	30
A_27_	41	40	36	13	42	40	32	44	45	35	36	35	36	36.54	41
A_28_	47	47	44	8	46	45	42	46	46	46	46	46	46	42.69	47
A_29_	15	18	16	40	17	16	10	19	21	11	13	13	8	16.69	11
A_30_	45	45	40	11	41	35	22	41	44	42	45	40	41	37.85	44
A_31_	30	32	21	34	35	38	39	31	31	26	23	21	29	30.00	34
A_32_	34	33	41	19	40	37	34	35	34	19	19	43	32	32.31	35
A_33_	8	7	31	16	16	29	2	32	11	29	34	27	18	20.00	17
A_34_	37	34	35	23	37	43	41	45	39	39	33	26	38	36.15	40
A_35_	28	29	26	24	28	27	10	29	28	25	27	31	31	26.38	28
A_36_	17	17	45	26	23	24	8	26	19	30	35	45	39	27.23	29
A_37_	24	23	18	29	27	21	21	25	30	17	18	14	30	22.85	21
A_38_	22	22	14	38	26	19	23	20	25	15	16	15	19	21.08	19
A_39_	25	24	25	20	29	26	13	30	29	24	26	18	26	24.23	25
A_40_	11	10	42	14	21	31	5	33	16	37	41	39	24	24.92	26
A_41_	26	27	28	32	24	22	15	24	27	23	25	22	17	24.00	24
A_42_	40	37	38	10	43	34	24	38	42	32	39	32	35	34.15	38
A_43_	38	36	39	18	39	33	27	37	40	28	30	33	34	33.23	36
A_44_	32	31	23	12	34	30	20	34	38	21	22	30	28	27.31	30
A_45_	29	28	24	9	32	28	19	27	33	20	21	25	13	23.69	22
A_46_	27	25	19	27	30	25	17	28	32	18	20	19	21	23.69	22
A_47_	7	8	17	35	10	15	3	15	7	16	17	16	10	13.54	8

Source: Authors’ own computation

**Table 8:** Pairwise comparison matrix of 47 cryptocurrency assets.

	1	2	3	4	5	6	7	8	9	10	11	12	13	14	15	16	17	18	19	20	21	22	23	24	25	26	27	28	29	30	31	32	33	34	35	36	37	38	39	40	41	42	43	44	45	46	47
**1**	-	1	1	1	1	1	1	1	1	1	1	1	1	1	1	1	1	1	1	1	1	1	1	1	1	1	1	1	1	1	1	1	1	1	1	1	1	1	1	1	1	1	1	1	1	1	1
**2**	0	-	1	0	0	1	1	1	1	1	1	1	1	1	1	1	1	1	1	1	1	1	1	1	1	1	1	1	1	1	1	1	1	1	1	1	1	1	1	1	1	1	1	1	1	1	1
**3**	0	0	-	0	0	1	1	1	1	1	1	1	1	1	1	1	1	1	1	1	1	1	1	1	1	1	1	1	1	1	1	1	1	1	1	1	1	1	1	1	1	1	1	1	1	1	1
**4**	0	1	1	-	1	1	1	1	1	1	1	1	1	1	1	1	1	1	1	1	1	1	1	1	1	1	1	1	1	1	1	1	1	1	1	1	1	1	1	1	1	1	1	1	1	1	1
**5**	0	1	1	0	-	1	1	1	1	1	1	1	1	1	1	1	1	1	1	1	1	1	1	1	1	1	1	1	1	1	1	1	1	1	1	1	1	1	1	1	1	1	1	1	1	1	1
**6**	0	0	0	0	0	-	0	0	0	0	0	0	0	0	0	1	0	0	1	0	0	0	0	0	0	0	0	1	0	1	0	0	0	1	0	0	0	0	0	0	0	0	0	0	0	0	0
**7**	0	0	0	0	0	1	-	0	1	0	1	1	1	1	0	1	0	1	1	1	0	1	1	0	1	1	1	1	0	1	1	1	1	1	1	1	1	1	1	1	1	1	1	1	1	1	1
**8**	0	0	0	0	0	1	1	-	1	1	1	1	1	1	1	1	1	1	1	1	1	1	1	1	1	1	1	1	1	1	1	1	1	1	1	1	1	1	1	1	1	1	1	1	1	1	1
**9**	0	0	0	0	0	1	0	0	-	0	1	0	1	1	0	1	0	0	1	1	0	1	0	0	1	1	1	1	0	1	1	1	1	1	1	1	0	0	0	1	0	1	1	1	0	0	0
10	0	0	0	0	0	1	1	0	1	-	1	1	1	1	1	1	1	1	1	1	1	1	1	1	1	1	1	1	1	1	1	1	1	1	1	1	1	1	1	1	1	1	1	1	1	1	1
11	0	0	0	0	0	1	0	0	0	0	-	0	1	1	0	1	0	0	1	1	0	1	0	0	0	0	1	1	0	1	0	0	0	1	0	0	0	0	0	0	0	1	1	0	0	0	0
12	0	0	0	0	0	1	0	0	1	0	1	-	1	1	0	1	0	1	1	1	0	1	1	0	1	1	1	1	1	1	1	1	1	1	1	1	1	1	1	1	1	1	1	1	1	1	0
13	0	0	0	0	0	1	0	0	0	0	0	0	-	1	0	1	0	0	1	1	0	1	0	0	0	1	1	1	0	1	0	1	0	1	0	0	0	0	0	1	0	1	1	0	0	0	0
14	0	0	0	0	0	1	0	0	0	0	0	0	0	-	0	1	0	0	1	0	0	1	0	0	0	0	1	1	0	1	0	0	0	1	0	0	0	0	0	0	0	0	0	0	0	0	0
15	0	0	0	0	0	1	1	0	1	0	1	1	1	1	-	1	1	1	1	1	1	1	1	1	1	1	1	1	1	1	1	1	1	1	1	1	1	1	1	1	1	1	1	1	1	1	0
16	0	0	0	0	0	0	0	0	0	0	0	0	0	0	0	-	0	0	1	0	0	0	0	0	0	0	0	1	0	1	0	0	0	0	0	0	0	0	0	0	0	0	0	0	0	0	0
17	0	0	0	0	0	1	1	0	1	0	1	1	1	1	0	1	-	1	1	1	1	1	1	1	1	1	1	1	1	1	1	1	1	1	1	1	1	1	1	1	1	1	1	1	1	1	1
18	0	0	0	0	0	1	0	0	1	0	1	0	1	1	0	1	0	-	1	1	0	1	1	0	1	1	1	1	0	1	1	1	1	1	1	1	1	1	1	1	1	1	1	1	1	1	0
19	0	0	0	0	0	0	0	0	0	0	0	0	0	0	0	0	0	0	-	0	0	0	0	0	0	0	0	1	0	0	0	0	0	0	0	0	0	0	0	0	0	0	0	0	0	0	0
20	0	0	0	0	0	1	0	0	0	0	0	0	0	1	0	1	0	0	1	-	0	1	0	0	0	0	1	1	0	1	0	0	0	1	0	0	0	0	0	0	0	1	1	0	0	0	0
21	0	0	0	0	0	1	1	0	1	0	1	1	1	1	0	1	0	1	1	1	-	1	1	1	1	1	1	1	1	1	1	1	1	1	1	1	1	1	1	1	1	1	1	1	1	1	1
22	0	0	0	0	0	1	0	0	0	0	0	0	0	0	0	1	0	0	1	0	0	-	0	0	0	0	0	1	0	1	0	0	0	0	0	0	0	0	0	0	0	0	0	0	0	0	0
23	0	0	0	0	0	1	0	0	1	0	1	0	1	1	0	1	0	0	1	1	0	1	-	0	0	1	1	1	0	1	1	1	0	1	1	1	1	1	1	1	1	1	1	1	1	1	0
24	0	0	0	0	0	1	1	0	1	0	1	1	1	1	0	1	0	1	1	1	0	1	1	-	1	1	1	1	1	1	1	1	1	1	1	1	1	1	1	1	1	1	1	1	1	1	1
25	0	0	0	0	0	1	0	0	0	0	1	0	1	1	0	1	0	0	1	1	0	1	1	0	-	1	1	1	0	1	1	1	0	1	1	1	1	1	1	1	1	1	1	1	1	1	0
26	0	0	0	0	0	1	0	0	0	0	1	0	0	1	0	1	0	0	1	1	0	1	0	0	0	-	1	1	0	1	1	1	0	1	1	0	1	1	1	0	0	1	1	1	1	1	0
27	0	0	0	0	0	1	0	0	0	0	0	0	0	0	0	1	0	0	1	0	0	1	0	0	0	0	-	1	0	1	0	0	0	0	0	0	0	0	0	0	0	0	0	0	0	0	0
28	0	0	0	0	0	0	0	0	0	0	0	0	0	0	0	0	0	0	0	0	0	0	0	0	0	0	0	-	0	0	0	0	0	0	0	0	0	0	0	0	0	0	0	0	0	0	0
29	0	0	0	0	0	1	1	0	1	0	1	0	1	1	0	1	0	1	1	1	0	1	1	0	1	1	1	1	-	1	1	1	1	1	1	1	1	1	1	1	1	1	1	1	1	1	0
30	0	0	0	0	0	0	0	0	0	0	0	0	0	0	0	0	0	0	1	0	0	0	0	0	0	0	0	1	0	-	0	0	0	0	0	0	0	0	0	0	0	0	0	0	0	0	0
31	0	0	0	0	0	1	0	0	0	0	1	0	1	1	0	1	0	0	1	1	0	1	0	0	0	0	1	1	0	1	-	1	0	1	0	0	0	0	0	0	0	1	1	0	0	0	0
32	0	0	0	0	0	1	0	0	0	0	1	0	0	1	0	1	0	0	1	1	0	1	0	0	0	0	1	1	0	1	0	-	0	1	0	0	0	0	0	0	0	1	1	0	0	0	0
33	0	0	0	0	0	1	0	0	0	0	1	0	1	1	0	1	0	0	1	1	0	1	1	0	1	1	1	1	0	1	1	1	-	1	1	1	1	1	1	1	0	1	1	1	0	1	0
34	0	0	0	0	0	0	0	0	0	0	0	0	0	0	0	1	0	0	1	0	0	1	0	0	0	0	1	1	0	1	0	0	0	-	0	0	0	0	0	0	0	1	0	0	0	0	0
35	0	0	0	0	0	1	0	0	0	0	1	0	1	1	0	1	0	0	1	1	0	1	0	0	0	0	1	1	0	1	1	1	0	1	-	0	0	0	0	0	0	1	1	1	0	0	0
36	0	0	0	0	0	1	0	0	0	0	1	0	1	1	0	1	0	0	1	1	0	1	0	0	0	1	1	1	0	1	1	1	0	1	1	-	0	0	1	0	0	1	1	1	1	1	0
37	0	0	0	0	0	1	0	0	1	0	1	0	1	1	0	1	0	0	1	1	0	1	0	0	0	0	1	1	0	1	1	1	0	1	1	1	-	0	1	0	1	1	1	1	1	1	0
38	0	0	0	0	0	1	0	0	1	0	1	0	1	1	0	1	0	0	1	1	0	1	0	0	0	0	1	1	0	1	1	1	0	1	1	1	1	-	1	1	1	1	1	1	1	1	0
39	0	0	0	0	0	1	0	0	1	0	1	0	1	1	0	1	0	0	1	1	0	1	0	0	0	0	1	1	0	1	1	1	0	1	1	0	0	0	-	0	0	1	1	1	1	1	0
40	0	0	0	0	0	1	0	0	0	0	1	0	0	1	0	1	0	0	1	1	0	1	0	0	0	1	1	1	0	1	1	1	0	1	1	1	1	0	1	-	0	1	1	1	0	0	0
41	0	0	0	0	0	1	0	0	1	0	1	0	1	1	0	1	0	0	1	1	0	1	0	0	0	1	1	1	0	1	1	1	1	1	1	1	0	0	1	1	-	1	1	1	1	1	0
42	0	0	0	0	0	1	0	0	0	0	0	0	0	1	0	1	0	0	1	0	0	1	0	0	0	0	1	1	0	1	0	0	0	0	0	0	0	0	0	0	0	-	0	0	0	0	0
43	0	0	0	0	0	1	0	0	0	0	0	0	0	1	0	1	0	0	1	0	0	1	0	0	0	0	1	1	0	1	0	0	0	1	0	0	0	0	0	0	0	1	-	0	0	0	0
44	0	0	0	0	0	1	0	0	0	0	1	0	1	1	0	1	0	0	1	1	0	1	0	0	0	0	1	1	0	1	1	1	0	1	0	0	0	0	0	0	0	1	1	-	0	0	0
45	0	0	0	0	0	1	0	0	1	0	1	0	1	1	0	1	0	0	1	1	0	1	0	0	0	0	1	1	0	1	1	1	1	1	1	0	0	0	0	1	0	1	1	1	-	0	0
46	0	0	0	0	0	1	0	0	1	0	1	0	1	1	0	1	0	0	1	1	0	1	0	0	0	0	1	1	0	1	1	1	0	1	1	0	0	0	0	1	0	1	1	1	1	-	0
47	0	0	0	0	0	1	0	0	1	0	1	1	1	1	1	1	0	1	1	1	0	1	1	0	1	1	1	1	1	1	1	1	1	1	1	1	1	1	1	1	1	1	1	1	1	1	-

Source: Authors’ own computation

**Table 9:** Combined results of 13 methods using the Borda count and Copeland approaches.

Alternative			Total Wins	Total Losses	Difference between Wins and Losses	Borda Count	Copeland
A_1_	ETH	Ethereum	46	0	46	1	1
A_2_	SOL	Solana	43	3	40	4	4
A_3_	TRX	Tron	42	4	38	5	5
A_4_	BNB	BSC (Binance)	45	1	44	2	2
A_5_	BTC	Bitcoin	44	2	42	3	3
A_6_	LINK	Chainlink	5	41	-36	42	42
A_7_	ARB	Arbitrum	34	12	22	13	13
A_8_	SUI	Sui	41	5	36	6	6
A_9_	AVAX	Avalanche	23	23	0	23	23
A_10_	POL	Polygon	40	6	34	7	7
A_11_	CRV	Curve	13	33	-20	34	34
A_12_	APT	Aptos	33	13	20	14	14
A_13_	OP	Optimism	15	31	-16	32	32
A_14_	CORE	CORE	8	38	-30	38	38
A_15_	UNI	Uniswap	38	8	30	8	8
A_16_	MNT	Mantle	3	43	-40	44	44
A_17_	CRO	Cronos	38	8	30	8	8
A_18_	ONDO	Ondo	31	15	16	16	16
A_19_	NUM	Numbers	1	45	-44	46	46
A_20_	CAKE	Pancakeswap	11	35	-24	36	36
A_21_	MKR	Maker	37	9	28	10	10
A_22_	RUNE	Thorchain	5	41	-36	42	42
A_23_	TON	TON	28	18	10	17	17
A_24_	ADA	Cardano	36	10	26	11	11
A_25_	GNO	Gnosis	28	18	10	17	17
A_26_	AAVE	Aave	22	24	-2	24	24
A_27_	AR	Arweave	6	40	-34	41	41
A_28_	DYDX	dYdX	0	46	-46	47	47
A_29_	NEAR	Near	33	13	20	14	14
A_30_	1INCH	1inch	2	44	-42	45	45
A_31_	ROSE	Oasis	15	31	-16	32	32
A_32_	SEI	Sei	13	33	-20	34	34
A_33_	ONE	Harmony	27	19	8	19	19
A_34_	MANA	Decentraland	7	39	-32	40	40
A_35_	KAVA	Kava	17	29	-12	30	30
A_36_	RON	Ronin	22	24	-2	24	24
A_37_	VET	Vechain	24	22	2	22	22
A_38_	LTC	Litecoin	26	20	6	20	20
A_39_	EOS	EOS	21	25	-4	26	26
A_40_	CELO	Celo	21	25	-4	26	26
A_41_	FTM	Fantom	26	20	6	20	20
A_42_	EGLD	MultiversX	8	38	-30	38	38
A_43_	STX	Stacks	10	36	-26	37	37
A_44_	XMR	Monero	16	30	-14	31	31
A_45_	ATOM	Cosmos	21	25	-4	26	26
A_46_	XTZ	Tezos	21	25	-4	26	26
A_47_	ALGO	Algorand	35	11	24	12	12

Source: Authors’ own computation

In Table 7, we calculated the mean rank of each asset, providing a foundational metric to assess their performance relative to one another. This mean rank serves as a critical indicator of each asset’s standing, with lower values reflecting better rankings.

Subsequently, we constructed a Pairwise Comparison Matrix in Table 8. This matrix serves as a detailed framework for evaluating the relative performance of each asset by comparing them in pairs. For each asset pair, we assessed their standings based on the number of better ranks they received across the 13 selected methods. In this comparison, the asset that received a greater number of better ranks was designated as a win (1). Conversely, the asset with fewer better ranks was classified as a loss (0). For instance, in this paper, consider two assets: if one asset ranked better than the other in at least 7 of the 13 methods, it would be classified as a win (1). On the other hand, if the same asset had a lower count of better ranks compared to its counterpart, it would be classified as a loss (0).

After completing Table 8, the Pairwise Comparison Matrix, we analyzed the results to derive meaningful insights. The sum of each row corresponds to the total wins for that specific asset, reflecting the number of other assets it has outperformed. Meanwhile, the sum of each column indicates the total losses, showcasing how many assets surpassed it in rank. This dual perspective provides a comprehensive view of each asset’s competitive standing. With this data in hand, we can proceed to calculate the Borda Count and Copeland scores. The Borda Count method uses the total number of wins for each asset; thus, an asset with a higher total win’s count is assigned a better rank. In contrast, the Copeland score is derived from the difference between wins and losses—each asset is ranked more favorably if it has a higher positive difference. This approach highlights not only the wins but also the impact of losses on the asset’s overall standing. Finally, Table 9 presents the results of the Borda Count and Copeland methods, offering deeper insights into the rankings derived from this comprehensive analysis.

Based on the results presented in Table 8, we constructed the Pairwise Comparison Matrix, enabling us to calculate the total wins and losses for each asset. This matrix serves as a foundational tool for evaluating asset performance, allowing for a systematic comparison. Utilizing this data, we derived the rankings based on both the Borda count and Copeland methods, which are summarized in Table 9. Importantly, in this study, the rankings from both methods are identical due to the use of an odd number of comparison methods (13 methods).

When the number of methods is odd, the outcomes are limited to two possibilities: a win (1) or a loss (0). A win occurs when an asset ranks higher than its counterpart in at least 7 of the 13 methods, while a loss is assigned if it ranks lower. In this context, it becomes evident that an asset with a higher number of wins will correspondingly have fewer losses, resulting in a significant difference between the wins and losses. Consequently, the Borda count, which focuses solely on the tally of wins, aligns perfectly with Copeland’s method, which takes into account the overall difference between wins and losses. Conversely, the scenario shifts when the number of methods is even. In such cases, we encounter three potential outcomes: win (1), loss (0), and draw (0). For example, if there are 12 methods and asset i ranks better than asset j in 6 methods, while asset j ranks better than asset i in the remaining 6, this situation results in a draw. This complicates our ability to ascertain that an asset with a greater number of wins necessarily has fewer losses, as the presence of draws introduces ambiguity into the comparisons. As a result, we would expect the Borda count to yield different outcomes compared to Copeland’s method in this context. Given these considerations, Copeland’s approach emerges as the more robust method for consolidating results, as it effectively accounts for both wins and losses. Although our findings reveal that the results from the Borda count and Copeland are consistent in this study (attributable to the 13 methods utilized), we aimed to present a comprehensive framework in this paper. This framework serves as a benchmark for readers who may explore various scenarios in future analyses. It is crucial to emphasize that if the number of methods changes, particularly to an even count, the effectiveness of the Borda count for making accurate comparisons may be diminished. Additionally, the summarized results from the Mean Rank, Borda Count, and Copeland methods are presented in Table A3 (Appendix 16). This table illustrates that while both the Borda count and Copeland methods may yield similar rankings under certain conditions, they can differ significantly from the mean rank approach, underscoring the importance of selecting an appropriate method based on the specific context of the analysis.

As previously noted, we employed the Copeland method for our comparisons. The results derived from this approach are displayed in Table 10, where we have categorized the assets into five distinct groups: excellent, satisfactory, average, below average, and poor. Each category is visually differentiated by a specific color in the table, enhancing clarity and facilitating quick assessment.

**Table 10:** Categorization of cryptocurrency assets based on Copeland method results.

Alternative			Final Rank	Alternative			Final Rank
A_1_	ETH	Ethereum	1	A_36_	RON	Ronin	24
A_4_	BNB	BSC (Binance)	2	A_39_	EOS	EOS	26
A_5_	BTC	Bitcoin	3	A_40_	CELO	Celo	26
A_2_	SOL	Solana	4	A_45_	ATOM	Cosmos	26
A_3_	TRX	Tron	5	A_46_	XTZ	Tezos	26
A_8_	SUI	Sui	6	A_35_	KAVA	Kava	30
A_10_	POL	Polygon	7	A_44_	XMR	Monero	31
A_15_	UNI	Uniswap	8	A_13_	OP	Optimism	32
A_17_	CRO	Cronos	8	A_31_	ROSE	Oasis	32
A_21_	MKR	Maker	10	A_11_	CRV	Curve	34
A_24_	ADA	Cardano	11	A_32_	SEI	Sei	34
A_47_	ALGO	Algorand	12	A_20_	CAKE	Pancakeswap	36
A_7_	ARB	Arbitrum	13	A_43_	STX	Stacks	37
A_12_	APT	Aptos	14	A_14_	CORE	CORE	38
A_29_	NEAR	Near	14	A_42_	EGLD	MultiversX	38
A_18_	ONDO	Ondo	16	A_34_	MANA	Decentraland	40
A_23_	TON	TON	17	A_27_	AR	Arweave	41
A_25_	GNO	Gnosis	17	A_6_	LINK	Chainlink	42
A_33_	ONE	Harmony	19	A_22_	RUNE	Thorchain	42
A_38_	LTC	Litecoin	20	A_16_	MNT	Mantle	44
A_41_	FTM	Fantom	20	A_30_	1INCH	1inch	45
A_37_	VET	Vechain	22	A_19_	NUM	Numbers	46
A_9_	AVAX	Avalanche	23	A_28_	DYDX	dYdX	47
A_26_	AAVE	Aave	24				

Source: Authors’ own computation

The categorization of assets into distinct groups enhances clarity and provides a clear guide for asset evaluation. Each category—excellent, satisfactory, average, below average, and poor—has its own economic significance. The “excellent” category represents the top-performing assets, indicating their strong potential for high returns and stability, making them ideal candidates for inclusion in a portfolio focused on maximizing performance. On the other hand, assets categorized as “poor” highlight those with lower expected returns or higher associated risks, serving as a signal for investors to avoid or limit exposure. By utilizing these categories, investors can align their portfolios more effectively with their financial goals, risk preferences, and market conditions.

In the subsequent steps of our analysis, we will focus on the assets classified as excellent (Top 10 assets), as this category contains high-quality assets with strong potential for superior performance. By concentrating on these high-quality assets, we have effectively conducted a preselection of the leading cryptocurrency options, ensuring that only the most promising candidates are considered for our investment strategy. This targeted focus on high-quality assets allows for a more refined and informed investment approach, ensuring that the portfolio remains aligned with the investor’s risk-return objectives.

### 4.2. Portfolio optimization results

Following the preselection of assets, which outlines the results of Stage 1, this section focuses on the portfolio optimization process, specifically regarding asset weight allocation. In this stage, we will allocate weights to the assets classified as excellent, specifically the top 10 assets, under various scenarios. These scenarios will be constructed by varying the values assigned to the cardinality constraints, enabling us to create portfolios of different sizes tailored to the preferences and risk appetites of diverse investors. To verify the efficiency of our proposed model and validate our two-stage framework, we will evaluate the portfolios generated using the top 10 assets against those developed using larger groups, including the top 20, 30, 40, and all 47 assets. For each of these portfolios, we will establish scenarios analogous to our own results, ensuring that the comparisons are meaningful and relevant. This validation process will provide insights into the effectiveness of our preselection methodology and its impact on portfolio performance. In this section, we will detail the results step by step, illustrating the implications of our findings.

As previously highlighted, we employ the Credibilistic CVaR criterion, which incorporates practical constraints into our analysis. To accurately model this Credibilistic CVaR, we utilize trapezoidal fuzzy variables. The initial step in this process involves gathering trapezoidal fuzzy variables for each asset, which are derived from expert opinions and compiled in Table 11. This data will serve as a foundational element for our analysis, allowing us to assess the potential risks and returns associated with the selected portfolios.

**Table 11:** Trapezoidal fuzzy representation of cryptocurrency returns expectations.

Rank	Alternative		Trapezoidal Fuzzy Data	Rank	Alternative		Trapezoidal Fuzzy Data
$x_{1}$	A_1_	ETH	(-0.101, -0.028, 0.118, 0.19)	$x_{25}$	A_36_	RON	(-0.181, -0.093, 0.082, 0.17)
$x_{2}$	A_4_	BNB	(-0.085, -0.023, 0.103, 0.165)	$x_{26}$	A_39_	EOS	(-0.216, -0.102, 0.125, 0.239)
$x_{3}$	A_5_	BTC	(-0.082, -0.031, 0.071, 0.123)	$x_{27}$	A_40_	CELO	(-0.189, -0.016, 0.33, 0.503)
$x_{4}$	A_2_	SOL	(-0.136, -0.066, 0.074, 0.144)	$x_{28}$	A_45_	ATOM	(-0.169, -0.08, 0.099, 0.189)
$x_{5}$	A_3_	TRX	(-0.209, 0.065, 0.614, 0.889)	$x_{29}$	A_46_	XTZ	(-0.182, -0.015, 0.32, 0.488)
$x_{6}$	A_8_	SUI	(-0.166, -0.026, 0.253, 0.393)	$x_{30}$	A_35_	KAVA	(-0.204, -0.109, 0.082, 0.178)
$x_{7}$	A_10_	POL	(-0.17, -0.089, 0.073, 0.154)	$x_{31}$	A_44_	XMR	(-0.366, -0.215, 0.087, 0.238)
$x_{8}$	A_15_	UNI	(-0.141, 0.029, 0.368, 0.538)	$x_{32}$	A_13_	OP	(-0.165, -0.025, 0.254, 0.394)
$x_{9}$	A_17_	CRO	(-0.148, 0.056, 0.465, 0.67)	$x_{33}$	A_31_	ROSE	(-0.195, -0.1, 0.091, 0.186)
$x_{10}$	A_21_	MKR	(-0.145, -0.05, 0.141, 0.237)	$x_{34}$	A_11_	CRV	(-0.204, -0.084, 0.154, 0.273)
$x_{11}$	A_24_	ADA	(-0.159, -0.062, 0.131, 0.228)	$x_{35}$	A_32_	SEI	(-0.182, -0.069, 0.158, 0.271)
$x_{12}$	A_47_	ALGO	(-0.159, -0.025, 0.242, 0.376)	$x_{36}$	A_20_	CAKE	(-0.197, -0.095, 0.109, 0.21)
$x_{13}$	A_7_	ARB	(-0.171, -0.067, 0.14, 0.244)	$x_{37}$	A_43_	STX	(-0.173, -0.027, 0.265, 0.411)
$x_{14}$	A_12_	APT	(-0.176, -0.07, 0.142, 0.248)	$x_{38}$	A_14_	CORE	(-0.291, -0.001, 0.579, 0.869)
$x_{15}$	A_29_	NEAR	(-0.171, -0.036, 0.233, 0.368)	$x_{39}$	A_42_	EGLD	(-0.194, -0.112, 0.052, 0.134)
$x_{16}$	A_18_	ONDO	(-0.144, -0.012, 0.252, 0.384)	$x_{40}$	A_34_	MANA	(-0.188, -0.045, 0.241, 0.384)
$x_{17}$	A_23_	TON	(-0.15, -0.055, 0.135, 0.229)	$x_{41}$	A_27_	AR	(-0.203, -0.024, 0.333, 0.512)
$x_{18}$	A_25_	GNO	(-0.123, -0.04, 0.127, 0.21)	$x_{42}$	A_6_	LINK	(-0.15, -0.032, 0.204, 0.322)
$x_{19}$	A_33_	ONE	(-0.198, -0.084, 0.144, 0.258)	$x_{43}$	A_22_	RUNE	(-0.182, -0.058, 0.191, 0.315)
$x_{20}$	A_38_	LTC	(-0.184, -0.091, 0.094, 0.186)	$x_{44}$	A_16_	MNT	(-0.101, 0.007, 0.222, 0.33)
$x_{21}$	A_41_	FTM	(-0.189, -0.068, 0.174, 0.294)	$x_{45}$	A_30_	1INCH	(-0.235, -0.115, 0.125, 0.245)
$x_{22}$	A_37_	VET	(-0.166, -0.032, 0.237, 0.372)	$x_{46}$	A_19_	NUM	(-0.228, 0.116, 0.805, 1.15)
$x_{23}$	A_9_	AVAX	(-0.166, -0.026, 0.253, 0.393)	$x_{47}$	A_28_	DYDX	(-0.22, -0.087, 0.18, 0.314)
$x_{24}$	A_26_	AAVE	(-0.172, -0.059, 0.169, 0.282)				

Source: Authors’ own compilation

For the optimization process, we utilized the data presented in Table 11, along with the Credibilistic CVaR framework defined in Equations 29–35. We employed GAMS software to solve the optimization model, carefully setting the parameters as follows: α=0.05, an expected return (R) of 10%, a floor ($l_{i}$) of 0.1, and a ceiling ($u_{i}$) of 0.5. The cardinality constraints were established for K values of 3, 5, and 7, allowing us to explore different portfolio sizes. After solving the model, we constructed three distinct portfolios using the top 10 assets, each corresponding to the specified cardinality constraints. For instance, when K=3, the selected asset allocations were: A_3_ (TRX) at 50% (ranked 5), A_15_ (UNI) at 10% (ranked 8), and A_17_ (CRO) at 40% (ranked 9). These allocations reflect a strategic selection aimed at optimizing the risk-return profile within the set of constraints. The detailed results of these portfolios, including the asset weights and corresponding ranks, are summarized in Table 12. This comprehensive analysis allows us to assess how different cardinality constraints influence portfolio composition and performance.

**Table 12:** Asset weight allocations for top 10 assets under different cardinality constraints.

No.	k	Objective Function	$x_{1}$	$x_{2}$	$x_{3}$	$x_{5}$	$x_{6}$	$x_{8}$	$x_{9}$
			A_1_ (ETH)	A_4_ (BNB)	A_5_ (BTC)	A_3_ (TRX)	A_8_ (SUI)	A_15_ (UNI)	A_17_ (CRO)
Scenario 1	k=3	-0.070	–	–	–	50%	–	10%	40%
Scenario 2	k=5	-0.052	–	10%	–	50%	10%	10%	20%
Scenario 3	k=7	-0.033	10%	10%	10%	40%	10%	10%	10%

Source: Authors’ own computation

To verify the efficiency of our proposed model and validate our two-stage framework, we expanded the scope of our analysis by generating additional portfolios using larger asset groups. Specifically, we examined the top 20, 30, 40, and all 47 assets, allowing for a comprehensive evaluation across multiple scenarios. The results of these portfolio constructions are detailed in Table 13. By analyzing these larger groups, we can better understand the dynamics of risk and return when more assets are considered. This broader perspective helps us assess the robustness of our model, particularly in terms of its adaptability and performance under different conditions.

**Table 13:** Asset weight allocations for larger asset groups: top 20, 30, 40, and 47.

Input Data: Top 20 Assets ($x_{1}$ to $x_{20})$									
No.	k	Objective Function	$x_{2}$	$x_{5}$	$x_{6}$	$x_{8}$	$x_{9}$	$x_{12}$	$x_{16}$
			A_4_ (BNB)	A_3_ (TRX)	A_8_ (SUI)	A_15_ (UNI)	A_17_ (CRO)	A_47_ (ALGO)	A_18_ (ONDO)
Scenario 1	k=3	-0.070	–	50%	–	10%	40%	–	–
Scenario 2	k=5	-0.054	–	50%	–	10%	20%	10%	10%
Scenario 3	k=7	-0.036	10%	40%	10%	10%	10%	10%	10%
**Input Data: Top 30 Assets (**$x_{1}$ **to** $x_{30})$									
No.	k	Objective Function	$x_{5}$	$x_{8}$	$x_{9}$	$x_{12}$	$x_{16}$	$x_{27}$	$x_{29}$
			A_3_ (TRX)	A_15_ (UNI)	A_17_ (CRO)	A_47_ (ALGO)	A_18_ (ONDO)	A_40_ (CELO)	A_46_ (XTZ)
Scenario 1	k=3	-0.070	50%	10%	40%	–	–	–	–
Scenario 2	k=5	-0.055	50%	10%	20%	–	10%	–	10%
Scenario 3	k=7	-0.038	40%	10%	10%	10%	10%	10%	10%
**Input Data: Top 40 Assets (**$x_{1}$ **to** $x_{40})$									
No.	k	Objective Function	$x_{5}$	$x_{8}$	$x_{9}$	$x_{16}$	$x_{27}$	$x_{29}$	$x_{38}$
			A_3_ (TRX)	A_15_ (UNI)	A_17_ (CRO)	A_18_ (ONDO)	A_40_ (CELO)	A_46_ (XTZ)	A_14_ (CORE)
Scenario 1	k=3	-0.070	50%	10%	40%	–	–	–	–
Scenario 2	k=5	-0.057	50%	10%	20%	10%	–	–	10%
Scenario 3	k=7	-0.041	40%	10%	10%	10%	10%	10%	10%
**Input Data: All Assets (**$x_{1}$ **to** $x_{47})$									
No.	k	Objective Function	$x_{5}$	$x_{8}$	$x_{9}$	$x_{16}$	$x_{38}$	$x_{44}$	$x_{46}$
			A_3_ (TRX)	A_15_ (UNI)	A_17_ (CRO)	A_18_ (ONDO)	A_14_ (CORE)	A_16_ (MNT)	A_19_ (NUM)
Scenario 1	k=3	-0.105	40%	–	10%	–	–	–	50%
Scenario 2	k=5	-0.094	20%	10%	10%	–	10%	–	50%
Scenario 3	k=7	-0.074	10%	10%	10%	10%	10%	10%	40%

Source: Authors’ own computation

As observed in the results, assets A_3_ (TRX, rank 5) and A_15_ (CRO, rank 9) were included in all portfolio constructions, regardless of the input data configurations and scenarios. In addition, assets UNI (rank 8) and A_17_ were present in all scenarios and input data sets, with the exception of the portfolio that utilized 47 input data points and a cardinality of k=3. Notably, all these assets belong to the top 10 category, classified as excellent assets. This consistent presence across various portfolios further emphasizes the reliability and quality of these selections in optimizing investment outcomes.

As reflected in the results, the model successfully constructed robust portfolios across different input data configurations. It effectively generated various scenarios tailored for different types of investors. These outcomes underscore the efficiency of our proposed model, demonstrating its capability to adapt to diverse requirements. Notably, the results show that portfolios formed from high-quality assets outperform those generated from other datasets. This finding highlights the critical importance of the preselection process prior to portfolio optimization, as selecting superior assets enhances overall portfolio performance. The analysis confirms that thoughtful asset selection is vital for achieving optimal investment outcomes.

## 5. Discussion and Practical implications

This paper introduces a two-stage framework designed to enhance cryptocurrency portfolio performance, addressing a critical gap in the literature and providing practical tools for investors.

The first stage emphasizes the pre-selection of high-potential assets through an innovative approach grounded in MADM methods. This strategy enables investors to systematically identify and evaluate assets that exhibit promising characteristics, significantly enhancing the asset selection process. Given the inherent volatility and unpredictability of the cryptocurrency market, the emphasis on high-quality pre-selection highlights the crucial need for rigorous asset evaluation. Historically, the lack of comprehensive studies addressing asset pre-selection in cryptocurrency portfolio optimization has resulted in the absence of established performance criteria specifically tailored for evaluating cryptocurrencies. This paper addresses this critical gap by introducing comprehensive criteria for the pre-selection of cryptocurrency assets. By providing a structured methodology, the research empowers investors to cherry-pick high-quality assets based on systematic evaluations rather than relying on intuition or superficial analysis. From a methodological perspective, this paper also contributes a robust framework for ranking and pre-selecting crypto-assets grounded in MADM techniques. One of the notable challenges associated with MADM approaches is that different methods can yield varying results, creating uncertainty regarding which technique provides the most reliable outcomes. To combat this issue, this paper presents a unified framework for pre-selection that synthesizes results from multiple MADM methods. By combining insights from various approaches, the framework enhances reliability and fosters greater trust in the asset selection process. Moreover, the implications of this framework extend beyond cryptocurrency. Its methodologies can be applied in various fields within decision science and engineering, where the ranking and ordering of alternatives are essential. This versatility underscores the framework’s potential to inform asset selection processes across different asset classes and investment contexts, ultimately leading to more effective decision-making. Therefore, the first stage of this two-stage framework not only fills a significant gap in the literature but also provides practical tools and methodologies that empower investors to make informed decisions in the rapidly evolving cryptocurrency landscape.

In the second stage, the focus shifts to the optimization process itself. Recognizing that uncertainty is a fundamental characteristic of capital markets, the framework incorporates credibility theory within the CVaR framework. By integrating these methodologies, the model effectively leverages their combined strengths to address downside risk and manage uncertainty during the optimization phase. This dual approach not only enhances the robustness of the portfolio but also empowers investors to make informed decisions in a volatile market landscape. Integrating credibility theory with traditional risk management frameworks equips managers with the tools necessary to navigate the complexities of cryptocurrency investments. This framework enables decision-makers to assess and account for uncertainties, leading to more resilient investment strategies. The ability to model downside risk effectively aids in capital preservation, particularly in a market characterized by rapid fluctuations and unpredictable events.

Therefore, the two-stage framework presented in this paper offers a structured and comprehensive pathway for enhancing cryptocurrency portfolio performance. By focusing on high-quality pre-selection in the first stage and sophisticated optimization in the second, this framework provides invaluable insights for investors seeking to navigate the intricacies of the cryptocurrency market. This holistic approach not only aids in optimizing investment strategies but also prepares investors to respond effectively to the challenges posed by market volatility and uncertainty. Furthermore, the methodologies introduced in this paper have broad applicability beyond cryptocurrency, providing valuable insights for asset selection processes across various investment domains. The principles of the proposed two-stage framework can be seamlessly applied to traditional financial markets, such as stocks, bonds, and alternative investments, thereby enhancing the asset selection process and optimizing portfolio construction in these markets as well. By integrating systematic pre-selection with advanced optimization techniques, this framework equips investors and managers with powerful tools for better decision-making, ensuring that portfolios are aligned with both risk and return objectives.

While the two-stage framework presented in this paper offers a structured and innovative approach to cryptocurrency portfolio optimization, several limitations should be acknowledged. First, the methodology relies on the availability of high-quality and consistent data, which can be a significant challenge in the cryptocurrency market due to its rapid evolution and volatility. Data gaps, especially in newly launched cryptocurrencies, may affect the accuracy of the asset pre-selection process. Additionally, while the integration of credibility theory with CVaR improves risk management, it may not account for all potential market dynamics, particularly during extreme market events. Furthermore, the framework also assumes that investors have access to sufficient computational resources and expertise to implement the methodologies effectively, which may limit its applicability to some investors. Lastly, while the proposed methods have been demonstrated in the context of cryptocurrency, further research is needed to test their effectiveness across other asset classes and market conditions. These limitations suggest that while the framework provides valuable insights, its real-world application may require adaptation to specific contexts and additional refinements.

## 6. Conclusions

This study presents a comprehensive two-stage framework aimed at enhancing cryptocurrency portfolio performance, specifically designed to tackle the challenges of portfolio construction in volatile and uncertain cryptocurrency markets. The framework comprises two sequential stages: Stage 1 focuses on the pre-selection of high-potential assets using a novel asset pre-selection approach. This process begins with the evaluation of 47 cryptocurrency assets, which are sorted based on comprehensive performance criteria. The assets are then categorized into five distinct groups, allowing for a systematic assessment. From these groups, we identify the top-performing segment, selecting the top 10 assets that exhibit the highest potential for returns. Stage 2 optimizes the selected assets through the application of a credibilistic CVaR model, while also considering various cardinality constraints to construct different scenarios for portfolio allocation. The effectiveness of this two-stage framework was rigorously tested by comparing the resultant portfolios against those constructed from the other asset groups. The results of this two-stage framework demonstrate its effectiveness in constructing well-diversified and efficient portfolios, addressing both the challenges of asset pre-selection and the complexities associated with uncertainty. By integrating these methodologies, investors are better equipped to navigate the risks associated with cryptocurrency investments while maximizing potential returns. This innovative approach not only enhances portfolio performance but also provides valuable insights for investors operating in the dynamic landscape of cryptocurrency markets.

Although the proposed framework represents a significant advancement in enhancing cryptocurrency portfolio performance, several avenues for further research remain. One promising area is the development of robust performance criteria that can be applied to other markets, such as equities, bonds, and commodities. This would facilitate the implementation of the pre-selection process beyond cryptocurrencies, enabling a broader application of the framework. Additionally, exploring alternative fuzzy number representations, such as coherent fuzzy numbers, could provide a more nuanced understanding of investor expectations. Traditional fuzzy numbers may not adequately represent the complexities of investor sentiment, especially in volatile markets. By investigating how these alternative representations influence portfolio performance and risk management, researchers could uncover new insights that enhance decision-making processes. Furthermore, the reliability of expert opinions in gathering fuzzy data is another critical aspect to consider. Expert insights are often essential in shaping investment decisions, particularly in uncertain environments like cryptocurrency markets. However, the subjective nature of these opinions can introduce bias. By utilizing Z-number theory, researchers could develop a more structured framework for incorporating expert insights, mitigating potential biases while enhancing the credibility of the data. This approach could result in more accurate models that reflect true market conditions, ultimately improving portfolio outcomes.

It is crucial to acknowledge that digital assets, especially cryptocurrencies, are continually evolving and are marked by substantial volatility and inherent risks. The value of most cryptocurrencies is largely influenced by speculative trading, where market sentiment is a key driver. Therefore, investors must exercise caution and perform comprehensive due diligence before making investment decisions in the cryptocurrency market, fully understanding its speculative nature and the various factors that can impact market dynamics.

## Supporting information

S1 Appendix Appendix S1: Pseudo Code of Selected MADM Algorithms and Required Further Calculations. (DOCX)
