# Supplementary material for: A two-stage framework for enhancing crsyptocurrency portfolio performance: Integrating credibilistic CVaR criterion with a novel asset preselection approach
Source: PLoS One. 2025 Jul 21;20(7):e0325973. doi: 10.1371/journal.pone.0325973 (PMC12279154; doi:10.1371/journal.pone.0325973)
Supplement: S1 Appendix — (DOCX) [file pone.0325973.s001.docx]

**Appendix 1: MARCOS (Measurement of Alternatives and Ranking according to COmpromise Solution)**

***Inputs:***

- $D_{ij}$: Decision matrix $\left( m\times n \right)$ where $i = 1, 2, \ldots, m$ (Alternatives) and $j=1,2,\ldots,n$ (criteria).
- $W_{j}$: Weight vector for the criteria $\left( \sum_{j=1}^{n} W_{j}=1 \right)$.
- $T_{j}$: Criterion type, where $Tj\in\{Benefit, Cost$}.

***Outputs:***

- $R[i]$: Ranking of alternatives.
- $CS[i]$: Compromise solution coefficients for all alternatives.

***Step 1:*** *Normalize the Decision Matrix*

Normalize the decision matrix based on the type of each criterion $\left( T_{j} \right)$.

- If $Tj=Benefit: N_{ij}=\frac{D_{ij}}{\max\left( D_{kj} \right)},\quad\forall k\in\{1,2,\ldots,m\}$
- If $Tj=Cost: N_{ij}=\frac{\min\left( D_{kj} \right)}{D_{ij}},\quad\forall k\in\{1,2,\ldots,m\}$

***Step 2:*** *Construct the Ideal and Anti-Ideal Solutions*

Compute the ideal $\left( AI_{j} \right)$ and anti-ideal $\left( AAI_{j} \right)$ values for each criterion.

$$AI_{j}=\max\left( N_{ij} \right),\quad\forall i\in\{1,2,\ldots,m\}$$

$$AAI_{j}=\min\left( N_{ij} \right),\quad\forall i\in\{1,2,\ldots,m\}$$

***Step 3:*** *Compute Utility Degrees*

Compute the utility degree $\left( U\left[ i \right] \right)$ for each alternative $i$, and for the ideal $\left( U_{AI} \right)$ and anti-ideal $\left( U_{AAI} \right)$ solutions.

- For alternatives:

$$U\left[ i \right]=\sum_{j=1}^{n} W_{j}\cdot N_{ij},\quad\forall i\in\{1,2,\ldots,m\}$$

- For the ideal solution:

$$U_{AI}=\sum_{j=1}^{n} W_{j}\cdot AI_{j}$$

- For the anti-ideal solution:

$$U_{AAI}=\sum_{j=1}^{n} W_{j}\cdot AAI_{j}$$

***Step 4:*** *Calculate Utility Coefficients*

Determine the utility coefficient $\left( K\left[ i \right] \right)$ for each alternative.

$$K\left[ i \right]=\frac{U\left[ i \right]}{U_{AAI}},\quad\forall i\in\{1,2,\ldots,m\}$$

***Step 5:*** *Compute Compromise Solution Coefficients*

Calculate the compromise solution coefficient $(CS[i])$ for each alternative.

$$CS\left[ i \right]=\frac{K\left[ i \right]-1}{\frac{U_{AI}}{U_{AAI}}-1},\quad\forall i\in\{1,2,\ldots,m\}$$

***Step 6:*** *Rank Alternatives*

Rank the alternatives based on the descending order of $CS[i]$.

**Appendix 2: CODAS (Combinative Distance-based Assessment)**

***Inputs:***

- $D_{ij}$: Decision matrix $\left( m\times n \right)$ where $i = 1, 2, \ldots, m$ (Alternatives) and $j=1,2,\ldots,n$ (criteria).
- $W_{j}$: Weight vector for the criteria $\left( \sum_{j=1}^{n} W_{j}=1 \right)$.
- $T_{j}$: Criterion type, where $Tj\in\{Benefit, Cost$}.
- $\tau:$ Threshold parameter for $TEDAST_{EDAS}\left( e.g.,\tau=0.02 \right)$.

***Outputs:***

- $R[i]:$ Ranking of alternatives.
- $AS[i]:$ Assessment scores for all alternatives.

***Step 1****: Normalize the Decision Matrix*

Normalize the decision matrix based on the type of each criterion $\left( T_{j} \right)$.

- *If* $Tj=Benefit: N_{ij}=\frac{D_{ij}}{\max\left( D_{kj} \right)},\quad\forall k\in\{1,2,\ldots,m\}$
- *If* $Tj=Cost: N_{ij}=\frac{\min\left( D_{kj} \right)}{D_{ij}},\quad\forall k\in\{1,2,\ldots,m\}$

*Step 2: Construct the Weighted Normalized Decision Matrix*

Compute the weighted normalized values $\left( WN_{ij} \right)$:

$$WN_{ij}=W_{j}\cdot N_{ij},\quad\forall i\in\{1,2,\ldots,m\},\forall j\in\{1,2,\ldots,n\}$$

*Step 3: Identify the Negative-Ideal Solution*

Determine the negative-ideal solution $\left( NIS_{j} \right)$:

$$NIS_{j}=\min\left( WN_{ij} \right),\quad\forall j\in\{1,2,\ldots,n\}$$

*Step 4: Compute the Euclidean and Taxicab Distances*

Calculate the Euclidean distance $\left( d_{E_{i}} \right)$ and Taxicab (Manhattan) distance $\left( d_{T_{i}} \right)$ of each alternative from the negative-ideal solution:

- Euclidean Distance:

$$d_{E_{i}}=\sqrt{\sum_{j=1}^{n} \left( WN_{ij}-NIS_{j} \right)^{2}},\quad\forall i\in\{1,2,\ldots,m\}$$

- Taxicab Distance:

$$d_{T_{i}}=\sum_{j=1}^{n} \left| WN_{ij}-NIS_{j} \right|,\quad\forall i\in\{1,2,\ldots,m\}$$

***Step 5****: Compute the Assessment Score*

Calculate the assessment score $(AS[i])$ for each alternative using the distances:

$$AS\left[ i \right]=d_{E_{i}}+\tau\cdot d_{T_{i}},\quad\forall i\in\{1,2,\ldots,m\}$$

*Step 6: Rank Alternatives*

Rank the alternatives based on descending order of $AS[i]$.

**Appendix 3: CoCoSo (COmbined COmpromise SOlution)**

***Inputs:***

- $D_{ij}$: Decision matrix $\left( m\times n \right)$ where $i = 1, 2, \ldots, m$ (Alternatives) and $j=1,2,\ldots,n$(criteria).
- $W_{j}$: Weight vector for the criteria $\left( \sum_{j=1}^{n} W_{j}=1 \right)$.
- $T_{j}$: Criterion type, where $Tj\in\{Benefit, Cost$}.
- Parameters $\lambda$ and $\mu$: CoCoSo adjustment parameters (default values: $\lambda=0.5, \mu=1$).

***Outputs:***

- $R[i]$: Ranking of alternatives.
- $S\left[ i \right]$: Combined compromise scores for all alternatives.

***Step 1:*** *Normalize the Decision Matrix*

Normalize the decision matrix based on the type of each criterion ($T_{j}$).

- *If* $Tj=Benefit: N_{ij}=\frac{D_{ij}}{\max\left( D_{kj} \right)},\quad\forall k\in\{1,2,\ldots,m\}$
- *If* $Tj=Cost: N_{ij}=\frac{\min\left( D_{kj} \right)}{D_{ij}},\quad\forall k\in\{1,2,\ldots,m\}$

***Step 2:*** *Compute the Weighted Normalized Matrix*

Calculate the weighted normalized values $\left( WN_{ij} \right)$:

$$WN_{ij}=W_{j}\cdot N_{ij},\quad\forall i\in\{1,2,\ldots,m\},\forall j\in\{1,2,\ldots,n\}$$

***Step 3:*** *Calculate Individual Scores for Each Alternative*

Compute three scores for each alternative:

- Simple Additive Weighting (SAW) Score:

$$S_{SAW}\left[ i \right]=\sum_{j=1}^{n} WN_{ij},\quad\forall i\in\{1,2,\ldots,m\}$$

- Power Weighting Product (PWP) Score:

$$S_{PWP}\left[ i \right]=\prod_{j=1}^{n} WN_{ij}^{W_{j}},\quad\forall i\in\{1,2,\ldots,m\}$$

- Overall Utility Score:

$$S_{OU}\left[ i \right]=\lambda\cdot S_{SAW}\left[ i \right]+\left( 1-\lambda\right)\cdot S_{PWP}\left[ i \right],\quad\forall i\in\{1,2,\ldots,m\}$$

***Step 4:*** *Adjust the Utility Scores*

Compute the adjusted scores ($AS\left[ i \right]$) for each alternative:

$$AS\left[ i \right]=\mu\cdot\left( \frac{S_{SAW}\left[ i \right]}{\max\left( S_{SAW} \right)} \right)+\left( 1-\mu\right)\cdot\left( \frac{S_{PWP}\left[ i \right]}{\max\left( S_{PWP} \right)} \right),\quad\forall i\in\{1,2,\ldots,m\}$$

***Step 5:*** *Combine the Scores*

Calculate the Combined Compromise Score $(S[i])$ for each alternative:

$$S\left[ i \right]=\frac{AS\left[ i \right]}{\sum_{k=1}^{m} AS\left[ k \right]},\quad\forall i\in\{1,2,\ldots,m\}$$

***Step 6:*** *Rank Alternatives*

Rank the alternatives based on descending order of $S\left[ i \right]$.

**Appendix 4: EDAS (Evaluation based on Distance from Average Solution)**

***Inputs:***

- $D_{ij}$: Decision matrix $\left( m\times n \right)$ where $i = 1, 2, \ldots, m$ (Alternatives) and $j=1,2,\ldots,n$ (criteria).
- $W_{j}$: Weight vector for the criteria $\left( \sum_{j=1}^{n} W_{j}=1 \right)$.
- $T_{j}$: Criterion type, where $Tj\in\{Benefit, Cost$}.

***Outputs:***

- $R[i]:$ Ranking of alternatives.
- $AS[i]:$ Appraisal scores for all alternatives.

***Step 1****: Compute the Average Solution*

Calculate the average solution ($AV_{j}$) for each criterion $j$:

$$AV_{j}=\frac{\sum_{i=1}^{m} D_{ij}}{m},\quad\forall j\in\{1,2,\ldots,n\}$$

***Step 2****: Calculate Positive and Negative Distances*

Compute the positive distance $\left( PD_{ij} \right)$ and negative distance $\left( ND_{ij} \right)$ for each alternative ii and criterion $j$:

- If $T_{j}=\text{Benefit}:$

$$PD_{ij}=\max\left( 0,D_{ij}-AV_{j} \right),\quad ND_{ij}=\max\left( 0,AV_{j}-D_{ij} \right)$$

- If$T_{j}=\text{Cost}:$

$$PD_{ij}=\max\left( 0,AV_{j}-D_{ij} \right),\quad ND_{ij}=\max\left( 0,D_{ij}-AV_{j} \right)$$

***Step 3****: Normalize the Positive and Negative Distances*

Normalize $PD_{ij}$ and $ND_{ij}$ across all alternatives:

$$NPD_{ij}=\frac{PD_{ij}}{\max\left( PD_{kj} \right)},\quad\forall k\in\{1,2,\ldots,m\}$$

$$NND_{ij}=\frac{ND_{ij}}{\max\left( ND_{kj} \right)},\quad\forall k\in\{1,2,\ldots,m\}$$

***Step 4****: Compute Weighted Aggregated Scores*

Calculate the weighted positive distance score $(SP[i])$ and the weighted negative distance score $(SN[i])$ for each alternative $i$:

$$SP\left[ i \right]=\sum_{j=1}^{n} W_{j}\cdot NPD_{ij}$$

$$SN\left[ i \right]=\sum_{j=1}^{n} W_{j}\cdot NND_{ij}$$

***Step 5****: Calculate the Appraisal Score*

Compute the appraisal score $(AS[i])$ for each alternative $i$:

$$AS\left[ i \right]=\frac{SP\left[ i \right]+\left( 1-SN\left[ i \right] \right)}{2}$$

***Step 6****: Rank Alternatives*

Rank the alternatives based on descending order of $AS\left[ i \right]$.

**Appendix 5:** **WASPAS (Weighted Aggregates Sum Product Assessment)**

***Inputs:***

- $D_{ij}$: Decision matrix $\left( m\times n \right)$ where $i = 1, 2, \ldots, m$ (Alternatives) and $j=1,2,\ldots,n$ (criteria).
- $W_{j}$: Weight vector for the criteria $\left( \sum_{j=1}^{n} W_{j}=1 \right)$.
- $T_{j}$: Criterion type, where $Tj\in\{Benefit, Cost$}.
- $\lambda$: WASPAS adjustment parameter ($0\leq\lambda\leq10 \leq\lambda\leq1, default is \lambda=0.5$).

***Outputs:***

- $R[i]:$ Ranking of alternatives.
- $Q[i]$: WASPAS scores for all alternatives.

***Step 1****: Normalize the Decision Matrix*

Normalize the decision matrix based on the type of each criterion ($Tj$).

- *If* $Tj=Benefit: N_{ij}=\frac{D_{ij}}{\max\left( D_{kj} \right)},\quad\forall k\in\{1,2,\ldots,m\}$
- *If* $Tj=Cost: N_{ij}=\frac{\min\left( D_{kj} \right)}{D_{ij}},\quad\forall k\in\{1,2,\ldots,m\}$

***Step 2****: Compute the Weighted Normalized Decision Matrix*

Calculate the weighted normalized values $\left( WN_{ij} \right):$

$$WN_{ij}=W_{j}\cdot N_{ij},\quad\forall i\in\{1,2,\ldots,m\},\forall j\in\{1,2,\ldots,n\}$$

***Step 3****: Compute the Sum Model (WSM) Score*

Calculate the Weighted Sum Model (WSM) score ($S[i]$) for each alternative $i$:

$$S\left[ i \right]=\sum_{j=1}^{n} WN_{ij},\quad\forall i\in\{1,2,\ldots,m\}$$

***Step 4****: Compute the Product Model (WPM) Score*

Calculate the Weighted Product Model (WPM) score ($P[i]$) for each alternative $i$:

$$P\left[ i \right]=\prod_{j=1}^{n} \left( N_{ij} \right)^{W_{j}},\quad\forall i\in\{1,2,\ldots,m\}$$

***Step 5****: Calculate the WASPAS Score*

Combine $S[i]$ and $P[i]$ using the parameter λ\lambda to calculate the WASPAS score ($Q[i]$) for each alternative $i$:

$$Q\left[ i \right]=\lambda\cdot S\left[ i \right]+\left( 1-\lambda\right)\cdot P\left[ i \right],\quad\forall i\in\{1,2,\ldots,m\}$$

***Step 6****: Rank Alternatives*

Rank the alternatives based on descending order of $Q[i]$.

**Appendix 6: TOPSIS (Technique for Order of Preference by Similarity to Ideal Solution)**

***Inputs:***

- $D_{ij}$: Decision matrix $\left( m\times n \right)$ where $i = 1, 2, \ldots, m$ (Alternatives) and $j=1,2,\ldots,n$ (criteria).
- $W_{j}$: Weight vector for the criteria $\left( \sum_{j=1}^{n} W_{j}=1 \right)$.
- $T_{j}$: Criterion type, where $Tj\in\{Benefit, Cost$}.

***Outputs:***

- $R[i]$: Ranking of alternatives.
- $C[i]$: Closeness coefficient for all alternatives.

***Step 1****: Normalize the Decision Matrix*

Normalize the decision matrix using the Euclidean norm:

$$N_{ij}=\frac{D_{ij}}{\sqrt{\sum_{k=1}^{m} D_{kj}^{2}}},\quad\forall i\in\{1,2,\ldots,m\},\forall j\in\{1,2,\ldots,n\}$$

***Step 2****: Compute the Weighted Normalized Decision Matrix*

Calculate the weighted normalized values $\left( WN_{ij} \right)$:

$$WN_{ij}=W_{j}\cdot N_{ij},\quad\forall i\in\{1,2,\ldots,m\},\forall j\in\{1,2,\ldots,n\}$$

***Step 3****: Determine the Ideal and Negative-Ideal Solutions*

Compute the ideal $\left( A+A^{+} \right)$ and negative-ideal $\left( A-A^{-} \right)$ solutions:

- For benefit criteria:

$$A_{j}^{+}=\max\left( WN_{ij} \right),\quad A_{j}^{-}=\min\left( WN_{ij} \right),\quad\forall j\in\{1,2,\ldots,n\}$$

- For cost criteria:

$$A_{j}^{+}=\min\left( WN_{ij} \right),\quad A_{j}^{-}=\max\left( WN_{ij} \right),\quad\forall j\in\{1,2,\ldots,n\}$$

***Step 4****: Calculate the Separation Measures*

Compute the separation measures for each alternative:

- Separation from the Ideal Solution ($Si+S_{i}^{+}$):

$$S_{i}^{+}=\sqrt{\sum_{j=1}^{n} \left( WN_{ij}-A_{j}^{+} \right)^{2}},\quad\forall i\in\{1,2,\ldots,m\}$$

- Separation from the Negative-Ideal Solution ($Si-S_{i}^{-}$):

$$S_{i}^{-}=\sqrt{\sum_{j=1}^{n} \left( WN_{ij}-A_{j}^{-} \right)^{2}},\quad\forall i\in\{1,2,\ldots,m\}$$

***Step 5****: Compute the Closeness Coefficient*

Calculate the closeness coefficient ($C\left[ i \right]$) for each alternative:

$$C\left[ i \right]=\frac{S_{i}^{-}}{S_{i}^{+}+S_{i}^{-}},\quad\forall i\in\{1,2,\ldots,m\}$$

***Step 6****: Rank Alternatives*

Rank the alternatives based on descending order of $C\left[ i \right]$.

**Appendix 7: MOORA (Multi-Objective Optimization on the basis of Ratio Analysis)**

***Inputs:***

- $D_{ij}$: Decision matrix $\left( m\times n \right)$ where $i = 1, 2, \ldots, m$ (Alternatives) and $j=1,2,\ldots,n$ (criteria).
- $W_{j}$: Weight vector for the criteria $\left( \sum_{j=1}^{n} W_{j}=1 \right)$.
- $T_{j}$: Criterion type, where $Tj\in\{Benefit, Cost$}.

***Outputs:***

- $R[i]$: Ranking of alternatives.
- $Q[i]$: MOORA scores for all alternatives.

***Step 1****: Normalize the Decision Matrix*

Normalize the decision matrix using the vector normalization method:

$$N_{ij}=\frac{D_{ij}}{\sqrt{\sum_{k=1}^{m} D_{kj}^{2}}},\quad\forall i\in\{1,2,\ldots,m\},\forall j\in\{1,2,\ldots,n\}$$

***Step 2****: Compute the Weighted Normalized Decision Matrix*

Calculate the weighted normalized values $\left( WN_{ij} \right)$:

$$WN_{ij}=W_{j}\cdot N_{ij},\quad\forall i\in\{1,2,\ldots,m\},\forall j\in\{1,2,\ldots,n\}$$

***Step 3****: Calculate the MOORA Scores*

Compute the MOORA score ($Q[i]$) for each alternative by separating benefit and cost criteria:

- Benefit Criteria Contribution:

$$B\left[ i \right]=\sum_{j\in\text{Benefit}} WN_{ij},\quad\forall i\in\{1,2,\ldots,m\}$$

- Cost Criteria Contribution:

$$C\left[ i \right]=\sum_{j\in\text{Cost}} WN_{ij},\quad\forall i\in\{1,2,\ldots,m\}$$

- Overall MOORA Score:

$$Q\left[ i \right]=B\left[ i \right]-C\left[ i \right],\quad\forall i\in\{1,2,\ldots,m\}$$

***Step 4****: Rank Alternatives*

Rank the alternatives based on descending order of $Q[i]$.

**Appendix 8: COPRAS (Complex PRoportional Assessment)**

***Inputs:***

- $D_{ij}$: Decision matrix $\left( m\times n \right)$ where $i = 1, 2, \ldots, m$ (Alternatives) and $j=1,2,\ldots,n$ (criteria).
- $W_{j}$: Weight vector for the criteria $\left( \sum_{j=1}^{n} W_{j}=1 \right)$.
- $T_{j}$: Criterion type, where $Tj\in\{Benefit, Cost$}.

***Outputs:***

- $R[i]:$ Ranking of alternatives.
- $S[i]$: Relative significance score for all alternatives.

***Step 1****: Normalize the Decision Matrix*

Normalize the decision matrix for all ii and j:

$$N_{ij}=\frac{D_{ij}}{\sum_{k=1}^{m} D_{kj}},\quad\forall i\in\{1,2,\ldots,m\},\forall j\in\{1,2,\ldots,n\}$$

***Step 2****: Compute the Weighted Normalized Decision Matrix*

Calculate the weighted normalized values $\left( WN_{ij} \right)$:

$$WN_{ij}=W_{j}\cdot N_{ij},\quad\forall i\in\{1,2,\ldots,m\},\forall j\in\{1,2,\ldots,n\}$$

***Step 3****: Separate Benefit and Cost Contributions*

Separate the benefit and cost contributions for each alternative i:

- Sum of Benefit Contributions:

$$S_{B}\left[ i \right]=\sum_{j\in\text{Benefit}} WN_{ij},\quad\forall i\in\{1,2,\ldots,m\}$$

- Sum of Cost Contributions**:**

$$S_{C}\left[ i \right]=\sum_{j\in\text{Cost}} WN_{ij},\quad\forall i\in\{1,2,\ldots,m\}$$

***Step 4****: Compute the Utility Degree for Each Alternative*

Calculate the relative significance score ($S[i]$) for each alternative i:

$$S\left[ i \right]=S_{B}\left[ i \right]+\frac{\min\left( S_{C} \right)}{S_{C}\left[ i \right]\cdot\sum_{k=1}^{m} \frac{\min\left( S_{C} \right)}{S_{C}\left[ k \right]}},\quad\forall i\in\{1,2,\ldots,m\}$$

***Step 5****: Rank Alternatives*

Rank the alternatives based on descending order of $S[i]$.

**Appendix 9: ARAS (Additive Ratio ASsessment)**

***Inputs:***

- $D_{ij}$: Decision matrix $\left( m\times n \right)$ where $i = 1, 2, \ldots, m$ (Alternatives) and $j=1,2,\ldots,n$ (criteria).
- $W_{j}$: Weight vector for the criteria $\left( \sum_{j=1}^{n} W_{j}=1 \right)$.
- $T_{j}$: Criterion type, where $Tj\in\{Benefit, Cost$}.

***Outputs:***

- $R[i]:$ Ranking of alternatives.
- $U[i]$: Utility scores for all alternatives.

***Step 1****: Normalize the Decision Matrix*

Normalize the decision matrix $\left( N_{ij} \right)$ for all ii and j:

- If $T_{j}=\text{Benefit}$:

$$N_{ij}=\frac{D_{ij}}{\sum_{k=1}^{m} D_{kj}},\quad\forall k\in\{1,2,\ldots,m\}$$

- If $T_{j}=\text{Cost}$:

$$N_{ij}=\frac{\sum_{k=1}^{m} D_{kj}}{D_{ij}},\quad\forall k\in\{1,2,\ldots,m\}$$

***Step 2****: Compute the Weighted Normalized Decision Matrix*

Calculate the weighted normalized values $\left( WN_{ij} \right)$:

$$WN_{ij}=W_{j}\cdot N_{ij},\quad\forall i\in\{1,2,\ldots,m\},\forall j\in\{1,2,\ldots,n\}$$

***Step 3****: Calculate the Overall Performance Score*

Compute the overall performance score ($S[i]$) for each alternative $i$:

$$S\left[ i \right]=\sum_{j=1}^{n} WN_{ij},\quad\forall i\in\{1,2,\ldots,m\}$$

***Step 4****: Calculate the Utility Degree*

Calculate the utility degree ($U[i]$) of each alternative relative to the best-performing alternative:

$$U\left[ i \right]=\frac{S\left[ i \right]}{S_{0}},\quad S_{0}=\max\left( S\left[ i \right] \right),\quad\forall i\in\{1,2,\ldots,m\}$$

***Step 5****: Rank Alternatives*

Rank the alternatives based on descending order of $U[i]$.

**Appendix 10: VIKOR (VIseKriterijumska Optimizacija I Kompromisno Resenje)**

***Inputs:***

- $D_{ij}$: Decision matrix $\left( m\times n \right)$ where $i = 1, 2, \ldots, m$ (Alternatives) and $j=1,2,\ldots,n$ (criteria).
- $W_{j}$: Weight vector for the criteria $\left( \sum_{j=1}^{n} W_{j}=1 \right)$.
- $T_{j}$: Criterion type, where $Tj\in\{Benefit, Cost$}.
- $v$: Weight for the decision strategy $\left( e.g.,v=0.5 \right).$

***Outputs:***

- $R[i]:$ Ranking of alternatives.
- $Q[i]$: Compromise solution scores for all alternatives.

***Step 1****: Determine the Ideal and Negative-Ideal Solutions*

Identify the ideal ($fj+f_{j}^{+}$) and negative-ideal ($fj-f_{j}^{-}$) values for each criterion:

- *For benefit criteria:*

$$f_{j}^{+}=\max\left( D_{ij} \right),\quad f_{j}^{-}=\min\left( D_{ij} \right),\quad\forall j\in\{1,2,\ldots,n\}$$

- *For cost criteria:*

$$f_{j}^{+}=\min\left( D_{ij} \right),\quad f_{j}^{-}=\max\left( D_{ij} \right),\quad\forall j\in\{1,2,\ldots,n\}$$

***Step 2:*** *Calculate the Weighted Normalized Distances*

Compute the weighted normalized distance from the ideal and negative-ideal values:

$$S_{i}=\sum_{j=1}^{n} W_{j}\cdot\frac{\left| f_{j}^{+}-D_{ij} \right|}{\left| f_{j}^{+}-f_{j}^{-} \right|},\quad\forall i\in\{1,2,\ldots,m\}$$

$$R_{i}=\max_{j\in\{1,2,\ldots,n\}} \left[ W_{j}\cdot\frac{\left| f_{j}^{+}-D_{ij} \right|}{\left| f_{j}^{+}-f_{j}^{-} \right|} \right],\quad\forall i\in\{1,2,\ldots,m\}$$

***Step 3****: Compute the VIKOR Index*

Calculate the compromise solution score ($Q[i]$) for each alternative:

$$\left[ i \right]=v\cdot\frac{S_{i}-S^{+}}{S^{-}-S^{+}}+\left( 1-v \right)\cdot\frac{R_{i}-R^{+}}{R^{-}-R^{+}},\quad\forall i\in\{1,2,\ldots,m\}$$

Where:

- $S^{+}=\min\left( S_{i} \right),S^{-}=\max\left( S_{i} \right),$
- $R^{+}=\min\left( R_{i} \right),R^{-}=\max\left( R_{i} \right).$

***Step 4****: Rank Alternatives*

Rank the alternatives based on ascending order of $Q[i]$. The smallest $Q\left[ i \right]$ represents the best alternative.

**Appendix 11: MABAC (Multi-Attributive Border Approximation area Comparison)**

***Inputs:***

- $D_{ij}$: Decision matrix $\left( m\times n \right)$ where $i = 1, 2, \ldots, m$ (Alternatives) and $j=1,2,\ldots,n$ (criteria).
- $W_{j}$: Weight vector for the criteria $\left( \sum_{j=1}^{n} W_{j}=1 \right)$.
- $T_{j}$: Criterion type, where $Tj\in\{Benefit, Cost$}.

***Outputs:***

- $R[i]:$ Ranking of alternatives.
- $Q[i]$: MABAC scores for all alternatives.

***Step 1****: Normalize the Decision Matrix*

Normalize the decision matrix based on the type of each criterion $\left( T_{j} \right)$:

- For benefit criteria:

$$N_{ij}=\frac{D_{ij}-\min\left( D_{kj} \right)}{\max\left( D_{kj} \right)-\min\left( D_{kj} \right)},\quad\forall k\in\{1,2,\ldots,m\}$$

- For cost criteria:

$$N_{ij}=\frac{\max\left( D_{kj} \right)-D_{ij}}{\max\left( D_{kj} \right)-\min\left( D_{kj} \right)},\quad\forall k\in\{1,2,\ldots,m\}$$

***Step 2****: Compute the Weighted Normalized Decision Matrix*

Calculate the weighted normalized values ($WN_{ij}$):

$$WN_{ij}=W_{j}\cdot N_{ij},\quad\forall i\in\{1,2,\ldots,m\},\forall j\in\{1,2,\ldots,n\}$$

***Step 3****: Define the Border Approximation Area (BAA)*

The border approximation area $\left( G_{j} \right)$ is computed as the average of the weighted normalized values across all alternatives for each criterion:

$$G_{j}=\frac{\sum_{i=1}^{m} WN_{ij}}{m},\quad\forall j\in\{1,2,\ldots,n\}$$

***Step 4****: Calculate the Distance to the Border Approximation Area*

Determine the distance of each alternative to the BAA for each criterion:

$$d_{ij}=WN_{ij}-G_{j},\quad\forall i\in\{1,2,\ldots,m\},\forall j\in\{1,2,\ldots,n\}$$

***Step 5****: Compute the Overall MABAC Score*

Aggregate the distances to calculate the overall MABAC score ($Q[i]$) for each alternative:

$$Q\left[ i \right]=\sum_{j=1}^{n} d_{ij},\quad\forall i\in\{1,2,\ldots,m\}$$

***Step 6****: Rank Alternatives*

Rank the alternatives based on descending order of $Q[i]$.

**Appendix 12: MACBETH (Measuring Attractiveness by a Categorical Based Evaluation TecHnique)**

***Inputs:***

- $A=\{A_{1},A_{2},\ldots,A_{m}\}:$ Set of mm alternatives.
- $C=\{C_{1},C_{2},\ldots,C_{n}$}: Set of nn criteria.
- $W_{j}$: Weight vector for criteria $\left( \sum_{j=1}^{n} W_{j}=1 \right)$.
- Pairwise attractiveness judgments for criteria and alternatives on a predefined qualitative scale (e.g., “no difference”, “weak”, “strong”, etc.).

***Outputs:***

- $R[i]$: Ranking of alternatives.
- $S[i]$: Overall scores for all alternatives.

***Step 1****: Construct the Pairwise Comparison Matrix for Criteria*

For each pair of criteria $\left( C_{i},C_{j} \right)$, determine their relative importance using the MACBETH qualitative scale. Populate the pairwise comparison matrix $MCM_{C}$, where:

$$M_{C}\left[ i,j \right]=\text{judged attractiveness of }C_{i}\text{ over }C_{j}.$$

***Step 2****: Convert the Qualitative Judgments into Quantitative Weights*

Using MACBETH’s interval consistency approach, convert the qualitative judgments in $MCM_{C}$ into a set of numerical weights $W_{j}$ for each criterion such that:

$$\sum_{j=1}^{n} W_{j}=1.$$

***Step 3****: Construct the Pairwise Comparison Matrix for Alternatives*

For each criterion $C_{j}$, evaluate the pairwise attractiveness of alternatives $\left( A_{i},A_{k} \right)$ based on their performance under $C_{j}$, using the same qualitative scale. Populate the matrix $M_{A}^{\left( j \right)}$ for each criterion.

***Step 4****: Convert Judgments for Alternatives into Performance Scores*

Using the MACBETH methodology, convert the qualitative judgments in $M_{A}^{\left( j \right)}$ into quantitative performance scores $S_{ij}$, where:

$$S_{ij}=\text{Performance score of }A_{i}\text{ under }C_{j}.$$

***Step 5****: Aggregate the Scores Across Criteria*

Compute the overall performance score $S\left[ i \right]$ for each alternative $A_{i}$ by aggregating the weighted scores across all criteria:

$$S\left[ i \right]=\sum_{j=1}^{n} W_{j}\cdot S_{ij},\quad\forall i\in\{1,2,\ldots,m\}.$$

***Step 6****: Rank Alternatives*

Rank the alternatives based on descending order of $S\left[ i \right]$.

**Appendix 13: TODIM (TOmada de Decisao Interativa e Multicriterio - Interactive and Multicriteria Decision Making)**

***Inputs:***

- $D_{ij}$: Decision matrix $\left( m\times n \right)$ where $i = 1, 2, \ldots, m$ (Alternatives) and $j=1,2,\ldots,n$ (criteria).
- $W_{j}$: Weight vector for the criteria $\left( \sum_{j=1}^{n} W_{j}=1 \right)$.
- $\theta$: Loss aversion coefficient (commonly $\theta>0$).

***Outputs:***

- $R\left[ i \right]$: Ranking of alternatives.
- $V\left[ i \right]$: Overall dominance scores for all alternatives.

***Step 1****: Normalize the Decision Matrix*

Normalize the decision matrix based on the type of each criterion ($T_{j}$):

- For benefit criteria:

$$N_{ij}=\frac{D_{ij}-\min\left( D_{kj} \right)}{\max\left( D_{kj} \right)-\min\left( D_{kj} \right)},\quad\forall k\in\{1,2,\ldots,m\}$$

- For cost criteria:

$$N_{ij}=\frac{\max\left( D_{kj} \right)-D_{ij}}{\max\left( D_{kj} \right)-\min\left( D_{kj} \right)},\quad\forall k\in\{1,2,\ldots,m\}$$

***Step 2****: Compute the Relative Dominance for Each Pair of Alternatives*

For each pair of alternatives $A_{i}$ and $A_{k}$, compute the dominance function $\Delta_{ik}^{j}$ for each criterion $C_{j}$:

- If $N_{ij}\geq N_{kj}$:

$$\Delta_{ik}^{j}=W_{j}\cdot\frac{N_{ij}-N_{kj}}{\max\left( N_{kj} \right)}$$

- If $N_{ij}<N_{kj}$:

$$\Delta_{ik}^{j}=-W_{j}\cdot\theta\cdot\frac{N_{kj}-N_{ij}}{\max\left( N_{kj} \right)}$$

***Step 3****: Aggregate the Dominance for All Criteria*

Calculate the overall dominance $\Delta_{ik}$ between each pair $\left( A_{i},A_{k} \right)$ by summing across all criteria:

$$\Delta_{ik}=\sum_{j=1}^{n} \Delta_{ik}^{j},\quad\forall i,k\in\{1,2,\ldots,m\}$$

***Step 4****: Compute the Global Dominance for Each Alternative*

For each alternative $A_{i}$, calculate its global dominance score $V\left[ i \right]$:

$$V\left[ i \right]=\sum_{k=1,k\neq i}^{m} \Delta_{ik},\quad\forall i\in\{1,2,\ldots,m\}$$

***Step 5****: Rank Alternatives*

Rank the alternatives based on descending order of $V\left[ i \right]$.

**Appendix 14:**

As we mentioned, the “Avg Active Addresses” data were not readily available and required further calculations. In this appendix, we provide a detailed breakdown of these calculations. For each asset, we collected data for four quarters, and the average of these values resulted in the final column. Here is the summarized data:

**Table A1.** Calculation of “Average Active Addresses” for each asset

| Alternative | | | Active Addresses (Million) | | | | |
| --- | --- | --- | --- | --- | --- | --- | --- |
|  |  |  | Q1 | Q2 | Q3 | Q4 | Average |
| A_1_ | ETH | Ethereum | 5.2 | 7.2 | 6.5 | 5.9 | 6.200 |
| A_2_ | SOL | Solana | 3.1 | 4.8 | 6.2 | 12.1 | 6.550 |
| A_3_ | TRX | Tron | 12.8 | 12.3 | 15.3 | 14.1 | 13.625 |
| A_4_ | BNB | BSC (Binance) | 12.4 | 13.2 | 9.9 | 11.2 | 11.675 |
| A_5_ | BTC | Bitcoin | 16.5 | 13.5 | 9.6 | 8.8 | 12.100 |
| A_6_ | LINK | Chainlink | 0.062 | 0.052 | 0.043 | 0.032 | 0.047 |
| A_7_ | ARB | Arbitrum | 2.5 | 4.5 | 5.7 | 3.2 | 3.975 |
| A_8_ | SUI | Sui | 0.02 | 0.045 | 0.064 | 0.025 | 0.039 |
| A_9_ | AVAX | Avalanche | 0.827 | 0.889 | 0.631 | 0.404 | 0.688 |
| A_10_ | POL | Polygon | 4.9 | 8.4 | 8.6 | 5 | 6.725 |
| A_11_ | CRV | Curve | 1.98 | 3.18 | 2.37 | 1.07 | 2.150 |
| A_12_ | APT | Aptos | 0.969 | 2.5 | 3.9 | 4.2 | 2.892 |
| A_13_ | OP | Optimism | 1.2 | 2.1 | 1.7 | 1.4 | 1.600 |
| A_14_ | CORE | CORE | 5.5 | 4.5 | 3 | 3.9 | 4.225 |
| A_15_ | UNI | Uniswap | 0.63 | 1.5 | 6.2 | 14.9 | 5.808 |
| A_16_ | MNT | Mantle | 0.018 | 0.019 | 0.038 | 0.024 | 0.025 |
| A_17_ | CRO | Cronos | 4.8 | 5.6 | 8.8 | 7.6 | 6.700 |
| A_18_ | ONDO | Ondo | 6.3 | 14.5 | 11.2 | 10.1 | 10.525 |
| A_19_ | NUM | Numbers | 0.001 | 0.003 | 0.004 | 0.005 | 0.003 |
| A_20_ | CAKE | Pancakeswap | 1.6 | 1.7 | 0.937 | 0.985 | 1.306 |
| A_21_ | MKR | Maker | 0.28 | 0.301 | 0.334 | 0.35 | 0.316 |
| A_22_ | RUNE | Thorchain | 0.007 | 0.008 | 0.007 | 0.004 | 0.007 |
| A_23_ | TON | TON | 0.239 | 1.7 | 4 | 7 | 3.235 |
| A_24_ | ADA | Cardano | 3.82 | 3.95 | 4.19 | 4.08 | 4.010 |
| A_25_ | GNO | Gnosis | 0.128 | 0.095 | 0.119 | 0.051 | 0.098 |
| A_26_ | AAVE | Aave | 0.073 | 0.174 | 0.137 | 0.1 | 0.121 |
| A_27_ | AR | Arweave | 0.143 | 0.15 | 0.195 | 0.185 | 0.168 |
| A_28_ | DYDX | dYdX | 0.521 | 0.68 | 0.49 | 0.74 | 0.608 |
| A_29_ | NEAR | Near | 2.4 | 2.6 | 3.1 | 3.5 | 2.900 |
| A_30_ | 1INCH | 1inch | 0.473 | 0.536 | 0.654 | 0.346 | 0.502 |
| A_31_ | ROSE | Oasis | 3.851 | 3.726 | 3.95 | 4.029 | 3.889 |
| A_32_ | SEI | Sei | 3.12 | 3.94 | 4.12 | 4.6 | 3.945 |
| A_33_ | ONE | Harmony | 0.024 | 0.036 | 0.045 | 0.058 | 0.041 |
| A_34_ | MANA | Decentraland | 1.23 | 1.54 | 1.26 | 1.33 | 1.340 |
| A_35_ | KAVA | Kava | 1.523 | 1.422 | 1.621 | 1.544 | 1.528 |
| A_36_ | RON | Ronin | 0.717 | 2.7 | 3.1 | 3.2 | 2.429 |
| A_37_ | VET | Vechain | 3.765 | 3.955 | 4.1 | 4.183 | 4.001 |
| A_38_ | LTC | Litecoin | 2.548 | 2.9 | 3.1 | 3.66 | 3.052 |
| A_39_ | EOS | EOS | 1.42 | 1.62 | 1.52 | 1.539 | 1.525 |
| A_40_ | CELO | Celo | 0.661 | 0.713 | 1.2 | 1.2 | 0.944 |
| A_41_ | FTM | Fantom | 0.443 | 0.571 | 0.372 | 0.495 | 0.470 |
| A_42_ | EGLD | MultiversX | 0.013 | 0.012 | 0.021 | 0.035 | 0.020 |
| A_43_ | STX | Stacks | 1.1 | 1 | 1.24 | 1.2 | 1.135 |
| A_44_ | XMR | Monero | 0.648 | 0.24 | 0.345 | 0.562 | 0.449 |
| A_45_ | ATOM | Cosmos | 0.398 | 0.507 | 0.281 | 0.24 | 0.357 |
| A_46_ | XTZ | Tezos | 1.56 | 1.24 | 1.58 | 1.6 | 1.495 |
| A_47_ | ALGO | Algorand | 1.23 | 1.3 | 1.2 | 1.36 | 1.273 |

Source: Authors’ own compilation/computation

**Appendix 15:**

As we mentioned, the “Circulating/Total Supply (Billion)” data were not readily available and required further calculations. In this appendix, we provide a detailed breakdown of these calculations. For each asset, we collected data for both Circulating and Total Supply, and then we calculated the ratio of Circulating to Total Supply, which is reflected in the final column. Here is the summarized data:

**Table A2.** Calculation of “Circulating/Total Supply” for each asset

| Alternative | | | Circulating | Total Supply | C/T |
| --- | --- | --- | --- | --- | --- |
| A_1_ | ETH | Ethereum | 0.120 | 0.120 | 1.000 |
| A_2_ | SOL | Solana | 0.400 | 0.512 | 0.782 |
| A_3_ | TRX | Tron | 90.000 | 100.851 | 0.892 |
| A_4_ | BNB | BSC (Binance) | 0.155 | 0.200 | 0.775 |
| A_5_ | BTC | Bitcoin | 0.019 | 0.021 | 0.905 |
| A_6_ | LINK | Chainlink | 0.500 | 1.000 | 0.500 |
| A_7_ | ARB | Arbitrum | 1.275 | 10.000 | 0.128 |
| A_8_ | SUI | Sui | 0.528 | 10.000 | 0.053 |
| A_9_ | AVAX | Avalanche | 0.350 | 0.720 | 0.486 |
| A_10_ | POL | Polygon | 9.300 | 10.000 | 0.930 |
| A_11_ | CRV | Curve | 0.750 | 3.303 | 0.227 |
| A_12_ | APT | Aptos | 0.200 | 1.000 | 0.200 |
| A_13_ | OP | Optimism | 0.315 | 4.295 | 0.073 |
| A_14_ | CORE | CORE | 0.425 | 2.100 | 0.202 |
| A_15_ | UNI | Uniswap | 0.753 | 1.000 | 0.753 |
| A_16_ | MNT | Mantle | 3.200 | 6.219 | 0.515 |
| A_17_ | CRO | Cronos | 25.260 | 30.263 | 0.835 |
| A_18_ | ONDO | Ondo | 1.390 | 10.000 | 0.139 |
| A_19_ | NUM | Numbers | 0.250 | 1.000 | 0.250 |
| A_20_ | CAKE | Pancakeswap | 0.200 | 0.200 | 1.000 |
| A_21_ | MKR | Maker | 0.001 | 0.001 | 0.972 |
| A_22_ | RUNE | Thorchain | 0.330 | 0.500 | 0.660 |
| A_23_ | TON | TON | 1.220 | 5.000 | 0.244 |
| A_24_ | ADA | Cardano | 35.000 | 45.000 | 0.778 |
| A_25_ | GNO | Gnosis | 0.003 | 0.010 | 0.260 |
| A_26_ | AAVE | Aave | 0.014 | 0.016 | 0.875 |
| A_27_ | AR | Arweave | 0.050 | 0.066 | 0.758 |
| A_28_ | DYDX | dYdX | 0.065 | 1.000 | 0.065 |
| A_29_ | NEAR | Near | 0.900 | 1.000 | 0.900 |
| A_30_ | 1INCH | 1inch | 0.600 | 1.500 | 0.400 |
| A_31_ | ROSE | Oasis | 5.700 | 10.000 | 0.570 |
| A_32_ | SEI | Sei | 1.800 | 10.000 | 0.180 |
| A_33_ | ONE | Harmony | 12.300 | 13.156 | 0.935 |
| A_34_ | MANA | Decentraland | 1.800 | 2.194 | 0.820 |
| A_35_ | KAVA | Kava | 0.150 | 0.200 | 0.750 |
| A_36_ | RON | Ronin | 0.150 | 1.000 | 0.150 |
| A_37_ | VET | Vechain | 72.700 | 86.713 | 0.838 |
| A_38_ | LTC | Litecoin | 0.073 | 0.084 | 0.869 |
| A_39_ | EOS | EOS | 0.980 | 1.000 | 0.980 |
| A_40_ | CELO | Celo | 0.500 | 1.000 | 0.500 |
| A_41_ | FTM | Fantom | 2.800 | 3.175 | 0.882 |
| A_42_ | EGLD | MultiversX | 0.025 | 0.031 | 0.796 |
| A_43_ | STX | Stacks | 1.300 | 1.818 | 0.715 |
| A_44_ | XMR | Monero | 0.018 | 0.018 | 1.000 |
| A_45_ | ATOM | Cosmos | 0.292 | 0.292 | 1.000 |
| A_46_ | XTZ | Tezos | 0.940 | 0.940 | 1.000 |
| A_47_ | ALGO | Algorand | 7.800 | 10.000 | 0.780 |

Source: Authors’ own compilation/computation

**Appendix 16:**

**Table A3.** Overview of combined results based on mean rank, Borda count, and Copeland approaches

| Alternative | | | Ranked Based on Mean Rank | Ranked Based on Borda Count | Ranked Based on Copeland |
| --- | --- | --- | --- | --- | --- |
| A_1_ | ETH | Ethereum | 1 | 1 | 1 |
| A_2_ | SOL | Solana | 3 | 4 | 4 |
| A_3_ | TRX | Tron | 5 | 5 | 5 |
| A_4_ | BNB | BSC (Binance) | 2 | 2 | 2 |
| A_5_ | BTC | Bitcoin | 4 | 3 | 3 |
| A_6_ | LINK | Chainlink | 45 | 42 | 42 |
| A_7_ | ARB | Arbitrum | 12 | 13 | 13 |
| A_8_ | SUI | Sui | 6 | 6 | 6 |
| A_9_ | AVAX | Avalanche | 27 | 23 | 23 |
| A_10_ | POL | Polygon | 7 | 7 | 7 |
| A_11_ | CRV | Curve | 33 | 34 | 34 |
| A_12_ | APT | Aptos | 14 | 14 | 14 |
| A_13_ | OP | Optimism | 32 | 32 | 32 |
| A_14_ | CORE | CORE | 39 | 38 | 38 |
| A_15_ | UNI | Uniswap | 15 | 8 | 8 |
| A_16_ | MNT | Mantle | 42 | 44 | 44 |
| A_17_ | CRO | Cronos | 10 | 8 | 8 |
| A_18_ | ONDO | Ondo | 16 | 16 | 16 |
| A_19_ | NUM | Numbers | 46 | 46 | 46 |
| A_20_ | CAKE | Pancakeswap | 37 | 36 | 36 |
| A_21_ | MKR | Maker | 9 | 10 | 10 |
| A_22_ | RUNE | Thorchain | 42 | 42 | 42 |
| A_23_ | TON | TON | 18 | 17 | 17 |
| A_24_ | ADA | Cardano | 13 | 11 | 11 |
| A_25_ | GNO | Gnosis | 20 | 17 | 17 |
| A_26_ | AAVE | Aave | 30 | 24 | 24 |
| A_27_ | AR | Arweave | 41 | 41 | 41 |
| A_28_ | DYDX | dYdX | 47 | 47 | 47 |
| A_29_ | NEAR | Near | 11 | 14 | 14 |
| A_30_ | 1INCH | 1inch | 44 | 45 | 45 |
| A_31_ | ROSE | Oasis | 34 | 32 | 32 |
| A_32_ | SEI | Sei | 35 | 34 | 34 |
| A_33_ | ONE | Harmony | 17 | 19 | 19 |
| A_34_ | MANA | Decentraland | 40 | 40 | 40 |
| A_35_ | KAVA | Kava | 28 | 30 | 30 |
| A_36_ | RON | Ronin | 29 | 24 | 24 |
| A_37_ | VET | Vechain | 21 | 22 | 22 |
| A_38_ | LTC | Litecoin | 19 | 20 | 20 |
| A_39_ | EOS | EOS | 25 | 26 | 26 |
| A_40_ | CELO | Celo | 26 | 26 | 26 |
| A_41_ | FTM | Fantom | 24 | 20 | 20 |
| A_42_ | EGLD | MultiversX | 38 | 38 | 38 |
| A_43_ | STX | Stacks | 36 | 37 | 37 |
| A_44_ | XMR | Monero | 30 | 31 | 31 |
| A_45_ | ATOM | Cosmos | 22 | 26 | 26 |
| A_46_ | XTZ | Tezos | 22 | 26 | 26 |
| A_47_ | ALGO | Algorand | 8 | 12 | 12 |

Source: Authors’ own computation
