# Supplementary material for: A two-stage framework for enhancing crsyptocurrency portfolio performance: Integrating credibilistic CVaR criterion with a novel asset preselection approach
Source: PLoS One. 2025 Jul 21;20(7):e0325973. doi: 10.1371/journal.pone.0325973 (PMC12279154; doi:10.1371/journal.pone.0325973)
Supplement: Attachment — Submitted filename: Response to Reviews.docx [file pone.0325973.s003.docx]

***COVER LETTER FOR*** ***RESUBMISSION OF MANUSCRIPT***

Dear Editor

Prof. Dr. Emily Chenette

We are enclosing herewith a revised version of our manuscript entitled *“A Two-Stage Framework for Enhancing Cryptocurrency Portfolio Performance: Integrating Credibilistic CVaR Criterion with a Novel Asset Preselection Approach”* for possible publication in PLOS One.

The reviewer comments were highly insightful and enabled us to considerably enhance the quality of our manuscript. In the following pages are our point-by-point responses to each of the comments of the reviewer. Moreover, new discussions and modifications in some formulas and tables have been done to eliminate the previous document's ambiguities and inaccuracies.

We shall look forward to hearing from you at your earliest convenience.

Sincerely yours,

*Emran Mohammadi, Associate Professor*

**Title of the paper:** *A Two-Stage Framework for Enhancing Cryptocurrency Portfolio Performance: Integrating Credibilistic CVaR Criterion with a Novel Asset Preselection Approach*

First of all, the authors would like to thank all the anonymous reviewers and the Associate Editor again for their efforts and valuable time to review and improve our paper. Taking into account their constructive suggestions and comments, the paper has been carefully revised following the referees’ comments.

We provide below the responses to each referee's comments. All amends and changes in the revised version are yellow highlighted.

**Responses to the comments of the reviewers and editors:**

| **Editor** | |
| --- | --- |
| **Response** | First of all, the authors would like to thank all the anonymous reviewers and the Associate Editor again for their efforts and valuable time to review and improve our paper. |
| **Action** | Taking into account their constructive suggestions and comments, the paper has been carefully revised following the referees’ comments. |
| **Reviewer 1** | |
| **(1)** | ***1. There are two recent studies that substantially extend the extent literature on cryptocurrencies. Ali, Khurram, and Sensoy (2025) indicate the importance of cryptocurrencies in portfolio optimization (risk reduction), whereas Ali, Du, and Majeed (2025) indicate the importance of matching trading periods while estimating intermarket interconnectedness. While the former will help the authors to improve the first paragraph of the Introduction Section, the latter will help the authors to improve the second paragraph.*** [***https://doi.org/10.1186/s40854-024-006864***](https://doi.org/10.1186/s40854-024-006864)***;***  [***https://doi.org/10.1016/j.frl.2025.107016***](https://doi.org/10.1016/j.frl.2025.107016) |
| **Response** | Thank you for your insightful comments and for suggesting such relevant papers. We truly appreciate the references you provided. |
| **Action** | In response to your suggestions, we have incorporated the four recommended studies into the Introduction section, ensuring they align with and strengthen our discussion. These changes are highlighted in yellow for your convenience. We believe these additions significantly enhance the clarity and depth of our work.  Once again, thank you for your valuable input, which has greatly contributed to improving the quality of the paper. |
| **(2)** | ***2. The authors have discussed dome seminal studies that have employed VaR and CVaR; however, it is important to mention studies that have recently employed these methods. In the wake of consistently evolving econometric tools to understand diversification, it is fundamental to discuss recent high-quality work that has used this method. I suggest taking guidance from the following study to strengthen the use of the VaR and CVaR methods (***[***https://doi.org/10.1016/j.rser.2023.114137***](https://doi.org/10.1016/j.rser.2023.114137) ***&*** [***https://doi.org/10.1016/j.ribaf.2022.101768***](https://doi.org/10.1016/j.ribaf.2022.101768) ***).*** |
| **Response** | Thank you for your insightful comments and for suggesting such relevant papers. We truly appreciate the references you provided. |
| **Action** | In response to your suggestions, we have incorporated the four recommended studies into the Introduction section, ensuring they align with and strengthen our discussion. These changes are highlighted in yellow for your convenience. We believe these additions significantly enhance the clarity and depth of our work.  Once again, thank you for your valuable input, which has greatly contributed to improving the quality of the paper. |
| **(3)** | ***3. The above four studies will also help the authors to improve the Literature Review section of the study.*** |
| **Response** | Thank you for suggesting such relevant papers. We appreciate the recommendations. |
| **Action** | In response to your comment, we have referenced the study “Green cryptocurrencies and portfolio diversification in the era of greener paths” in Section 2.1 on Cryptocurrency Portfolio Optimization, as it aligns well with the themes of this section. Specifically, we have added the following content on page 6:  Ali et al. explored the diversification benefits of green cryptocurrencies in the context of global portfolio optimization, introducing a novel four-step process to identify cryptocurrencies that are more energy-efficient than others. This study highlights the growing concern over the environmental impact of cryptocurrencies, particularly energy-intensive mining practices. By focusing on energy-efficient cryptocurrencies such as Cardano (ADA), Tezos (XTZ), and Stellar (XLM), the authors demonstrate how these green cryptocurrencies can serve as effective diversifiers for portfolios, offering benefits comparable to or even superior to their non-green counterparts like Bitcoin (BTC) and Ethereum (ETH). The research employed a variety of advanced econometric models, including dynamic conditional correlation (DCC-GARCH) and four-moment modified VaR, to evaluate the downside risk and expected shortfall of portfolios containing both green and non-green cryptocurrencies.  Once again, thank you for your thoughtful suggestions, which have helped enhance the quality of the paper. |
| **(4), (5)** | ***4. Sections 3 and 4 occupy nearly 80% of the manuscript size, indicating sections are not well-distributed.***  ***5. Please reduce the size of the study; it is very lengthy and hard to keep interest while reading it.*** |
| **Response** | Thank you for your valuable feedback regarding the length and distribution of the manuscript. We appreciate your suggestion to reduce the size of the study for better readability. |
| **Action** | As this framework contains two stages, with each stage involving numerous computations and models, we felt it necessary to carefully introduce and explain them in detail. For Stage 1, we utilized 13 MADM methods, each of which involves several components. For Stage 2, we integrated credibility theory with CVaR, which involves its own mathematical framework. However, we understand your concern about the manuscript being lengthy, so we have worked to address this.  In response to your comment, we have reduced the size of Sections 3 and 4 by moving some tables to the supplementary section. Additionally, we made changes to certain parts of these sections to improve their conciseness, and these changes are highlighted in yellow for your convenience. It’s also important to note that a significant portion of the content is now in the supplementary material, and the main paper itself is approximately 40 pages long. We hope this adjustment improves the overall readability of the manuscript while maintaining the integrity of the study.  Thank you once again for your helpful suggestions. |
| **(6)** | ***6. The authors need to discuss the limitations of the study.*** |
| **Response** | Thank you for your insightful comment regarding the limitations of the study. We appreciate your suggestion to address this aspect more thoroughly.  In response to your feedback, we have included a more detailed discussion of the limitations in the revised manuscript. |
| **Action** | This text was added in page 43 and 44:  While the two-stage framework presented in this paper offers a structured and innovative approach to cryptocurrency portfolio optimization, several limitations should be acknowledged. First, the methodology relies on the availability of high-quality and consistent data, which can be a significant challenge in the cryptocurrency market due to its rapid evolution and volatility. Data gaps, especially in newly launched cryptocurrencies, may affect the accuracy of the asset pre-selection process. Additionally, while the integration of credibility theory with CVaR improves risk management, it may not account for all potential market dynamics, particularly during extreme market events. Furthermore, the framework also assumes that investors have access to sufficient computational resources and expertise to implement the methodologies effectively, which may limit its applicability to some investors. Lastly, while the proposed methods have been demonstrated in the context of cryptocurrency, further research is needed to test their effectiveness across other asset classes and market conditions. These limitations suggest that while the framework provides valuable insights, its real-world application may require adaptation to specific contexts and additional refinements. |
| **(7)** | ***7. Finally, discuss some practical implications of the study.*** |
| **Response** | Thank you for your valuable comment regarding the practical implications of the study.  In response, we have added a section discussing the practical implications of our study. This includes how the proposed framework for cryptocurrency portfolio optimization, especially the asset preselection method, can be utilized by investors, portfolio managers, and policymakers. We highlight how the methodology can guide more informed decision-making in portfolio construction, risk management, and asset selection, particularly in the rapidly evolving cryptocurrency market. Additionally, we discuss how our findings can contribute to the development of more robust investment strategies and improve the management of cryptocurrencies as part of diversified portfolios. |
| **Action** | This text was added in page 44 and 45:  Therefore, the two-stage framework presented in this paper offers a structured and comprehensive pathway for enhancing cryptocurrency portfolio performance. By focusing on high-quality pre-selection in the first stage and sophisticated optimization in the second, this framework provides invaluable insights for investors seeking to navigate the intricacies of the cryptocurrency market. This holistic approach not only aids in optimizing investment strategies but also prepares investors to respond effectively to the challenges posed by market volatility and uncertainty. Furthermore, the methodologies introduced in this paper have broad applicability beyond cryptocurrency, providing valuable insights for asset selection processes across various investment domains. The principles of the proposed two-stage framework can be seamlessly applied to traditional financial markets, such as stocks, bonds, and alternative investments, thereby enhancing the asset selection process and optimizing portfolio construction in these markets as well. By integrating systematic pre-selection with advanced optimization techniques, this framework equips investors and managers with powerful tools for better decision-making, ensuring that portfolios are aligned with both risk and return objectives. |
| **Reviewer 2** | |
| **(1)** | ***It is advisable to conduct comparative experiments by comparing with other methods applicable to cryptocurrency asset pre - selection, so as to highlight the advantages and innovativeness of the method proposed in this paper.*** |
| **Response** | Thank you for your insightful suggestion regarding comparative experiments with other methods applicable to cryptocurrency asset preselection. |
| **Action** | As far as we are aware, there is no existing study that specifically addresses asset preselection in the context of cryptocurrency portfolio optimization. We have highlighted this gap multiple times in the paper, including on page 6 (highlighted in blue) and in Section 2.4 (Research Gap), where we point out the absence of asset preselection in cryptocurrency portfolio optimization. In particular, we emphasize the lack of cryptocurrency performance criteria, which are essential for evaluating cryptocurrencies prior to portfolio construction. To address this, we propose a comprehensive set of performance criteria, reflecting fundamental, technical, and market-driven factors relevant to digital asset evaluation, which we also highlighted in blue.  While there are studies on asset preselection in the context of stocks using methods like DEA, MADM, and machine learning/deep learning, these approaches are not yet directly used for cryptocurrencies. Therefore, we view these methods as an avenue for future research and suggest exploring them as part of expanding the cryptocurrency portfolio optimization framework. We appreciate your suggestion and will certainly consider it in future studies to further enhance the methodology.  We performed a comprehensive review of studies on portfolio optimization with asset preselection (section 2.2.), and it is evident that all of them focus on stocks, with cryptocurrency being notably absent. Given this gap in the literature, we see opportunities for future research to explore asset preselection for other asset classes, such as funds, other digital assets, or fixed incomes, which could further broaden the applicability of this approach. (Mentioned as another suggestion for future research direction.) |
| **(2)** | ***In the dataset, the data of the asset ONDO only covers 330 days, which is different from the 379 - day data of other assets. This may seriously affect the reliability of statistical analysis and the universality of conclusions.*** |
| **Response** | Thank you for your valuable comment regarding the dataset for the asset ONDO. We appreciate your attention to the potential impact of this discrepancy on the reliability of our statistical analysis.  *It is important to highlight that the descriptive statistics for all assets were computed based on a dataset comprising 379 days of data. However, the asset ONDO presents a slight deviation, as it has only been available for 330 days (the asset was launched 330 days ago).*  *In Stage 1, we used the average for all criteria, which helps to mitigate the impact of this slight deviation. As the criteria are averaged across the full available dataset, the difference in the number of data points between ONDO and the other assets (49 days) will have a minimal* *impact on the overall analysis. This averaging process ensures that the results remain robust, even with the slight deviation in the data for ONDO.*  *In Stage 2, we employed fuzzy theory, which is an uncertainty approach specifically designed for situations like this, where data might be incomplete or unavailable. Fuzzy theory allows for more flexible handling of uncertain or missing data and provides a way to account for these discrepancies without compromising the reliability of the analysis.*  That said, since the difference between 330 and 379 days is relatively small (just 49 days), we believe this deviation can be neglected for the purposes of this study. The gap of 49 days is unlikely to significantly distort the overall trends or findings in the portfolio optimization model, and including ONDO still provides valuable insights (Moreover, given that cryptocurrency markets are known for their volatility, this slight discrepancy in the dataset length is relatively common in emerging assets, and it is unlikely to have a major impact on the conclusions drawn from the analysis). Furthermore, given the nature of emerging assets like ONDO, the shorter data period is a common issue, and we consider its inclusion important for the scope and future relevance of the study.  We have addressed this in the paper by clearly noting this discrepancy and ensuring that it does not affect the reliability of the results drawn from the data. We have also emphasized that removing the asset could limit the scope of the analysis, particularly in the context of the many portfolio optimization studies that face similar data limitations when dealing with newly launched assets.  Thank you once again for your careful review, and we hope this clarification helps to address your concern. |
| **Action** | This text was added in page 15:  It is important to highlight that the descriptive statistics for all assets were computed based on a dataset comprising 379 days of data. However, the asset ONDO presents a slight deviation, as it has only been available for 330 days (the asset was launched 330 days ago). This discrepancy is worth noting, as it may affect the reliability of the statistical analysis for ONDO compared to the other assets included in the study.  However, since the difference between 330 and 379 days is relatively small, we believe this deviation can be neglected for the purposes of this study. The difference of just 49 days is unlikely to significantly distort the overall trends or findings. Therefore, we think it is beneficial to consider ONDO as an option rather than removing it entirely, as its inclusion may offer valuable insights, particularly as the asset develops further and additional data becomes available. Removing it could limit the scope of our analysis, especially considering that many portfolio optimization studies may encounter similar data limitations with newly launched assets. |
| **(3)** | ***When constructing the Credibilistic CVaR model, the trapezoidal fuzzy variables are obtained based on expert opinions. It is necessary to consider combining market data and objective algorithms to determine the fuzzy variables, or adding multiple sets of expert opinions for comparative analysis to reduce subjectivity and enhance the objectivity and accuracy of the model.*** |
| **Response** | Thank you for your valuable suggestion regarding the incorporation of market data and multiple expert opinions in the Credibilistic CVaR model. |
| **Action** | We would like to clarify that the trapezoidal fuzzy variables used in the model are indeed based on expert opinions; however, these opinions are not arbitrary but are formulated based on the available market data. The expert's judgment is informed by the data they have at hand, ensuring that the opinions are grounded in objective market conditions rather than solely subjective views. Moreover, the framework we have proposed is designed to be flexible and optional in nature. Investors have the choice to rely on a single expert or to incorporate multiple experts' opinions, and they may assign different weights to each expert to reflect varying levels of confidence or expertise. This provides a customizable approach to enhance the robustness of the decision-making process.  *It is important to note that there is no difference in the calculation process when using one or multiple experts. The only distinction lies in the input data—the expert opinions—that are used to define the fuzzy variables. In both cases, the model’s underlying calculations remain the same, with the variability introduced by the differences in the expert inputs. This ensures consistency in the model's application, regardless of whether a single expert or multiple experts are involved.*  For future research, we also suggest exploring the use of Z-numbers to assess the credibility of each expert's opinion. Since the reliability of an expert's judgment is not uniform and varies based on their experience and knowledge, incorporating this assessment could further strengthen the model. We also recognize this limitation within the current study and see it as an important avenue for future work to refine the approach.  Once again, thank you for your thoughtful feedback, which has helped us clarify and highlight potential directions for future research.  Page 45:  Furthermore, the reliability of expert opinions in gathering fuzzy data is another critical aspect to consider. Expert insights are often essential in shaping investment decisions, particularly in uncertain environments like cryptocurrency markets. However, the subjective nature of these opinions can introduce bias. By utilizing Z-number theory, researchers could develop a more structured framework for incorporating expert insights, mitigating potential biases while enhancing the credibility of the data. |
| **(4)** | ***When presenting the results of asset pre - selection and portfolio optimization, the economic significance and practical application value of the results are insufficiently elaborated.*** |
| **Response** | Thank you for your valuable feedback regarding the economic significance and practical application value of the results.  We appreciate your suggestion to elaborate further on these aspects. In response, we have expanded the discussion to better highlight the economic implications and practical applications of our asset preselection and portfolio optimization approach. |
| **Action** | This text was added in page 40:  The categorization of assets into distinct groups enhances clarity and provides a clear guide for asset evaluation. Each category—excellent, satisfactory, average, below average, and poor—has its own economic significance. The “excellent” category represents the top-performing assets, indicating their strong potential for high returns and stability, making them ideal candidates for inclusion in a portfolio focused on maximizing performance. On the other hand, assets categorized as “poor” highlight those with lower expected returns or higher associated risks, serving as a signal for investors to avoid or limit exposure. By utilizing these categories, investors can align their portfolios more effectively with their financial goals, risk preferences, and market conditions.  In the subsequent steps of our analysis, we will focus on the assets classified as excellent (Top 10 assets), as this category contains high-quality assets with strong potential for superior performance. By concentrating on these high-quality assets, we have effectively conducted a preselection of the leading cryptocurrency options, ensuring that only the most promising candidates are considered for our investment strategy. This targeted focus on high-quality assets allows for a more refined and informed investment approach, ensuring that the portfolio remains aligned with the investor’s risk-return objectives. |
| **(5)** | ***In addition to the mentioned uncertainties and market volatility, the significant impacts of regulatory policy changes, technological innovations, and other factors on the cryptocurrency market should also be considered.*** |
| **Response** | Thank you for your valuable feedback regarding the consideration of regulatory policy changes, technological innovations, and other factors in the cryptocurrency market. |
| **Action** | We would like to clarify that all of these factors, including regulatory policy changes, technological advancements, and market dynamics, were indeed considered within the expert opinions used to define the trapezoidal fuzzy variables in our model. Since these expert opinions are based on available market data, they inherently account for such factors and reflect the expert’s understanding of their potential impacts on the cryptocurrency market. However, we acknowledge that the one factor not explicitly considered in the model is extreme market events, such as sudden market crashes or black swan events, which can significantly impact asset prices and portfolio performance.  We recognize this as a limitation and have addressed it in the limitations section of the paper, where we highlight the potential impact of such events on the accuracy and reliability of the model's predictions. These extreme events, while unpredictable, are a crucial consideration in the real-world application of cryptocurrency portfolio optimization, and future research could explore methodologies to account for such occurrences more effectively.  Page 44:  Additionally, while the integration of credibility theory with CVaR improves risk management, it may not account for all potential market dynamics, particularly during extreme market events.  Furthermore, we acknowledged that:  Page 45:  It is crucial to acknowledge that digital assets, especially cryptocurrencies, are continually evolving and are marked by substantial volatility and inherent risks. The value of most cryptocurrencies is largely influenced by speculative trading, where market sentiment is a key driver. Therefore, investors must exercise caution and perform comprehensive due diligence before making investment decisions in the cryptocurrency market, fully understanding its speculative nature and the various factors that can impact market dynamics. |
| **(6)** | ***Although a review of relevant literature has been carried out, the exploration in some fields is not in - depth enough. It is necessary to further analyze the limitations of existing research more deeply and clearly explain how this research can address these issues specifically, highlighting the necessity and innovativeness of the research.***  ***It is recommended to supplement the following literature (not mine)***  ***10.1109/TCSI.2024.3523339 10.1109/TII.2024.3495785*** |
| **Response** | Thank you for your insightful comment regarding the limitations of the study. We appreciate your suggestion to address this aspect more thoroughly.  In response to your feedback, we have included a more detailed discussion of the limitations in the revised manuscript. |
| **Action** | This text was added in page 43 and 44:  While the two-stage framework presented in this paper offers a structured and innovative approach to cryptocurrency portfolio optimization, several limitations should be acknowledged. First, the methodology relies on the availability of high-quality and consistent data, which can be a significant challenge in the cryptocurrency market due to its rapid evolution and volatility. Data gaps, especially in newly launched cryptocurrencies, may affect the accuracy of the asset pre-selection process. Additionally, while the integration of credibility theory with CVaR improves risk management, it may not account for all potential market dynamics, particularly during extreme market events. Furthermore, the framework also assumes that investors have access to sufficient computational resources and expertise to implement the methodologies effectively, which may limit its applicability to some investors. Lastly, while the proposed methods have been demonstrated in the context of cryptocurrency, further research is needed to test their effectiveness across other asset classes and market conditions. These limitations suggest that while the framework provides valuable insights, its real-world application may require adaptation to specific contexts and additional refinements. |
